# Morphological and molecular characterization of Nepalese Common Bean (*Phaseolus vulgaris* L.) Landraces

**DOI:** 10.1371/journal.pone.0354479

**Published:** 2026-07-30

**Authors:** Pradip Thapa, Sandip Bohara, Promise Shrestha, Sanjay Kumar Raut, Bal Krishna Joshi

**Affiliations:** 1 National Agriculture Genetic Resources Centre, Lalitpur, Nepal; 2 Far Western University, Tikapur, Nepal; 3 Agriculture and Forestry University, Chitwan, Nepal; National Cheng Kung University, TAIWAN

## Abstract

Nepalese common bean landraces constitute valuable yet underexplored genetic reservoirs for crop improvement. However, integrated morphological and molecular characterization of this germplasm remains limited. This experiment was conducted at 2023 and 2024 to evaluate the morphological and molecular traits of 58 Nepalese common bean landraces collected from 21 districts of the Nepal via an augmented block design. Twenty-three quantitative and twenty-four qualitative traits were measured and analyzed via descriptive and multivariate methods. The Shannon-Weaver diversity indices (H’) ranged from 0.085–0.99 for qualitative traits and 0.46–0.92 for quantitative traits. The five principal components (PCs) explained 70.86% of the total phenotypic variation, with two PCs explaining 46.27% of the variation. Phenotypic cluster analysis grouped the 58 accessions into 5 distinct groups. Molecular analysis via 15 SSR markers revealed 38 alleles, with an average of 3.16 alleles per marker. The PIC value ranged from 0.16 to 0.46. The genotypic dendrogram grouped the 58 landraces into 3 clusters. The first two molecular principal coordinates accounted for 43.27% of the variation among the accessions. The analysis of molecular variance revealed a maximum variation of 93% within the population and only 7% variation among the populations. Population structure analysis revealed three genetic clusters, with most accessions showing admixture with moderate heterozygosity and a high fixation index. The accessions NGRCO4168 and NGRCO9516 were identified as promising accessions for traits including days to 50% flowering, days to 50% fruiting, maturity, and yield (t/ha). These accessions should be further evaluated across diverse environments to assess their stability and adaptability. Additionally, expanded SSR or SNP analyses integrated with phenotypic data could facilitate marker–trait association studies aimed at identifying genomic regions linked to key agronomic traits.

## 1. Introduction

The common bean (*Phaseolus vulgaris* L.) is an annual, herbaceous, self-pollinating legume crop species that belongs to the family Fabaceae [1,2]. It is a diploid crop and has 11 pairs of chromosomes (2n = 2x = 22), with a genomic size of 587 Mb [3]. The Leguminosae family ranks as the second most important group of agricultural crops, following the Poaceae family [4]. The origin of the bean dates back to 8,000–10,000 years ago through the domestication of wild bean from the Mesoamerican (Mexico, Central America, and Colombia) and Andean (southern Peru, Bolivia, and Argentina) gene pools [5–7]. Six races of the common bean have been identified within these gene pools: three were Andean gene pool races (Chile, Peru, and Nueva Granada) and three Mesoamerican gene pool races (Durango, Mesoamerican, and Jalisco). These races have been differentiated through molecular approaches using biochemical markers, such as RAPD and SSR, and morphological characterization [6,7]. Geographical and reproductive barriers partially isolated the centers of genetic diversity in the Mesoamerican and Andean regions of origin [5]. It is estimated that the divergence between these gene pools occurred approximately 165,000 years ago [3]. The Mesoamerican gene pool has greater genetic diversity compared to the Andean gene pool [8]. The Andean gene pool is characterized by heavier seeds (>40 g 100-seed ^−^ ^1^) with ‘T,’ ‘C,’ ‘H,’ and ‘A’ phaseolin types, whereas the Mesoamerican pool has lighter (<25 g) or medium-sized seeds with ‘S’ or ‘B’ phaseolin types [5,9,10,]. Distinct physical traits separate the Andean and Mesoamerican gene pools. Andean beans have larger leaves, lanceolate or triangular bracteoles, white flowers, and longer internodes, whereas Mesoamerican varieties are characterized by cordate or ovate bracteoles, a placental pod beak, and colorful, striped flowers [6,11]. The genus *Phaseolus* comprises more than 70 species, five of which, runner bean (*P. coccineus* L.), lima bean (*P. lunatus* L.), tepary bean (*P. acutifolius* A. Gray), year bean (*P. dumosus* Macfady), and common bean (*P. vulgaris* L.) have been domesticated in various ecogeographic regions [12]. Among the five domesticated varieties worldwide, the common bean is the most extensively consumed grain legume in the world and makes up approximately 90% of cultivated crops [13].

The common bean provides up to 15% of total daily calories and 36% of total daily protein in parts of Africa and the Americas [3,14]. Over 200 million people in sub-Saharan Africa rely on the common bean as a primary dietary staple [3]. The global area, yield, and productivity of common bean are 35345967 ha, 16.66 (t/ha) and 56208663 tons, respectively [15]. Asia was the dominant contributor to global dry bean production, accounting for 46.8% of the total output, followed by Africa (26.1%) and the Americas (25%). In contrast, green bean production was highly concentrated in Asia, which contributed 92.2% of the global total, whereas Europe and Africa accounted for comparatively smaller shares of 4% and 2.6%, respectively [15]. Beans were grown in 126 countries, and in 2024, India was the largest producer, accounting for 26 percent of global output, followed by Brazil (10%), Myanmar (8%), the United States of America (5%), and the United Republic of Tanzania (5%) [16]. In Nepal, beans are cultivated in an area of 21773 ha, with a production of 18787.4 tones and a yield of 3.71 t/ha [15].

Beans are a major source of plant-based protein in the human diet and can be consumed in various forms, including dried seeds, green pods, or immature seeds [17]. Often referred to as “poor man’s meat,” they provide an affordable and accessible source of nutrition for approximately 300 million people in tropical regions and 100 million people in Africa [18]. The *Phaseolus vulgaris* (common bean) is especially important in subsistence farming systems, where it supports food security and livelihoods, earning it the designation “grain of hope” and “meat of poor” [18–20]. Common bean has high protein (16–33%) and fiber contents; complex carbohydrates; and other nutrients, such as iron, zinc, magnesium, potassium, and folic acid, an important component of B-complex vitamins [10,21]. It is used as a traditional remedy for renal issues, diabetes, dropsy, diarrhea, and dysentery [22]. Common beans improve soil fertility by fixing atmospheric nitrogen through their symbiotic association with rhizobia and the breakdown of plant residues [23].

Landraces, also known as local genotypes, are populations produced over many years by artificial or natural selection, which is distinguished by their strong tolerance to biotic and abiotic stresses, leading to stable yields and moderate productivity within low-input farming systems [24]. Landrace and wild crop accessions are important genetic resources for plant breeding and the preservation of plant alleles and gene combinations [18]. Landraces serve as valuable sources of genetic diversity, offering traits for resistance to diseases and pests, as well as tolerance to various abiotic stresses [6]. Improved landraces perform better than the national average yield of cereal crops by 20% and 60% for minor crops [25]. As the biophysical resource base declines and climate change poses increasing threats, landraces are central to current crop breeding programs and represent a vital part of plant biodiversity in common beans [26]. The world’s population is predicted to grow at a high rate to 10 billion people by 2050. Therefore, to fulfill the anticipated food demand in 2050, global food production must be increased by 60% to 110% [27]. The National Agriculture Genetic Resources Centre (NAGRC) has conserved a total of 719 pulse crop accessions, 93 of which are bean accessions [25]. Nepal has a total of five common bean varieties, including three registered landraces and 2 hybrid varieties [28]. Characterizing the genetic diversity of common bean accessions from germplasm banks and working collections helps determine optimal genetic distances between conserved landraces, identify redundancies, and understand genetic interactions [29].

Morphological characterization of landraces is the first and foremost step to identify genetic variation [30]. It facilitates the description and comparison of genetic variability and aids in selecting diverse parents for breeding [31]. It also enables the identification of landraces with desirable agronomic traits such as early maturity, drought tolerance, pest and disease resistance, and high yield potential [32]. Morphological characterization plays a key role in conservation breeding and the prevention of genetic erosion by identifying unique or highly divergent landraces, thereby facilitating the prioritization of plant genetic material for both *in situ* and *ex situ* conservation [24]. Morphological and agronomic traits are often used to assess genetic diversity, but they are strongly influenced by the environment and developmental stage, and their limited heritability makes them unreliable indicators of true genetic relatedness among accessions [33,34]. When these DNA molecular markers are tightly associated with genes of interest, they may be utilized in marker-assisted breeding programs to select for preferred alleles [35]. Therefore, molecular markers are crucial techniques for genetic mapping and analysis of genetic diversity, population structure, and phylogenetic connections in many plants since they are not affected by ecological conditions [36]. For the characterization, conservation, and management of germplasm, molecular markers offer a more straightforward, dependable, and effective method of revealing genotype variations at the DNA level [37]. Simple sequence repeats (SSRs), or microsatellites, are PCR-based, codominant, and highly polymorphic markers (can amplify up to 25 alleles per locus) with 2–6 bp tandem repeats [2,38]. Gaitán-Solís et al. [39] developed SSR markers for common beans for the first time to evaluate the genetic diversity of cultivated and wild species. Simple sequence repeats (SSRs) are broadly classified into genic and genomic categories. Genic SSRs, also referred to as microsatellites, are located within or in close proximity to coding regions of the genome, whereas genomic SSRs are distributed in non-coding regions that are not directly associated with genes [40]. They are widely used for variety identification, genetic mapping, diversity analysis, marker-assisted selection, and population structure studies in common bean germplasm [24,41,42].

To date, there is no evidence of the molecular characterization of common bean germplasm by simple sequence repeat (SSR) markers in Nepal. Therefore, this research was carried out to assess the genetic diversity and population structure among 58 bean landraces via morphological and molecular analysis for further use in breeding and improvement programs of common bean.

## 2. Materials and methods

### 2.1. Morphological characterization

#### 2.1.1. Study area and experimental site.

**Fig 1:**
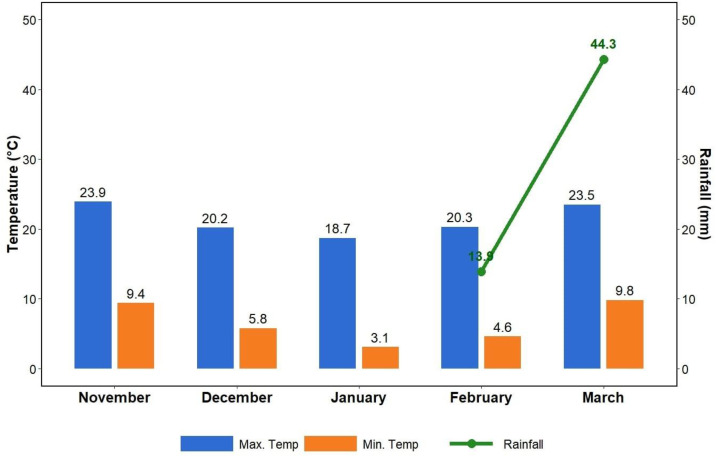
Meteorological data of the study site.

The experiment was conducted at the research field and molecular laboratory of National Agriculture Genetic Resources Center (NAGRC) Lalitpur, Nepal, during the 2023 and 2024. The site lies 1,368 m above sea level at 27°40’ N latitude and 85°20’ E longitude. During the study period (November to March), the site experienced maximum temperatures ranging from 18.7°C (January) to 23.9°C (November) and minimum temperatures ranging from 3.1°C (January) to 9.8°C (March) (Fig 1). Rainfall was low and sporadic, occurring mainly in February (13.9 mm) and March (44.3 mm). Overall, the region receives approximately 29.1 mm of rainfall, with average minimum and maximum temperatures of 6.5°C and 21.3°C, respectively.

#### 2.1.2. Plant material.

**Fig 2:**
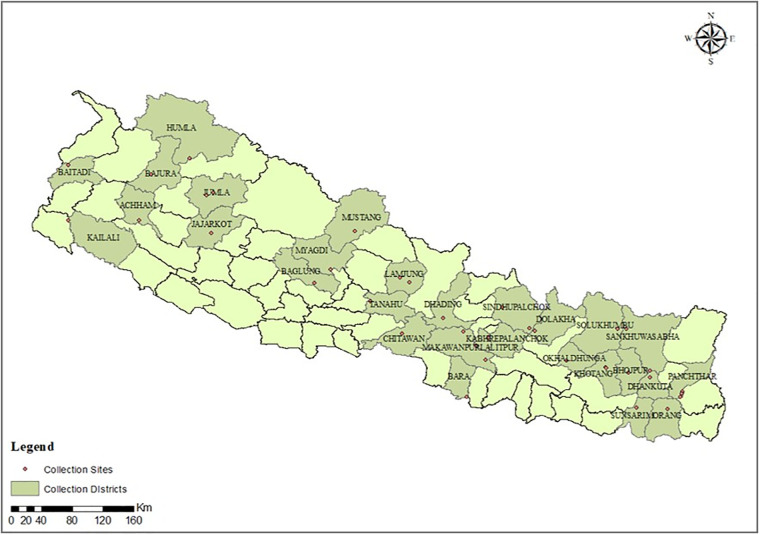
Collection sites of common bean landraces used in the experiment.

A total of 58 common bean accessions collected from 21 districts (S1 Table; Fig 2) across Nepal were included in the study for morphological and molecular assessment. Among them, 44 accessions were collected from the mid-hill region, 9 accessions were collected from the high hill regions and remaining five accessions were collected from the Terai region of Nepal.

#### 2.1.3. Statement on experimental site and plant materials.

The common bean accessions used in this study were conserved and maintained at the National Agriculture Genetic Resources Center (Nepal Genebank) Lalitpur, Nepal, and the field characterization was conducted at the research field of the same institution. No specific permits were required for field access or accession use because the study was conducted using institutional accession and facilities under the regular research activities of National Agriculture Genetic Resources Center.

#### 2.1.4. Experimental design and layout.

The experiment was conducted using an augmented block design with two replications. A total of 55 test accessions were evaluated along with three check varieties, namely NGRCO8500, NGRCO8503, and Trishuli. In each replication, the test accessions were arranged in blocks of seven entries, after which the three check varieties were systematically repeated. Thus, each block consisted of seven test accessions followed by the three checks. Each replication contained four such blocks, resulting in a total of eight blocks across the two replications. Seeds were sown on 12^th^ November, 2023. Each accession was planted in two rows, each measuring 0.9 m in width and 3 m in length. Ten seeds were sown per row, maintaining a spacing of 60 cm between rows and 30 cm between plants.

#### 2.1.5. Agronomic and crop management practices.

The experimental field was prepared through plowing and harrowing to obtain a fine tilth. Farmyard manure was applied at 10 t ha^-1^ during final land preparation. Fertilizers were applied at the recommended rate of 50:60:40 kg ha^-1^ N:P:K [28], with the full dose of phosphorus and potassium and half of the nitrogen applied basally at sowing, and the remaining nitrogen was top-dressed at 30 days after sowing (DAS). Hand weeding was carried out twice, at 25–30 DAS and 45–50 DAS. Irrigation was provided as needed, particularly during flowering and pod development. Staking at 30–35 DAS was performed via bamboo stakes to support indeterminate and semi-climbing plants. Plant protection measures were applied uniformly following need-based integrated pest management practices.

#### 2.1.6. Data collection and analysis.

Morphological data, including the 23 quantitative and 24 qualitative traits, were recorded via standard descriptors for common bean [43]. The qualitative traits were recorded based on the observation of descriptor states (S3 Table), whereas the quantitative traits were recorded by count or measurement (S2 Table). For each accession, quantitative trait data were collected from five randomly selected plants, which served as biological replicates, to capture within-accession variability under field conditions. Seed-based observations were also recorded from representative samples of each accession.

The mean quantitative data were analyzed for descriptive statistics (minimum, maximum, mean, variance, standard deviation, and coefficient of variance) via the pastecs [44] package in R Studio (4.4.3) [45]. The frequency and distribution were computed for qualitative data via package summary tools [46]. Shannon-Weaver diversity index (H′) for qualitative traits was calculated directly from observed phenotypic classes as per the descriptor states, and for quantitative traits, after classification of raw data into nine equal-width classes [47] was also calculated via a specific formula in Microsoft Excel (2019).


$$\begin{array}{c} H^{\prime }\;=\;-\sum_{i=\text{1}}^{n}p_{i}\text{ ln }p_{i} \end{array}$$

Where, $p_{i}$ is the proportion of individuals in the $i^{th}$class, and n is the total number of classes. The H′ values describe the distribution pattern of trait expression, reflecting both evenness and dominance across classes. Diversity indices were interpreted as low (0.1–0.4), intermediate (0.4–0.6), high (0.6–0.8), and very high (>0.8) [48]. Principal component analysis (PCA) was performed via the Factoextra [49], FactoMineR [50] and gridextra [51] packages. The optimum number of clusters was selected via the Factoextra package [49] and the Nbclust package [52]. The hierarchical cluster dendrogram was constructed using the UPGMA method with Euclidean distance, estimated from dendextend [53] and circilize [54] packages, from the 17 quantitative traits across the 58 bean landraces.

## 3. Molecular Characterization

### 3.1. Genomic DNA extraction

Approximately 0.2 g of leaf samples from 2–3-week-old plants of each accession were collected, and genomic DNA was extracted with following the CTAB protocol [55], with minor modifications, including the addition of polyvinylpyrrolidone (PVP) to remove polyphenols, increased NaCl concentration to improve polysaccharide removal, and an extended chloroform:isoamyl alcohol purification step [56]. The DNA concentration and quality were checked using the A260/A280 ratio with a Quawell UV-Vis spectrophotometer Q5000S. The extracted DNA was stored at −20°C for PCR amplification.

### 3.2. PCR amplification and SSR genotyping

In total, 15 SSR primer pairs (S4 Table) were used in this study to assess the genetic diversity among the bean accessions. The DNA sample was then diluted to a final concentration of 50 ng/μl for PCR. The primers were also diluted to a final concentration of 250 ng/μl. PCR analysis was carried out in a total reaction volume of 15 µL, including 7.5 µL of master mix 2X Green GoTaq® (Promega 25 ml) which contains Taq DNA polymerase, dNTPs, MgCl_2_, reaction buffer, and loading dyes, 1.5 µL of forward and 1.5 µL of reverse primer (Promega 25 ml), 2.5 µL of nuclease-free water (Promega 25 ml), and 2 µL of DNA using the MiniAmp thermal cycler. The specific denaturation, annealing, and extension temperatures are given in (S4 Table). The PCR products were separated on a 1.5% agarose gel (BIONEER 500 gm) using 1x TAE buffer (GENAXXON bioscience 500 ml). PCR products (15 µL) were loaded onto the gel along with 1 kb and 100 bp DNA ladders (GENAXXON Bioscience) for base pair size estimation. The gel was stained with ethidium bromide and subjected to electrophoresis. The DNA bands were visualized under UV light using a gel documentation system (PowerPro, Cleaver Scientific).

### 3.3. Molecular data analysis

The SSR bands were scored on the basis of the presence and absence of the bands, the 1 and 0 scoring and band size were estimated from ImageLab software. The frequency of alleles, observed heterozygosity, expected heterozygosity, polymorphic information content, and fixation index were calculated via Power Marker (3.0) software [57]. The PIC value was calculated as PIC= 1−∑pi2, where p represents the frequency of an allele [38]. The percentage of polymorphisms, marker index, resolving power, and effective multiplex ratio were calculated via specific formulae in Microsoft Excel. The percentage of polymorphism was calculated by POP =Number of polymorphic bands/Total number of bands. The resolving power was calculated via the formula RP = Ib= 1−(2×0.5−P∣), where P represents the proportion of individuals containing bands and Ib = Bands informativeness for each band [58]. The effective multiplex value was calculated via the formula EMR= Number of polymorphic bands×numbers of polymorphic bands/Total number of bands [38]. The marker index was calculated via the formula MI=PIC×EMR, where PIC = polymorphic information content and EMR = effective multiplex [38]. The molecular hierarchical cluster dendrogram was prepared via the average method and Nei’s genetic distance (1973) via NTsyspc software [59]. Principal coordinate analysis (PCoA) and analysis of molecular variance (AMOVA) were performed via Gene Alex (6.0) software [60]. To analyze the PCoA and AMOVA, bean landrace accessions were grouped into three populations based on collection altitude, representing agro-ecological regions of Nepal: Population 1 (Terai, < 500 m) with 6 accessions, Population 2 (Mid-hills, 500–1500 m) with 18 accessions, and Population 3 (High hills, > 1500 m) with 34 accessions. The population structure analysis and expected heterozygosity and fixation index (F_st_) calculations for each subpopulation were performed via STRUCTURE software V.2.3.4 [61]. The optimum number of clusters was selected by implementing the structure selector [62] and Evanno’s ∆K method [63]. The analysis involved Markov Chain Monte Carlo (MCMC) simulations by setting the k value from 2 to 10 with 5 iterations, with burn-in periods of 10000 and 100,0000.

## 4. Results

### 4.1. Quantitative traits-based diversity assessment

Analysis of 23 quantitative traits of the bean germplasm revealed considerable variation across phenological, yield, and yield-related traits, as evidenced by their observed ranges, mean values, standard errors, coefficients of variation (CVs), and Shannon-Weaver diversity indices (H′) (Table 1). The coefficient of variance ranged from 10% to 68%, which indicates low to high variation among the quantitative traits. Very high variability (>25%) was found for six traits: total yield (68%), plant height (52%), number of pods per plant (46%), thousand-seed weight (42%), chlorophyll content at the vegetative stage (30%) and number of raceme/plant (28%). Shannon Weaver’s index ranged from 0.46 to 0.92, which represented a moderate to very high level of diversity. Very high diversity (>80%) was found for 12 quantitative traits: leaf breadth (0.92), leaf length (0.9), days to first fruiting (0.9), seed length (0.87), seed thickness (0.87), days to 50% flowering (0.86), days to 80% maturity (0.85), days to first flowering (0.84), days to 80% germination (0.83), stem diameter (0.83), days to 50% fruiting (0.82) and plant height (0.82).

**Table 1:** Descriptive statistics and Shannon Weaver diversity among 23 quantitative traits of bean landraces.

Quantitative variables	Min	Max	Mean±SE	STD	CV (%)	H’
Days to 80% germination	18	30	21.46 ± 0.32	2.41	11	0.83
Chlorophyll content at the vegetative stage (CCI)	11.74	41.5	21.54 ± 0.86	6.56	30	0.67
Leaf length (cm)	6.18	10.6	8.17 ± 0.13	1.02	13	0.90
Leaf breadth (cm)	3.27	6.89	5.10 ± 0.10	0.73	14	0.92
Days to first flowering	50	83	62.15 ± 1.27	9.69	16	0.84
Days to 50% flowering	52	87	69.51 ± 1.49	11.35	16	0.86
Days to first fruiting	53	85	66.34 ± 1.21	9.24	14	0.90
Days to 50% fruiting	56	95	75.48 ± 1.60	12.15	16	0.82
Number of flower bud/raceme	2	5	3.72 ± 0.08	0.62	17	0.66
Number of raceme/plants	5	14	8.20 ± 0.30	2.32	28	0.75
Pod length (cm)	5.95	15.13	9.58 ± 0.28	2.14	22	0.64
Pod width (mm)	7.54	16.26	9.97 ± 0.19	1.48	15	0.80
Plant height (cm)	20.65	178.60	91.04 ± 6.26	47.66	52	0.82
Stem diameter (mm)	4.00	8.01	6.23 ± 0.10	0.77	12	0.83
Number of nodes/plant	4	8	5.63 ± 0.15	1.15	20	0.62
Number of pods/plant	4	27	10.56 ± 0.64	4.90	46	0.76
Number of seed/pod	3	9	4.86 ± 0.13	1.00	21	0.46
Days to 80% maturity	85	123	102.70 ± 1.35	10.31	10	0.85
Seed length (mm)	9.90	20.35	12.97 ± 0.24	1.79	14	0.87
Seed breadth (mm)	5.72	13.60	7.28 ± 0.15	1.11	15	0.80
Seed thickness (mm)	3.95	9.06	5.48 ± 0.11	0.84	15	0.87
Thousand seed weight (g)	131.25	1236.25	344.42 ± 18.86	143.65	42	0.75
Total yield (t/ha)	0.02	2.01	0.85 ± 0.08	0.58	68	0.74

Min = minimum, Max = maximum, STD = standard deviation, CV = coefficient of variation, H’ = Shannon–Weaver diversity index.

Early phenological traits exhibited substantial variability. Days to 80% germination ranged from 18 to 30 days (21.46 ± 0.32; H′ = 0.83), days to first flowering from 50 to 83 days (62.15 ± 1.27; H′ = 0.84), and days to 50% flowering from 52 to 87 days (69.51 ± 1.49; H′ = 0.86), all showing very high diversity with accessions broadly spread across phenotypic classes without concentration in any single range. The reproductive phenological traits showed pronounced heterogeneity. Days to first fruiting (53–85 days; 66.34 ± 1.21) recorded the highest phenological diversity (H′ = 0.90), suggesting near-uniform distribution across SD-based classes. Days to 50% fruiting (56–95 days; 75.48 ± 1.60; H′ = 0.82) and days to 80% maturity (85–123 days; 102.70 ± 1.35; H′ = 0.85) also showed very high diversity, reflecting broad spread without dominance of any particular maturity class.

Vegetative growth traits similarly showed high to very high diversity. Plant height ranged widely from 20.65 to 178.60 cm (91.04 ± 6.26; H′ = 0.82), indicating an even spread across height classes from short determinate bush types to tall climbing accessions with no single height class dominating. Leaf length ranged from 6.18 to 10.60 cm (8.17 ± 0.13; H′ = 0.90) and leaf breadth from 3.27 to 6.89 cm (5.10 ± 0.10; H′ = 0.92), both showing the highest vegetative diversity, reflecting near-uniform distribution of leaf dimensions across SD-based classes. Among yield-contributing traits, pod length ranged from 5.95 to 15.13 cm (9.58 ± 0.28; H′ = 0.64) and number of pods per plant from 4 to 27 (10.56 ± 0.64; H′ = 0.76), showing moderate to high diversity with some concentration towards intermediate classes. Number of seeds per pod (3–9; 4.86 ± 0.13) showed the lowest diversity (H′ = 0.46), indicating a strong concentration of accessions near the mean with limited representation in extreme classes. Thousand-seed weight varied widely from 131.25 to 1236.25 g (344.42 ± 18.86; H′ = 0.75) and total yield from 0.02 to 2.01 t ha ⁻ ¹ (0.85 ± 0.08; H′ = 0.74), both showing high diversity with accessions broadly distributed across classes without strong dominance by any single class.

### 4.2. Qualitative traits-based diversity assessment

The frequency, percentage, and Shannon Weaver diversity index of 24 qualitative traits of 58 bean landraces are presented in Table 2. The diversity index (H’) ranged from 0.085 to 0.99, which indicates that low to very high diversity is present among the 24 qualitative traits of the bean landraces. The highest diversity was found for leaf shape (0.99), and the lowest diversity was found for seed coat color (0.085). Only three traits, leaf shape (0.99), bracteole shape (0.99), and hypocotyl pigmentation (0.94), presented very high diversity (>80%).

**Table 2:** Frequency, Percentage and Shannon Weaver diversity of 24 qualitative traits.

Variables	Descriptor states	Frequency (n)	Percentage (%)	H’
Hypocotyl pigmentation	1-Purple	12	20.69	0.94
	2-Green	46	79.31	
Emerging cotyledon color	3-Green	32	55.17	0.39
	4-Very pale green	25	43.1	
	6-other	1	1.72	
Leaf color of chlorophyll	3-Pale green	11	18.97	0.63
	5-Medium green	31	53.45	
	7-Dark green	16	27.59	
Leaf hairiness	3-low	7	12.07	0.45
	5-Medium	43	74.14	
	7-High	8	13.79	
Leaf shape	1-Triangular	31	53.45	0.99
	2-Quadrangular	27	46.55	
Flower color	1-White	21	36.21	0.32
	3-Lilac	25	43.1	
	7-Dark lilac with purplish spots	3	5.17	
	9-Purple	9	15.52	
Plant type	1-Determinate bush	26	44.83	0.24
	2-Indeterminate bush with erect branches	5	8.62	
	3-Indeterminate bush with prostate branches	2	3.45	
	4-indeterminate with semi-climbing main stem and branches	14	24.14	
	5-indeterminate with moderate climbing ability and pods are distributed evenly up to the plant	3	5.17	
	6-indeterminate with aggressive climbing and pods are mainly on the upper nodes of the plant.	8	13.79	
Bracteole size	3-Small	21	36.21	0.58
	5-Medium	28	48.28	
	7-Large	9	15.52	
Bracteole shape	3-Lanceolate	28	48.28	0.99
	7-Ovate	30	51.72	
Flower bud size	3-Small	12	20.69	0.66
	5-Medium	26	44.83	
	7-Large	20	34.48	
Pod curvature	3-Straight	12	20.69	0.65
	5-Slightly curved	28	48.28	
	7-Curved	18	31.03	
Pod beak orientation	3-Upward	20	34.48	0.61
	5-Straight	27	46.55	
	7-Downward	11	18.97	
Pod beak position	1-Marginal	32	55.17	0.39
	2-Non-marginal	25	43.1	
	3-Others	1	1.72	
Wing/pod opening	3-Parallel closed wings	20	34.48	0.58
	5-Wings moderately diverging	29	50	
	7-Wings widely diverging	9	15.52	
Pod color	1-Dark purple	1	1.72	0.28
	3-Purple strips on green	2	3.45	
	4-Carmine strips on green	7	12.07	
	6-Dark pink	1	1.72	
	7-Normal green	18	31.03	
	8-Shiny green	25	43.1	
	9-Dull green or silver grey	4	6.9	
Seed coat pattern	0-Absent	31	53.45	0.11
	1-Constant mottled	1	1.72	
	2-Stripped	15	25.86	
	3-Rhomboid spotted	3	5.17	
	4-Speckled	4	6.9	
	5-Circular mottling	3	5.17	
	7- Broad striped	1	1.72	
Seed coat color	1-Black	10	17.24	0.085
	2-Brown pale to dark	14	24.14	
	3-Maroon	8	13.79	
	5-Yellow to greenish yellow	3	5.17	
	6-Pale cream to buff	15	25.86	
	7-Pure white	2	3.45	
	8-Whitish	3	5.17	
	9-White purple tinged	1	1.72	
	12-Red	2	3.45	
Seed shape	1-Round	4	6.9	0.24
	2-Oval	12	20.69	
	3-Cuboid	33	56.9	
	4-Kidney shaped	3	5.17	
	5-Truncate fastigiate	6	10.34	
Brilliance of the seed	3-Matt	2	3.45	0.64
	5-Medium	27	46.55	
	7-Shiny	29	50	
Number of seed color	1-Black	23	39.66	0.14
	2-Brown pale to dark	19	32.76	
	3-Maroon	12	20.69	
	4-Gray, brownish to greenish	2	3.45	
	5-Yellow to greenish yellow	2	3.45	
Primary/main seed color	1-Black	9	15.52	0.085
	2-Brown, pale to dark	15	25.52	
	3-Maroon	7	12.07	
	5-Yellow to greenish yellow	3	5.17	
	6-Pale cream to buff	16	27.59	
	7-Pure white	2	3.45	
	8-Whitish	3	5.17	
	9-White, purple tinged	1	1.72	
	12-Red	2	3.45	
Secondary seed color	1-Black	2	5.56	0.090
	2-Brown, pale to dark	5	13.89	
	3-Maroon	11	30.56	
	5-Yellow to greenish yellow	4	11.11	
	6-pale cream to buff	8	22.22	
	12-Red	3	8.33	
	13-Pink	1	2.78	
	14-Purple	2	5.56	
Seed veining	+-Absent	48	82.76	0.66
	0-Present	10	17.24	
Seed hilum color	8-Whitish	52	89.66	0.48
	12-Red	6	10.34	

Note: H′ values are rounded to two decimal places; some values may appear identical due to rounding.

Leaf shape showed the highest diversity (H′ = 0.99) due to the nearly equal distribution of triangular (53.45%) and quadrangular (46.55%) forms among the 58 bean landraces. Similarly, Bracteole shape (H′ = 0.99) also had almost equal proportions of lanceolate (48.28%) and ovate (51.72%) forms, with no dominant state. Hypocotyl pigmentation (H′ = 0.94) was largely green (79.31%) over purple (20.69%), yet showed very high diversity as both states were well represented across accessions. Leaf chlorophyll color showed moderate diversity (H′ = 0.63), with medium green as the predominant class (53.45%), followed by dark green (27.59%) and pale green (18.97%), showing moderate concentration towards one class. Leaf hairiness showed intermediate diversity (H′ = 0.45), with the medium class strongly dominant (74.14%) and limited representation in low and high classes. Flower color showed low diversity (H′ = 0.32), with lilac (43.10%) and white (36.21%) together comprising nearly 80% of accessions, leaving purple and dark lilac forms poorly represented. Plant growth habit showed limited diversity (H′ = 0.24), with determinate bush type accounting for 44.83% of accessions and the remaining five growth types collectively representing the rest. Pod color showed low diversity (H′ = 0.28), with shiny green (43.10%) and normal green (31.03%) as the dominant classes and pigmented types being rare. Seed-related traits generally showed low to very low diversity. Seed coat pattern (H′ = 0.11) was strongly dominated by the absent class (53.45%), with the remaining pattern types collectively accounting for less than half of accessions. Seed coat color (H′ = 0.085) and primary seed color (H′ = 0.085) were the least diverse traits, with pale cream to buff and brown as the dominant classes across nine possible color states. The number of seed colors (H′ = 0.14) was similarly concentrated, with black (39.66%) and brown (32.76%) together accounting for over 70% of accessions. Seed shape showed low diversity (H′ = 0.24), with cuboid as the dominant form (56.90%), oval (20.69%), and truncate fastigiate (10.34%) having limited presence. Seed brilliance showed moderate diversity (H′ = 0.64), with medium (46.55%) and shiny (50%) classes together covering nearly all accessions, showing moderate evenness between two states.

### 4.3. Principal component analysis

The results of the principal component analysis (PCA) of twenty-three quantitative characteristics of the bean landraces are presented in Table 3. Dimension-1 contains the most variance (28.49%) in the whole dataset, followed by dimension-2 (17.77%), with a cumulative variance of 46.27%. The first five principal components with eigenvalues>1 (PC1-C5) were retained for further analysis and accounted for 76.53% of the total variation, with PC-5 explaining more than ~70% of the variation (PC1: 28.49%, PC2: 17.17%, PC3: 10.39%, PC4: 8.28%, PC5: 5.93%). The first principal component (PC-1) is highly shaped by phenology, vegetative growth, and yield performance, including days to 50% fruiting (−0.360), days to 50% flowering (−0.358), days to first flowering (−0.329), days to 80% maturity (−0.283), plant height (−0.273), number of racemes per plant (−0.265), total yield (0.240) and number of nodes per plant (−0.273). The second principal component (PC-2) is highly dominated by seed characteristics and physiological attributes, including thousand-seed weight (0.415), seed breadth (0.405), seed thickness (0.352), number of flower bud per raceme (−0.257), stem diameter (−0.256) and chlorophyll content at the vegetative stage (0.221) were used.

**Table 3:** PCA table of 23 quantitative traits of common bean.

Dimensions	PC1	PC2	PC3	PC4	PC5
Eigenvalue	6.55	4.09	2.39	1.9	1.36
Variance (%)	28.49	17.77	10.39	8.28	5.93
Cumulative variance (%)	28.49	46.27	56.65	64.93	70.86
**Variables**	**Coefficient vectors**				
Days to 80% germination	0.016	0.182	**−0.298**	**−0.216**	**−0.234**
Chlorophyll content at vegetative stage	−0.035	**0.221**	0.151	**−0.209**	**−0.569**
Leaf length (cm)	0.119	−0.191	**−0.393**	**0.253**	**−0.257**
Leaf breadth (cm)	−0.025	−0.131	**−0.511**	0.188	**−0.264**
Days to first flowering	**−0.329**	0.131	−0.04	−0.01	**−0.209**
Days to 50% flowering	**−0.358**	0.111	−0.028	−0.009	−0.169
Dyas to first fruiting	−0.34	0.157	−0.069	0.072	−0.066
Days to 50% fruiting	**−0.36**	0.135	−0.025	0.009	−0.147
Number of flower bud/raceme	0	**−0.257**	0.127	**0.268**	−0.013
Number of raceme/plant	**−0.265**	−0.028	0.017	−0.027	0.077
Pod length (cm)	−0.205	−0.016	**−0.411**	−0.054	**0.321**
Pod width (mm)	−0.093	0.125	**0.291**	**0.352**	0.036
Plant height (cm)	**−0.273**	0.014	−0.037	0.131	**0.22**
Stem diameter (mm)	−0.003	**−0.256**	0.072	**0.338**	**−0.333**
Number of nodes/plant	**−0.237**	−0.031	0.001	0.129	−0.01
Number of pods/plant	−0.114	−0.179	0.12	**0.383**	−0.08
Number of seed/pod	−0.103	−0.174	**−0.321**	−0.057	**0.219**
Days to 80% maturity	**−0.283**	0.034	0.038	**0.213**	**0.215**
Seed length (mm)	0.188	0.316	**−0.221**	0.109	0.062
Seed breadth (mm)	0.091	**0.405**	−0.056	**0.288**	0.032
Seed thickness (mm)	0.174	**0.352**	−0.016	**0.285**	0.093
Thousand seed weight (g)	0.124	**0.415**	−0.096	**0.211**	0.038
Total yield (t/ha)	**0.24**	−0.137	−0.091	0.204	−0.05

**Fig 3:**
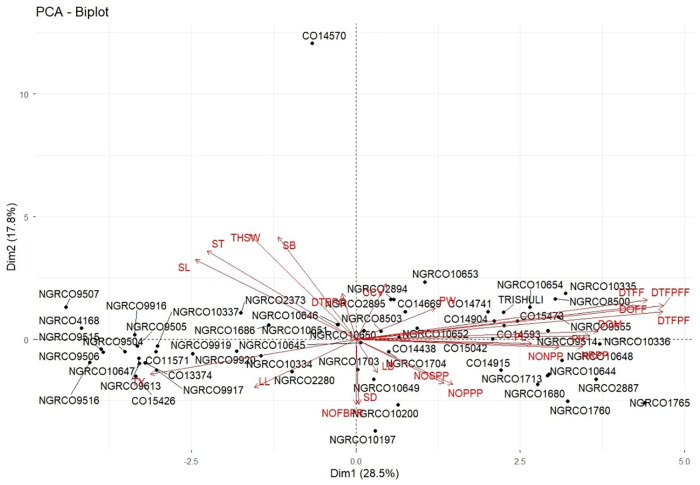
PCA biplot of common bean landraces.

The PCA biplot (Fig 3) revealed considerable phenotypic diversity among the bean landraces. In PC1, accessions such as NGRCO10335, NGRCO10644, NGRCO1765, and CO14741 exhibited strong associations with reproductive and yield-related traits, including days to 50% flowering, days to first fruiting, days to 50% fruiting, number of pods per plant, and number of seeds per pod. The separation observed along PC2 was limited to a few accessions, notably CO14570, and is largely explained by high loadings of seed-related traits, including seed length, seed breadth, seed thickness, and thousand-seed weight.

The PC-3 mainly captured differences in leaf and pod traits along with early growth characteristics, showing that variation in how the plants develop at early stages and build their vegetative structure contributed to the overall diversity among the accessions. PC-4 was more related to yield and plant structure traits, suggesting that differences in reproductive capacity and overall plant architecture were important in distinguishing the accessions. PC-5 reflected variation in physiological vigor and plant growth behavior, indicating that traits such as chlorophyll content, growth pattern, and general plant performance also played a role in shaping the observed diversity. Principal component analysis (PCA) revealed that days to 50% fruiting, days to 50% flowering, thousand seed weight, days to first fruiting, seed breadth, days to first flowering, leaf breadth, leaf length, seed thickness, number of pods per plant, pod length, seed length, chlorophyll content at the vegetative stage and days to 80% maturity were the traits that contributed the most to the overall observed variation.

### 4.4. Hierarchical clustering

**Fig 4:**
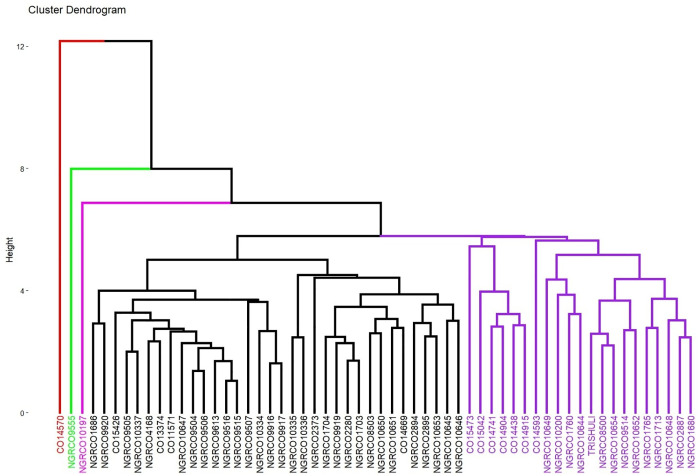
Hierarchical cluster dendrogram of bean landraces using 17 quantitative traits.

The hierarchical clustering dendrogram classified the accessions into the 5 clusters (Fig 4). Clusters I, II, III, IV, and V are represented by red, green, cyan, black, and purple colors, respectively (Fig 3). Clusters I, II, III, IV, and V consisted of 21, 34, 1, 1, and 1 accession, respectively. Cluster II contained the largest number of accessions, 34, whereas clusters III, IV, and V contained the lowest number of accessions (Table 4).

**Table 4:** Clustering means of the 5 clusters for 17 quantitative traits.

Cluster	I	II	III	IV	V
Cluster number	21	34	1	1	1
Days to 80% germination	21.10	22.10	18.00	21.00	26.00
Chlorophyll content at vegetative stage	20.20	22.60	25.90	19.60	41.50
Leaf length (cm)	8.30	8.00	6.92	10.60	6.18
Leaf breadth (cm)	5.03	5.27	3.27	6.89	4.31
Days to 50% flowering	62.40	79.50	87.00	70.00	86.00
Days to 50% fruiting	68.60	85.10	87.00	75.00	94.00
Pod length (cm)	8.68	11.30	5.95	9.40	9.08
Pod width (mm)	10.10	9.51	16.30	9.88	10.20
Plant height (cm)	78.30	114.00	101.00	65.50	59.00
Number of pods per plant	9.26	11.40	24.00	27.00	7.00
Number of seed per pod	4.56	5.38	3.00	7.00	4.00
Days to 80% maturity	99.50	108.00	118.00	98.00	100.00
Seed length (mm)	13.40	12.10	11.90	11.60	20.40
Seed width (mm)	7.45	6.64	8.29	7.91	13.60
Seed thickness (mm)	5.84	4.77	5.76	4.69	9.06
Thousand seed weight (g)	371.00	264.00	297.00	293.00	1236.00
Total yield (t/ha)	1.00	0.59	0.89	2.00	0.63

Cluster II, the largest cluster, was characterized by delayed flowering (79.50 days) and maturity (108 days), the tallest plants (114 cm), and a relatively high chlorophyll content (22.60) but a relatively low yield (0.59 t/ha). Cluster III, represented by a single landrace, exhibited early germination (18 days) but very late maturity (118 days), high pod width (16.30 mm), and a large number of pods per plant (24), resulting in a moderate yield (0.89 t/ha). Cluster IV emerged as the most productive cluster, with the highest yield (2 t/ha), maximum number of pods per plant (27) and number of seeds per pod (7), early maturity (98 days), and relatively large leaf dimensions (leaf length of 10.60 and leaf width of 6.89), with a length of 10.60 cm and breadth of 6.89 cm. In contrast, Cluster V was distinct in terms of exceptionally high chlorophyll content (41.50) and the highest seed length (20.40 mm), seed width (13.60 mm), seed thickness (9.06 mm), and thousand seed weight (1236 g) but delayed germination (26 days).

The inter-cluster centroid distance analysis approach helps in identifying genetically distinct groups for effective parent selection in hybridization programs and utilization in breeding programs (Table 5). The greatest genetic distance was observed between Cluster II and Cluster V (974.09), followed by the distances between Cluster IV and Cluster V (944.27), Cluster III and Cluster V (940.95), and between Cluster I and Cluster V (866.76), demonstrating that Cluster V is genetically most distinct from the other clusters. In contrast, the lowest inter-cluster distance occurred between Cluster II and Cluster III (41.01), followed by Cluster III and Cluster IV (47.74), indicating close genetic similarity among these clusters. Moderate divergence was recorded among Cluster I with Clusters II, III, and IV.

**Table 5:** Cluster centroid distance among 5 cluster.

	Cluster I	Cluster II	Cluster III	Cluster IV	Cluster V
Cluster I	0.00	115.35	87.11	81.60	866.76
Cluster II	115.35	0.00	41.01	61.13	974.09
Cluster III	87.11	41.01	0.00	47.74	940.95
Cluster IV	81.60	61.13	47.74	0.00	944.27
Cluster V	866.76	974.09	940.95	944.27	0.00

## 5. Molecular marker-based diversity assessment

### 5.1. Genetic diversity statistics

The genetic diversity statistics are presented in Table 6. The genetic diversity of 58 common bean landraces was assessed via 15 SSR markers. Among the 15 SSR primers, three were monomorphic, whereas the remaining 12 markers were polymorphic. The monomorphic markers were excluded, and only polymorphic markers were selected for further analysis.

**Table 6:** Genetic diversity statistics of 12 genetic traits among 58 bean landraces.

Primers	Base pairs	Total bands		Total alleles	Polymorphic alleles			Allele frequency		Observed heterozygosity (H_o_)	
PV-cct001	100-150	61		3	3			0.776		0.372	
BM143	100-180	61		3	3			0.543		0.511	
BM170	200-500	69		3	3			0.905		0.173	
BM184	100-160	55		3	3			0.759		0.386	
BM187	180-500	59		4	4			0.569		0.547	
PV-BR35	100-200	55		3	3			0.534		0.540	
PV-BR185	100-150	55		3	3			0.603		0.514	
SSR-IAC10	280-800	86		5	5			0.655		0.505	
SSR-IAC62	100-150	56		3	3			0.603		0.504	
BMd-12	100-150	61		2	3			0.603		0.479	
BM152	100-150	58		3	3			0.690		0.438	
BM210	100-200	55		3	3			0.776		0.361	
Sum		731		38	38						
Mean		60.92		3.16	3.16			0.668		0.444	
						
Expected Heterozygosity (H_e_)			PIC			f	RP		EMR		MI
0.172			0.34			0.542	1.552		2		0.68
0.052			0.40			0.901	1.086		2		0.8
0.190			0.16			−0.087	1.81		2		0.32
0.000			0.34			1.000	1.518		2		0.68
0.086			0.46			0.845	1.138		2		0.92
0.000			0.44			1.000	1.068		2		0.88
0.000			0.43			1.000	1.206		2		0.86
0.190			0.45			0.630	1.31		2		0.9
0.000			0.41			1.000	1.206		2		0.82
0.034			0.36			0.929	1.206		2		0.72
0.000			0.36			1.000	1.38		2		0.72
0.000			0.32			1.000	1.552		2		0.64
							16.032		24		8.94
0.060			0.37			0.866	1.33		2		0.745

Note: PIC = Polymorphic information content, EMR = Effective multiplex ratio, MI = Marker index and RP = Resolving power, f = Fixation index

The size of the band ranged from 100–800 base pairs. The total number of bands was 731, with an average of 60.92 bands for each marker. The highest number of bands amplified by the BM170 primer was 69, the lowest was 55, produced by BM184, PV-BR35, PV-BR185 and BM210. The total number of alleles was 38, with an average of 3.16 alleles per locus. Similarly, the total number of polymorphic alleles was 38, with an average of 3.16. The allele frequency distribution spanned from 0.534–0.905, which is a medium-high frequency distribution. The allele frequency distribution generally ranges from 0–1.

The observed heterozygosity (H_o_) ranged from 0.173 (BM170) to 0.547 (BM187), with an overall mean of 0.444, indicates a moderate level of heterozygosity within the germplasm. This pattern suggests differential allelic expression across loci, consistent with the presence of both fixed and segregating alleles. The expected heterozygosity (H_e_) ranged from 0 to 0.19, with an average of 0.06, reflected the predominance of alleles with low frequency across the SSR loci. Certain loci, such as SSR-IAC10, exhibited relatively higher H_e_ (0.19), contributing disproportionately to the overall genetic diversity of the population. Polymorphic information content (PIC), an indicator of the discriminatory capacity of each SSR marker, ranged from 0.16 (BM170) to 0.46 (BM187), with a mean of 0.37. Markers with higher PIC values, including BM187 and SSR-IAC10, were particularly effective in revealing genetic variation among landraces. The resolving power (RP) of SSR markers, reflecting their ability to discriminate among accessions, varied between 0.542 and 1.0, with an average of 0.866. This indicates a moderate to high discriminative capacity across loci. The effective multiplex ratio (EMR) was consistently 2 for all markers, suggesting uniform amplification efficiency. The marker index (MI), a composite measure of marker informativeness integrating RP and EMR, ranged from 0.32 (BM170) to 0.92 (BM187), with an average of 0.745. Higher MI values correspond to more informative markers, indicating their suitability for genetic differentiation and diversity studies.

### 5.2. Phylogenetic tree

**Fig 5:**
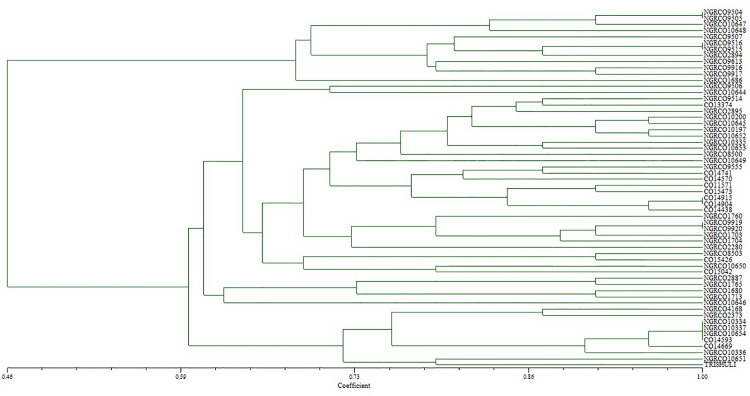
Molecular cluster dendrogram of 12 SSR markers among 58 common bean landraces.

The 58 bean landraces using the 12 SSR markers were classified into 3 major clusters (Fig 5). Cluster I consisted of 12 accessions, predominantly originating from the High hills (>1500 m) region, including accessions from Khotang, Dhankhuta, Panchthar, Sankhuwasabha, and Lamjung. The clustering indicates that accessions from the same district were grouped together, showing shared genetic backgrounds and potential local adaptation. Cluster II was the largest, containing 36 accessions, and included a mix of Mid-hills (500–1500 m) and High hills (>1500 m) accessions.

Among the 36 accessions, approximately 20 were from high-hill regions and 16 from mid-hill regions, highlighting the relatively broad genetic base within this cluster. Notably, accessions from the same district were generally clustered together but some districts exhibited distribution across subclusters. Cluster III comprised 10 accessions, with a dominance of High hills (>1500 m) origin. The cluster included accessions from Myagdi, Mustang, Panchthar, Dolakha, and the variety Trishuli. Overall, the dendrogram illustrates that geographic origin, particularly altitude, plays a significant role in the genetic structure of Nepalese bean landraces. Accessions from high-altitude districts tend to cluster together, whereas accessions from mid-hill and lowland showed more intermixing.

### 5.3. Principal coordinate analysis

**Fig 6:**
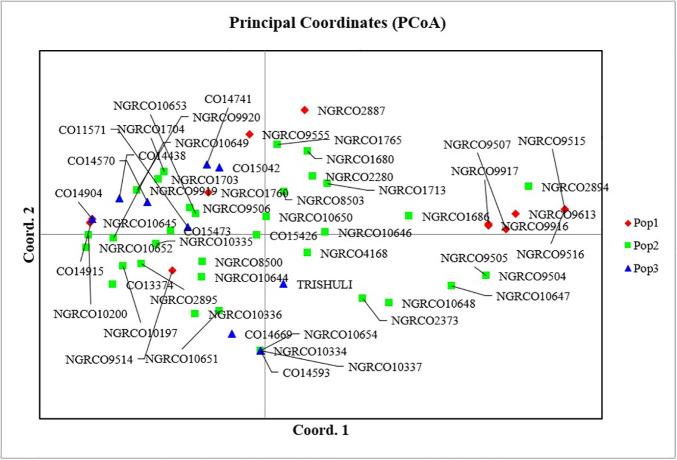
Molecular principal coordinate analysis showing three axes among three populations.

The PCoA identified three main axes that describe genetic differences among the bean accessions. Axis 1 explained 27.44% of the total variation, indicating that it represented the largest share of genetic diversity. Axis 2 adds another 15.83%, whereas Axis 3 accounts for 9.87%. Together, the first two axes capture approximately 43.27% of the total variation, offering a good two-dimensional view of the genetic patterns (Table 7; Fig 6). When the third axis is included, the cumulative explained variation increases to 53.14%, meaning that these axes together describe most of the genetic differences in the population.

**Table 7:** Principal coordinate analysis of 58 bean landraces for 3 axes.

Axis	1	2	3
Percentage (%)	27.44	15.83	9.87
Cumulative percentage (%)	27.44	43.27	53.14

These populations were used for principal coordinate analysis (PCoA) based on SSR markers. Unlike the clear separation observed in the cluster dendrogram, the PCoA plot reveals that accessions from different clusters are not strictly grouped together. Instead, there was noticeable overlap and intermixing among accessions belonging to Pop1, Pop2, and Pop3. While some accessions from the same cluster are positioned in proximity, others are dispersed and intermixed with accessions from different clusters.

### 5.4. Analysis of the molecular variance (AMOVA)

The analysis of molecular variance (AMOVA) was conducted via a Nei’s distance matrix of 58 accessions on the basis of the three molecular clusters. The AMOVA revealed that 93% of the total genetic variation resided within populations, whereas 7% was attributed to differences among populations (Table 8). This distribution indicates that individual accessions within each population harbor substantial genetic diversity. The AMOVA yielded a PhiPT value of 0.075 (P = 0.001), indicating moderate and statistically significant genetic differentiation among the populations. This result suggests that approximately 7% of the total genetic variation is attributable to differences between populations, whereas the remaining variation occurs within populations.

**Table 8:** Analysis of molecular variance (AMOVA) for 3 populations.

Source	df	SS	MS	Est. Var.	%	PhiPT	P value
Among Pops	2	43.923	21.962	0.781	7%	0.075	0.005
Within Pops	55	532.611	9.684	9.684	93%		
Total	57	576.534		10.465	100%		

Note: df = degree of freedom, SS = sum of squares, Est. Var = Estimated variance and % = Percentage of total variance

### 5.5. Population structure analysis

**Fig 7:**
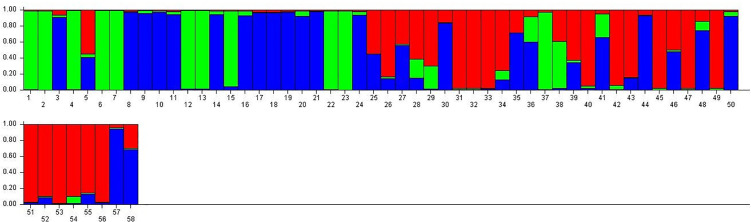
Population structure analysis of 12 SSR markers of 58 bean landraces.

The population structure of the 58 bean landraces based on 12 SSR markers revealed the presence of three major genetic clusters (K = 3), indicating the underlying genetic composition of the studied accessions. The STRUCTURE bar plot (Fig 7) illustrates the proportional membership coefficient (Q) of each accession in the inferred clusters, where each vertical bar represents an individual accession and the colors blue, green, and red represent the three inferred genetic groups. Based on the highest membership coefficient value, out of the 58 accessions, 26 accessions were assigned to subpopulation I, 11 accessions to subpopulation II, and 21 accessions to subpopulation III (Table 9). The STRUCTURE bar plot indicated that several accessions exhibited admixed ancestries, shown by multiple colors within a single bar, suggesting shared genetic components among clusters. However, some accessions showed predominant membership in a single cluster with high membership coefficients (Q values), indicating relatively distinct genetic backgrounds with limited admixture.

**Table 9:** Membership coefficient of bean accessions in three subpopulations.

	Subpopulation		
Accessions	**I**	**II**	**III**
NGRCO9504	0.011	0.981^*^	0.008
NGRCO9505	0.012	0.981^*^	0.007
NGRCO9506	0.061	0.031	0.908^*^
NGRCO9507	0.007	0.983^*^	0.01
NGRCO9514	0.543^*^	0.046	0.412
NGRCO9516	0.004	0.991^*^	0.005
NGRCO9515	0.004	0.991^*^	0.005
NGRCO1760	0.015	0.007	0.977^*^
NGRCO2887	0.006	0.032	0.961^*^
NGRCO9555	0.012	0.011	0.977^*^
NGRCO1765	0.018	0.038	0.944^*^
NGRCO2894	0.005	0.979^*^	0.016
NGRCO9613	0.007	0.975^*^	0.018
NGRCO1680	0.008	0.048	0.944^*^
NGRCO1686	0.011	0.945^*^	0.044
NGRCO2280	0.008	0.063	0.929^*^
NGRCO9919	0.028	0.006	0.967^*^
NGRCO9920	0.024	0.007	0.969^*^
NGRCO1703	0.019	0.006	0.976^*^
NGRCO8503	0.014	0.063	0.923^*^
NGRCO1704	0.012	0.005	0.983^*^
NGRCO9916	0.011	0.977^*^	0.011
NGRCO9917	0.008	0.985^*^	0.007
NGRCO1713	0.018	0.041	0.941^*^
NGRCO10200	0.544^*^	0.005	0.451
NGRCO10197	0.829^*^	0.026	0.145
NGRCO2895	0.428	0.011	0.561^*^
NGRCO4168	0.612^*^	0.238	0.15
NGRCO2373	0.698^*^	0.287	0.015
NGRCO10335	0.153^*^	0.007	0.84
NGRCO10334	0.975^*^	0.017	0.008
NGRCO10337	0.978^*^	0.014	0.008
NGRCO10336	0.972^*^	0.01	0.017
NGRCO10644	0.754^*^	0.113	0.132
NGRCO10645	0.279	0.005	0.716^*^
NGRCO10646	0.085	0.314	0.601^*^
NGRCO10647	0.022	0.966^*^	0.012
NGRCO10648	0.391	0.581^*^	0.028
NGRCO8500	0.627^*^	0.029	0.344
NGRCO10649	0.948^*^	0.029	0.023
NGRCO10650	0.046	0.295	0.659^*^
NGRCO10651	0.942^*^	0.049	0.008
NGRCO10652	0.839^*^	0.005	0.157
NGRCO10653	0.059	0.01	0.931^*^
NGRCO10654	0.977^*^	0.016	0.007
CO11571	0.498^*^	0.019	0.483
CO14669	0.977^*^	0.013	0.01
CO15426	0.141	0.113	0.746^*^
CO14593	0.975^*^	0.018	0.008
CO15042	0.016	0.056	0.928^*^
CO14915	0.97^*^	0.005	0.025
CO15473	0.894^*^	0.02	0.086
CO13374	0.982^*^	0.005	0.013
TRISHULI	0.894^*^	0.086	0.02
CO14438	0.855^*^	0.012	0.133
CO14904	0.969^*^	0.005	0.027
CO14741	0.041	0.014	0.945^*^
CO14570	0.298	0.01	0.692^*^

Genetic diversity parameters estimated for the three inferred subpopulations indicated moderate variation in expected heterozygosity (H_e_) among the clusters (Table 10). The expected heterozygosity ranged from 0.272 to 0.374, with a mean value of 0.312, and showed a moderate level of genetic diversity within the inferred populations. Among the clusters, subpopulation 3 exhibited the highest genetic diversity (H_e_ = 0.374), whereas subpopulation 1 showed the lowest diversity (H_e_ = 0.272). F_st_ serves as a measure of genetic divergence between different subgroups within a population. The fixation index (F_st_) ranged from 0.224 to 0.505, with an average value of 0.406. The highest genetic differentiation was observed in subpopulation 2 (F_st_ = 0.505), followed by subpopulation 1 (F_st_ = 0.488), indicating strong genetic differentiation from the overall population. In contrast, subpopulation 3 exhibited comparatively lower differentiation (F_st_ = 0.224), suggesting relatively higher genetic exchange or shared ancestry with other groups. These results indicate that while some level of admixture exists among the bean landraces, the identified clusters still maintain considerable genetic differentiation.

**Table 10:** Expected heterozygosity and F_st_ among the 3 subpopulations.

Subpopulation (K)	Expected heterozygosity (H_0_)	F_st_
1	0.272	0.488
2	0.289	0.505
3	0.374	0.224
Mean	0.312	0.406

## 6. Discussion

Landraces are vital for maintaining agrobiodiversity but are increasingly threatened by genetic erosion due to habitat loss, replacement by uniform elite varieties, urbanization, deforestation, overexploitation, and invasive alien species, leading to a reduction in overall genetic diversity [64]. Since the early 20^th^ century, approximately 75% of plant genetic diversity has been lost globally, largely due to farmers replacing diverse local varieties and traditional landraces with genetically uniform, high-yielding cultivars [65]. The rapid expansion of industrial and green revolution in agriculture over the last 100 years has resulted in the loss of more than 90% of crop varieties from farmers’ fields [65]. In Nepal, an average of about 40% of agricultural genetic resources (AGRs) have been lost nationwide, while in certain regions farmers have reported the complete disappearance (up to 100%) of native AGRs [66]. Therefore, it is essential to prioritize the morphological and molecular characterization of landraces, along with conservation-oriented breeding strategies using morphological and molecular markers and *ex situ* preservation through gene banks.

The common bean landraces exhibited considerable phenotypic and genotypic variation. The quantitative traits showed the moderate to very high level of diversity, whereas the qualitative traits showed a low to very high diversity [67]. A low H′ value indicates a highly uneven distribution of frequency classes for a given trait and reflects limited genetic diversity within the evaluated germplasm. Similarly, [68] and [69] reported H’ diversity indices for quantitative traits ranging from 0.47–0.73 and 0.36–0.97, respectively, and for qualitative data ranging from 0.57–0.58 to 0.47–0.57, which aligns with our findings. The days to first flowering ranged from 50−83 days, and the days to first fruiting ranged from 53−85 days. According to [70], reported 35−70 days for the number of days to first flowering and 40−84 days for days to 50% flowering, and [32] reported 40−63 days for days to 50% flowering. Accessions from hilly regions generally require a longer duration to initiate flowering than those from warmer Terai plains do [4,32]. The accession NGRCO9507 from Tanahun flowered earliest (50 days), whereas NGRCO9555 from Rupandehi exhibited the latest flowering. The observed variation in flowering and fruiting times is likely influenced by environmental factors, particularly temperature, which regulate physiological and phenological development [71]. Early-flowering and early-fruiting accessions can be utilized in breeding programs aimed at developing short-duration and stress-escaping cultivars, whereas late-maturing accessions may serve as valuable parents for improving adaptation and yield stability in favorable agroecological environments. The accessions showed wide variation in maturity duration, reaching physiological maturity between 85 and 123 days. According to [71], reported that the number of days to 80% maturity ranged from 80−149 days. It is a key agronomic trait influencing farmers’ varietal preference, as early-maturing cultivars reduce exposure to biotic stresses and enable earlier market entry and crop intensification [72,73]. The accession NGRCO4168, a bush-type accession, matured earliest (85 days), whereas NGRCO9514, an indeterminate and moderately climbing accession, exhibited the latest maturity (123 days). This variation is likely associated with differences in growth habits and temperature. The indeterminate genotypes maintain prolonged vegetative growth that delays reproductive development [74], and with low-temperature stress in high-altitude environments, extending crop phenology [70]. The number of pods per plant varied from 4 to 27 and the number of seeds per pod ranged from 3 to 9. These findings are comparable to earlier reports by [32], who reported 9–32 pods per plant and 3–6 seeds per pod. This variation is likely due to differences in growth habit and environmental adaptation among the accessions. Indeterminate and climbing types generally accumulate more assimilates and more reproductive timing, resulting in higher pod and seed formation, whereas determinate types exhibit synchronized flowering and relatively lower reproductive output [74,75]. In addition, environmental factors such as temperature variation across agroecological zones affect flowering success and seed set, contributing to differences in seed number per pod [70,71]. The common bean accessions have substantial variation in total yield, ranging from 0.01 to 2.01 t ha^-1^, with accession NGRCO9516 resulting in the highest yield (2.01 t ha^-1^). The superior performance of this accession is likely associated with its determinate growth habit and relatively short maturity duration (87 days), which favors synchronized flowering and pod set. These phenological attributes may increase yield components, including pod and seed numbers per plant, by enabling partial escape from abiotic stresses commonly affecting the flowering and pod development stages [75]. Pod length exhibited substantial variation among the accessions, ranging from 5.95 to 15.13 mm. The maximum pod length (15.13 mm) was recorded for the check variety NGRCO8500, followed closely by accession NGRCO1765 (15.01 mm). Pod length is a key economically important trait, as it directly influences yield potential and plays a significant role in marketability and consumer preference [21,26].

Among the 58 landraces studied, the flower color was predominantly lilac, followed by white, purple, and dark lilac with purplish spots. Flower color in common bean is regulated primarily by the V locus, where the dominant V allele promotes anthocyanin production, resulting in purple/pink flowers, and the recessive v allele produces white flowers [76]. Seed coat color is controlled mainly by loci such as P, V, and C, which regulate anthocyanin and flavonoid biosynthesis, with silencing of the P gene during domestication contributing to white-seeded phenotypes [76,77]. The color of the seed coat is mostly black and brown, which is characteristic of the Mesoamerican gene pool, whereas maroon, red, pink, purple, pale cream, and yellow-green hues strongly represent Andean types with diverse pigmentation patterns [6,11,78]. The presence of dual pigmentation and lighter seed colors suggests a mixed gene pool composition. Colored seeds, particularly black and red seeds, are often associated with relatively high antioxidant contents due to the presence of anthocyanins, making them valuable for nutritional enhancement [79]. Conversely, white-seeded varieties are preferred in certain markets for their aesthetic appeal and faster cooking times [80]. Seed traits are the primary characteristics of beans that influence both their productivity and economic value [4]. Morphologically, 27% of bean accessions of Andean origin had more than 40 g of seed/100 g of seed weight, whereas the remaining 72.41% were of Mesoamerican origin and had less than 25 g of seed/100 g of seed weight. This diversity in seed color observed among the studied common bean accessions reflects potential differences between the Andean and Mesoamerican gene pools. Seed shape can also be classified into 5 major shapes, and is dominated by cuboid seed shape. The observed variation in the seed coat pattern, including mottled patterns, aligns with [81], who noted that complex patterns arise from interactions between multiple loci. Common bean seed characteristics such as color, size, and shape play key roles in consumer selection, as individual preferences vary from person to person [26]. All of these types of seed-related diversity, including color and shape, might be due to hybridization between the gene pools, intergene pool introgression, recombination, mass selection, seed sale and exchange, selection for cooking traits such as taste, and minimum selection by farmers [82–85]. Considerable variation in plant growth type was observed across the accessions, which were classified into six unique categories. This indeterminate growth habit type prevalence is most likely a result of both ecological adaptation and the cropping strategy used in the region [86]. According to [32], this indeterminate semi-climber growth habit is most suited for high altitudes in the Himalayan area, where temperatures are lower than those in the lowland regions of Nepal. Beans are typically planted as companion crops with maize and amaranth in hilly areas, where climbing varieties are more attractive, whereas in plains, beans are typically cultivated as sole crop-bush types. Those that exhibited determinate growth habit and a shorter maturity time produced more pods and seeds per plant because these accessions were able to avoid the adverse circumstances of high daily temperatures and low humidity during the blooming and pod formation phases [75].

Principal component analysis (PCA) is a multivariate statistical method for simplifying complex datasets by identifying key traits, uncovering their interrelationships, key roles, and the nature of traits, which is crucial for recognizing the diversity within breeding material, identifying traits that contribute to successful growth and eliminating low breeding value traits [87]. The first six components captured 70.86% of the total variation, with traits such as days to 50% fruiting, days to 50% flowering, thousand seed weight, days to first fruiting, seed breadth, days to first flowering, leaf breadth, leaf length, seed thickness, number of pods per plant, pod length, seed length, chlorophyll content at the vegetative stage and days to 80% maturity contributing the most to the observed variation. These results are in agreement with those of various other studies [86–88]. Our study’s six principal components are quite comparable to those reported by [89], who reported that the overall number of the six components accounts for 83% of the variation. These traits could be used for selection and improvement of bean landraces for bean breeding program. Breeders can use this information to select parent lines that combine complementary vegetative and reproductive traits. Clustering a large number of germplasm accessions into a homogeneous group facilitates the selection of diverse parents, enables precise comparisons among populations, and increases the likelihood of combining desirable genes for superior progeny development [90]. In the present study, the landraces were grouped into five distinct clusters. Cluster V, represented by accession NGRCO14570, was characterized by superior seed length, breadth, and thickness and high seed weight, reflecting traits typical of the Mesoamerican gene pool and offering the potential to improve the size of seeds preferred by farming communities. The cluster IV accession NGRC10197 presented high yield potential, early maturity, a determinate growth habit, and relatively high numbers of pods and seeds per plant, making it a promising parent for yield improvement. Cluster I accession NGRCO4168 exhibited earlier 50% flowering and fruiting compared to accessions in the other clusters, indicating its potential usefulness in breeding programs aimed at developing early-maturing cultivars. The large inter-cluster distances among Clusters I, IV, and V suggest substantial genetic divergence, supporting the strategic use of NGRC10197 and NGRCO4168 in hybridization to combine early maturity with high yield, followed by introgression of seed size traits from NGRCO14570. The lack of correspondence between geographical origin and morphological clustering indicates that phenotypic diversity is shaped more by genetic drift, selection pressure, environmental effects, and seed exchange than by collection site [91,92].

The SSR primers amplified a wide range of fragments, ranging from 100 to 800 base pairs. This type of variability is characteristic of SSR markers, where repeat motif expansions or contractions generate different allele sizes [93]. Larger ranges may indicate higher mutability, whereas narrower ranges suggest stable motifs with lower mutation rates [94]. Differences in repeat motifs (di- versus tri-nucleotide) and flanking sequence lengths contribute to the observed fragment range [83]. The observed band size facilitates accurate allele scoring, which is crucial for distinguishing closely related landraces [95]. The total number of amplified bands per primer ranged from 55 to 69, indicating great diversity. Higher band counts often correlate with robust PCR amplification, whereas lower counts may indicate null alleles or amplification bias [38]. Heterozygous individuals contribute two bands per locus, whereas homozygous individuals contribute one, so higher band counts are associated with greater heterozygosity [96]. The total number of alleles ranged from 3 to 5. Variation in allele counts arises from the use of different markers and sample sizes collected from various locations [24]. According to [29], they used 25 SSR primers and reported an average of 4.20 alleles per primer, which is slightly greater than our findings. Our findings are in line with those of [17], who used 27 SSR primers and likewise reported 2−12 alleles per locus. SSRs typically exhibit high allelic richness in cross-pollinated species; however, in predominantly selfing species such as common bean, allele numbers are constrained by the self-pollinating reproductive system, which promotes homozygosity and reduces allelic richness through inbreeding [97]. In outcrossing species such as maize, where SSRs often exhibit >5 alleles per locus due to relatively high recombination rates and gene flow [98]. The observed heterozygosity (H_o_) exceeded the expected heterozygosity (H_e_) across most loci, reflecting an excess of heterozygotes in the studied Nepalese bean landraces. Common bean is predominantly self-pollinated, which reduces H₀ via limited outcrossing and increases homozygosity [99]. The (H_o_ > H_e_) for a self-pollinating species may result from occasional outcrossing events, farmer-mediated seed exchange, mixed cropping systems, or admixture between distinct populations, which maintain heterozygosity despite the predominantly self-pollinating nature of common bean [100,101]. This pattern may also reflect potential genotyping artifacts, such as null alleles and allele dropout, rather than true biological heterozygosity. The PIC reflects marker informativeness, with values >0.5 considered highly discriminatory [102]. The low to medium mean PIC aligns with the limited allele number (3−5), as the PIC is influenced by both allele count and frequency distribution [103]. These findings are comparable with [104], who reported PIC values of 0.33–0.66 in bean germplasm, whereas populations with fewer alleles, such as those reported by germplasm [41], exhibited lower PIC values (0.17–0.41). Higher PIC values indicate greater utility for genetic mapping and diversity studies [102,105]. Low PIC values are typically found among closely related genotypes, and high values are typically found among more genetically diverse genotypes, such as cross-pollinated species [106].

The molecular cluster dendrogram classified the accessions into the 2 clusters. Similarly, [17] and [107] also reported the major molecular cluster of 2 when 27 and 23 SSR primers were used. Cluster-1, cluster-2 and cluster-3 contained 12, 36 and 10 accessions, respectively. The UPGMA dendrogram did not reveal distinct clustering of the common bean accessions according to their collection sites, indicating a lack of clear geographic structuring. This finding was further supported by the results of [17,29] and [71], who reported that there was no significant correlation between geographical origin and genetic similarity in their cluster analysis of bean landraces. This lack of clear grouping suggests that beans from different regions are genetically similar and that such genetic similarity is likely due to high gene flow. In contrast, SSR-based UPGMA analysis by [1] revealed geographic separation among bean genotypes from Ordu, likely due to traditional local seed production and limited external seed exchange.

Principal coordinate analysis (PCoA) is an ordination method that transforms a similarity or dissimilarity matrix into a low-dimensional plot, aiming to preserve the original distances between individuals [108,109]. It is reliable when the first axes explain ≥25% of the variation. Together, the first two axes capture approximately 43.27% of the total variation and shows intermixing of the accessions. Similar kinds of PCoA patterns were found in the SSR-based characterization of bean landraces by [29,110] and [111] who reported intermixing of the landraces among populations. There was no correlation between PCoA grouping and cluster analysis in this study. These results suggest low genetic differentiation among common bean accessions from different regions, likely due to extensive gene flow by farmer-to-farmer seed exchange across districts, which is a dominant practice in Nepal. This exchange system promotes the spread of genetic material across wide areas, leading to the homogenization of genetic diversity. The analysis of molecular variance (AMOVA) revealed greater variation within populations than between populations, which also verified the PCoA results. Similarly, [112] reported an intrapopulation variance of 83% and a variance of 17%, and [17] reported an intra-population variance of 66% and an interpopulation variance of 34%. The population structure analysis identified 3 clusters, which also showed a wide range of admixture and shared ancestry among the populations. The high F_st_ values suggest substantial genetic differentiation among the subpopulations, supporting the presence of well-structured genetic variation within the studied bean landraces. The observed population structure, together with moderate heterozygosity and noticeable interpopulation differentiation, suggests that the genetic diversity within the studied common bean accessions is influenced by both intrapopulation variation and limited gene flow between subpopulations. The genetic variation revealed through PCoA, AMOVA and population structure analysis can largely be attributed to a combination of a range of evolutionary and ecological mechanisms among plant varieties. These include both artificial and natural selection, gene flow between populations, and random processes such as genetic drift.

## 7. Conclusion

The majority of qualitative, quantitative, and molecular traits evaluated in Nepalese common bean landraces revealed substantial phenotypic and genotypic diversity. Agro-morphological assessment revealed that many landraces outperformed the 3 check accessions in terms of key reproductive and yield-related traits such as; earliness and yield components. Farmers in Nepal are generally reported to prefer early fruiting and maturity, climbing growth habits, high yields, and more seeds per pod. The identified early-fruiting and early-maturing bush-type landraces can serve as valuable parents in hybridization with high-yielding climbing types to combine earliness and productivity. SSR-based molecular characterization confirmed the morphological variability, revealing moderate to high genetic diversity, high heterozygosity and allelic diversity. Most variation occurred within populations, and weak geographic structuring with substantial admixture indicated extensive gene flow among landraces. While SSR markers can be used to assess diversity and population structure effectively, future studies using high-density SNP genotyping and GWAS could provide a finer resolution of trait–marker relationships, which will support marker-assisted selection and the development of improved, farmer-preferring bean varieties adapted to diverse agro-ecological regions of Nepal.

## Supporting information

S1 Table Information about the collection of 58 landraces from 21 districts of Nepal. (DOCX)

S2 Table List of 23 quantitative characters studied among the common bean landraces. (DOCX)

S3 Table List of 24 qualitative characters studied among the common bean landraces. (DOCX)

S4 Table Details of 15 SSR primers, sequences and annealing temperature used in the research. (DOCX)
