# Supplementary material for: Morphological and molecular characterization of Nepalese Common Bean (Phaseolus vulgaris L.) Landraces
Source: PLoS One. 2026 Jul 30;21(7):e0354479. doi: 10.1371/journal.pone.0354479 (PMC13423178; doi:10.1371/journal.pone.0354479)
Supplement: S1 Table — (DOCX) [file pone.0354479.s001.docx]

S1 Table. Information about the collection of 58 landraces from 24 districts of Nepal

| S. N | Accessions | Collected district | Lattitude | Longitude | Altitude |
| --- | --- | --- | --- | --- | --- |
| 1 | NGRCO 9504 | Khotang | 27.21567 | 86. 795202 | 1523m |
| 2 | NGRCO 9505 | Khotang | 27.22415 | 86. 78719 | 1668m |
| 3 | NGRCO 9506 | Khotang | 27.22415 | 86. 78719 | 1668m |
| 4 | NGRCO 9507 | Tanahun | 28.001 | 83.9131 | 879m |
| 5 | NGRCO 9514 | Dhankhuta | 27. 0095 | 87. 31353 | 1490m |
| 6 | NGRCO 9516 | Dhankhuta | 27. 0011 | 87. 308 | 1380m |
| 7 | NGRCO 9515 | Dhankhuta | 27. 00244 | 87. 30722 | 1431m |
| 8 | NGRCO 1760 | Kavrepalanchok | 27.31 | 85.38 | 800m |
| 9 | NGRCO 2887 | Sindhupalchok | 27.9300 | 85.5800 | 1100m |
| 10 | NGRCO 9555 | Bara | 26. 88 | 85. 16 | 32m |
| 11 | NGRCO 1765 | Sindhupalchowk | 27.57 | 85.41 | 1100m |
| 12 | NGRCO 2894 | Sankhuwasaaba | 27.41 | 86.44 | 2300m |
| 13 | NGRCO 9613 | Sunsari | 26. 7466 | 87. 153185 | 109m |
| 14 | NGRCO 1680 | Lamjung | 28.2199 | 84.4852 | 1402m |
| 15 | NGRCO 1686 | Lamjung | 28.2745 | 84.3790 | 1981m |
| 16 | NGRCO 2280 | Jajarkot | 29.2748 | 82.1840 | 1800m |
| 17 | NGRCO 9919 | Jajarkot | 28. 79561 | 82. 16664 | 1783m |
| 18 | NGRCO 9920 | Jajarkot | 28. 79561 | 82. 16664 | 1783m |
| 19 | NGRCO 1703 | Bajura | 29.4900 | 81.4600 | 2675m |
| 20 | NGRCO 1704 | Bajura | 29.4900 | 81.4600 | 2675m |
| 21 | NGRCO 9916 | Chitwan | 27. 61861 | 84. 40333 | 195m |
| 22 | NGRCO 9917 | Chitwan | 27. 61861 | 84. 40333 | 195m |
| 23 | NGRCO 1713 | Achham | 28.2789 | 83.8501 | 1648m |
| 24 | NGRCO 10200 | Jumla | 29. 2425 | 82. 10528 | 2277m |
| 25 | NGRCO 10197 | Jumla | 29. 2425 | 82. 10528 | 2277m |
| 26 | NGRCO 2895 | Humla | 29.6767 | 81.9137 | 1350m |
| 27 | NGRCO 4168 | Myagdi | 28.3717 | 83.6066 | 1100m |
| 28 | NGRCO 2284 | Mustang | 28.8201 | 83.8471 | 3450m |
| 29 | NGRCO 2373 | Mustang | 28.8201 | 83.8471 | 3450m |
| 30 | NGRCO 10335 | Kailali | 29. 597 | 80. 489 | 1314m |
| 31 | NGRCO 10334 | Kailali | 29. 597 | 80. 489 | 1314m |
| 32 | NGRCO 10337 | Baitadi | 29. 597248 | 80. 489687 | 1315m |
| 33 | NGRCO 10336 | Kailali | 29. 597 | 80. 489 | 1314m |
| 34 | NGRCO 10644 | Solukhumbu | 27.41 | 86.44 | 1893m |
| 35 | NGRCO 10645 | Morang | 26. 736657 | 87. 514838 | 271m |
| 36 | NGRCO 10646 | Dhankhuta | 27. 00841 | 86. 31093 | 1485m |
| 37 | NGRCO 10647 | Dhankhuta | 27. 00956 | 87. 31090 | 1530m |
| 38 | NGRCO 10648 | Panchthar | 26. 93777 | 86. 687678 | 1600m |
| 39 | NGRCO 10649 | Panchthar | 26. 93777 | 86. 687678 | 1600m |
| 40 | NGRCO 10650 | Panchthar | 26. 93777 | 86. 687678 | 1600m |
| 41 | NGRCO 10651 | Panchthar | 26. 93777 | 86. 687678 | 1600m |
| 42 | NGRCO 10652 | Panchthar | 26. 91667 | 87. 68333 | 1699m |
| 43 | NGRCO 10653 | Panchthar | 26. 86667 | 87. 66667 | 1623m |
| 44 | NGRCO 10654 | Panchthar | 26. 86667 | 87. 66667 | 1623m |
| 45 | CO 11571 | Makwanpur | 27.6441 | 85.1184 | 1701m |
| 46 | CO 14669 | Dolakha | 27. 64993311 | 85. 960618 | 2200m |
| 47 | CO 14962 | Lalitpur | 27. 479894 | 85. 236181 | 1700m |
| 48 | CO 15426 | Okhaldhunga | 27. 307183 | 86. 330297 | 1250m |
| 49 | CO 14593 | Sindhupalchowk | 27. 683442 | 85. 892030 | 2000m |
| 50 | CO 14956 | Lalitpur | 17. 492007 | 85. 247828 | 1700m |
| 51 | CO 15042 | Baglung | 28. 131 | 83. 372 | 1840m |
| 52 | CO 14915 | Dhading | 27. 80204 | 84. 880560 | 462m |
| 53 | CO 14570 | Sindhupalchowk | 27. 677912 | 86. 934377 | 2500m |
| 54 | CO 15473 | Bhojpur | 27. 165 | 87.055 | 1500m |
| 55 | CO 13374 | Dhankhuta | 27. 00091 | 87. 3087 | 1371m |
| 56 | Trishuli | Dolakha | 27.46 | 86.10 | 1596m |
| 57 | NGRCO 8500 | Dolakha | 27.46 | 86.10 | 1596m |
| 58 | NGRCO 8503 | Dolakha | 27.46 | 86.10 | 1727m |
