# Supplementary material for: Morphological and molecular characterization of Nepalese Common Bean (Phaseolus vulgaris L.) Landraces
Source: PLoS One. 2026 Jul 30;21(7):e0354479. doi: 10.1371/journal.pone.0354479 (PMC13423178; doi:10.1371/journal.pone.0354479)
Supplement: S2 Table — (DOCX) [file pone.0354479.s002.docx]

S2 Table. List of 23 quantitative characters studied among the common bean landraces

| Quantitative traits | Unit | Crop growth stage | Description of character |
| --- | --- | --- | --- |
| Days to 80% germination | count | Vegetative stage | Number of days taken by seed to germinate by 80% |
| Chlorophyll content | CCI | Vegetative stage | Measured from 5 plants from different positions |
| Leaf length | cm | Vegetative stage | Measured on terminal leaflet of third trifoliate leaf from pulvinus to leaf tip |
| Leaf breadth | cm | Vegetative stage | Measured on terminal leaflet of third trifoliate leaf across the leaf diameter |
| Days to first flowering | count | Reproductive stage | Number of days from emergence to stage where 50% of plants have set flowers |
| Days to 50% flowering | count | Reproductive stage | Number of days from emergence to stage where first plant set flowers |
| Dyas to first fruiting | count | Reproductive stage | Number of days from emergence to stage where first plant set fruit |
| Number of flower bud/raceme | count | Reproductive stage | Average number of flower buds counted from 5 random plants raceme |
| Number of raceme/plants | count | Reproductive stage | Average number of racemes counted from 5 random plants |
| Pod length | mm | Reproductive stage | Average length of the largest fully expanded immature pods measured from 5 random plants by vernier caliper |
| Pod width | mm | Reproductive stage | Average width of the largest fully expanded immature pods measured from 5 random plants by vernier caliper |
| Plant height | cm | Maturity stage | Measured from cotyledon scar to the tip of plant |
| Stem diameter | mm | Maturity stage | Measured by vernier caliper at crop maturity |
| Number of nodes/plants | count | Maturity stage | Counting the number of nodes from 5 random plants |
| Number of pods/plants | count | Maturity stage | Average number of pods from 5 plants |
| Number of seed/pods | count | Maturity stage | Average number of seed from one pod |
| Days to maturity | count | Maturity stage | Number of days from emergence until 90% of pod are mature |
| Seed length | mm | After harvesting stage | Measured parallel to the hilum by vernier caliper |
| Seed breadth | mm | After harvesting stage | Measured the diameter between the seed by vernier caliper |
| Seed thickness | mm | After harvesting stage | Measured from hilum to opposite site by vernier caliper |
| Thousand seed weight | g | After harvesting stage | Weight of 100 seed measured by weighing balance at moisture content of 12-14% |
| Total yield | t/ha | After harvesting stage | Converting the gram per meter square into tons per hectare |
