# Supplementary material for: Morphological and molecular characterization of Nepalese Common Bean (Phaseolus vulgaris L.) Landraces
Source: PLoS One. 2026 Jul 30;21(7):e0354479. doi: 10.1371/journal.pone.0354479 (PMC13423178; doi:10.1371/journal.pone.0354479)
Supplement: S3 Table — (DOCX) [file pone.0354479.s003.docx]

S3 Table. List of 24 qualitative characters studied among the common bean landraces

| Qualitative traits | Crop growth stage | Description of character |
| --- | --- | --- |
| Hypocotyl pigmentation | Vegetative stage | 1=Purple, 2=green |
| Emerging cotyledon color | Vegetative stage | 3=Green, 4=white, 6=others |
| Leaf color of chlorophyll | Vegetative stage | 3=Pale green, 5=medium green 7=dark green |
| Leaf hairiness | Vegetative stage | 3=Low, 5=medium, 7=dark green |
| Leaf shape | Vegetative stage | 1=Triangular, 2=quadrangular |
| Flower color | Reproductive stage | 1=White, 3=lilac, 7=dark lilac with purple spots |
| Plant type | Reproductive stage | 1=Determinate bush, indeterminate bush with erect branches, 3=Indeterminate bush with produce branches, 4=Indeterminate with semi climbing main stem and branching, 5= Indeterminate with moderate climbing ability and pods are distributed evenly upto the plants, 6=Indeterminate with aggressive climbing and pods are mainly on the upper nodes of the plant |
| Bracteole size | Reproductive stage | 3= Small, 5=medium, 7=large |
| Bracteole shape | Reproductive stage | 3= Lanceolate, 7=Ovate |
| Bracteole/calyx length relation | Reproductive stage | 3=Shorter than or equal to, 5=up to 1/3 longer |
| Calyx/bracteole color | Reproductive stage | 1=Green, 2=pale violet, 3-dark purple |
| Flower bud size | Reproductive stage | 3=Small, 5=medium, 7-large |
| Pod curvature | Reproductive stage | 3-Straight, 5=slightly curved, 7=curved |
| Pod beak orientation | Reproductive stage | 3=Upward, 5=straight, 7=downward |
| Pod beak position | Reproductive stage | 1=Marginal, 2=non-marginal, 3=others |
| Wing/pod opening | Reproductive stage | 3=Parallel closed wings, 5=wings moderately diverging, 7=wings widely diverging |
| Position of pod | Reproductive stage | 1=base, 4=combination of 1,2 and 3 |
| Pod color | Maturity stage | 1=Dark purple, 3=purple strips on green, 4=carmine strips on green, 6=dark pink, 7=normal green, 8=shiny green, 9=dull green or silver grey |
| Seed coat pattern | After harvesting | 0=Absent, 1=constant mottled, 2=stripped, 3=rhomboid spotted, 4=speckled, 5=circular mottling, 7=broad striped |
| Seed coat color | After harvesting | 1=Black, 2=brown pale to dark, 3=maroon, 5=yellow to greenish yellow, 6=pale cream to buff, 7=pure white, 8=whitish, 9=white purple tinged, 12=red |
| Seed shape | After harvesting | 1=Round, 2=oval, 3=cuboid, 4=kidney shaped, 5=truncate fastigiate, 3=matt, 5=medium, 7=shiny |
| Brilliance of the seed | After harvesting | 3=matt, 5=medium, 7=shiny |
| Number of seed color | After harvesting | 1=Black, 2=brown pale to dark, 3=maroon, 4=gray, brownish to greenish, 5=yellow to greenish yellow |
| Primary/main seed color | After harvesting | 1-Black, 2-brown, pale to dark, 3-maroon, 5-yellow to greenish yellow, 6-pale cream to buff, 7-pure white, 8-whitish, 9-white, purple tinged, 12-red |
| Secondary seed color | After harvesting | 1=Black, 2=brown, pale to dark, 3=maroon, 5=yellow to greenish yellow, 6=pale cream to buff, 7=pure white, 8=whitish, 9=white, purple tinged, 12=red |
| Seed veining | After harvesting | +=Absent, 0=present |
| Seed hilum colour | After harvesting | 8=Whitish, 12=red |
