# Supplementary material for: Morphological and molecular characterization of Nepalese Common Bean (Phaseolus vulgaris L.) Landraces
Source: PLoS One. 2026 Jul 30;21(7):e0354479. doi: 10.1371/journal.pone.0354479 (PMC13423178; doi:10.1371/journal.pone.0354479)
Supplement: S4 Table — (DOCX) [file pone.0354479.s004.docx]

S4 Table. Details of 15 SSR primers, sequences and annealing temperature used in the research

| Marker name | Forward primer | Reverse primer | Annealing temperature |
| --- | --- | --- | --- |
| PV-cct001 | CCAACCACATTCTTCCCTACGTC | GCGAGGCAGTTATCTTTAGGAGTG | 56.9 |
| BM143 | GGGAAATGAACAGAGGAA A | ATGTTGGGAACTTTTAGTGTG | 54.5 |
| BM170 | AGCCAGGTGCAAGACCTTAG | AGATAGGGAGCTGGTGGTAGC | 52.1 |
| BM184 | AGTGCTCTATCAAGATGTGTG | ACATAATCAATGGGTCACTG | 50.5 |
| BM187 | TTTCTCCAACTCACTCCTTTCC | TGTGTTTGTGTTCCGAATTATGA | 48.1 |
| PV-BR35 | TCTACGCGTTCCCTCTGTCT | AGTGGATGTGTGGGAAAAGC | 52.1 |
| PV-BR167 | GGCAAAAACAAAACCATTTCA | GCCATTTCTCCACTGTCTGG | 43.8 |
| PV-BR185 | TGGTAAAGCAAA ACGATGG | GACAGAAGAGTGAGGGTGTGAA | 50.5 |
| SSR-IAC10 | AGGAACTAAAAGCCGAACTGG | GCCTCCGCCGATCAACACTA | 50.5 |
| SSR-IAC62 | AACCCGTGAATCTTTGAGG | ATTGATGGTGGATTTTGAA | 49.5 |
| BMd-12 | CATCAACAAGGACAGCCTCA | GCAGCTGGCGGGTAAAACAG | 52.1 |
| BMd-25 | GCAGATCGCCTACTCACAAA | CGTTGACGAGAAGCATCAAG | 55.7 |
| BMd-26 | CTTGCCTTGTGCTTCCTTCT | TCCATTCCCAACCAAGTTTC | 53.2 |
| BM152 | CCGGGACTTGCCAGAAGAAC | AAGAGGAGGTCGAAACCTTAAATCG | 50.5 |
| BM210 | CCCTCATCCTCCATTCTTATCG | ACCACTGCAATCCTCATCTTTG | 50.5 |
