# Supplementary material for: Morphological and molecular characterization of Nepalese Common Bean (Phaseolus vulgaris L.) Landraces
Source: PLoS One. 2026 Jul 30;21(7):e0354479. doi: 10.1371/journal.pone.0354479 (PMC13423178; doi:10.1371/journal.pone.0354479)
Supplement: Attachment — Submitted filename: PONE-D-26-11665_Rev-AM.pdf [file pone.0354479.s005.pdf]

PLOS One

Morphological and Molecular Characterization of Nepalese Common Bean (Phaseolus vulgaris L.) Landraces

--Manuscript Draft--

**Manuscript Number:**	PONE-D-26-11665
**Article Type:**	Research Article
**Full Title:**	Morphological and Molecular Characterization of Nepalese Common Bean (Phaseolus vulgaris L.) Landraces
**Short Title:**	Diversity in Nepalese in common bean
**Corresponding Author:**	Pradip Thapa<br>Nepal Agricultural Research Council<br>Khumaltar, Lalitpur, NEPAL
**Keywords:**	Genetic diversity; SSR markers; population structure; agromorphological traits
**Abstract:**	Nepalese common bean landraces constitute valuable yet underexplored genetic reservoirs for crop improvement. However, integrated morphological and molecular characterization of this germplasm remains limited. This study evaluated the morphological and genetic diversity of 58 Nepalese common bean landraces from 21 districts at the National Agriculture Genetic Resources Centre (NAGRC) from 2023-2024 via an augmented block design. Twenty-three quantitative and twenty-four qualitative traits were measured and analyzed via descriptive and multivariate methods. The Shannon-Weaver diversity indices (H') were 0.085-0.99 for qualitative traits and 0.46-0.90 for quantitative traits. The five principal components (PCs) explained 70.86% of the total phenotypic variation, with two PCs explaining 46.27% of the variation. Phenotypic cluster analysis grouped the 58 accessions into 5 distinct groups. Molecular analysis via 15 SSR markers revealed 40 alleles, with an average of 3.16 alleles per marker. The PIC value ranged from 0.16 to 0.46. The genotypic dendrogram grouped the 58 landraces into 3 clusters. The first two molecular principal coordinates accounted for 43.27% of the variation among the accessions. The analysis of molecular variance revealed a maximum variation of 93% within the population and only 7% variation among the populations. Population structure analysis revealed three genetic clusters, with most accessions showing admixture with moderate heterozygosity and a high fixation index. The accessions NGRCO4168 and NGRCO9516 were identified as promising for days to 50% flowering, days to 50% fruiting, maturity, and yield and should be evaluated across environments, whereas expanded SSR or SNP analyses combined with phenotypic data can support marker-trait association studies for key agronomic traits.
**Order of Authors:**	Pradip Thapa
	Sandip Bohara, B.Sc.
	Promise Shrestha, B.Sc.
	Sanjay Raut
	Bal Krishna Krishna Joshi
**Additional Information:**	
**Question**	**Response**
**Financial Disclosure**<br><br>Enter a financial disclosure statement that describes the sources of funding for the work included in this submission. Review the submission guidelines for detailed requirements. View published research articles from *PLOS ONE* for specific	The author(s) received no specific funding for this work.

examples.<br><br>This statement is required for submission and **will appear in the published article** if the submission is accepted. Please make sure it is accurate.<br><br>**Funded studies**<br>Enter a statement with the following details:<br>• Initials of the authors who received each award<br>• Grant numbers awarded to each author<br>• The full name of each funder<br>• URL of each funder website<br>• Did the sponsors or funders play any role in the study design, data collection and analysis, decision to publish, or preparation of the manuscript?<br><br>Did you receive funding for this work?	
**Competing Interests**<br><br>Use the instructions below to enter a competing interest statement for this submission. On behalf of all authors, disclose any competing interests that could be perceived to bias this work—acknowledging all financial support and any other relevant financial or non-financial competing interests.<br><br>This statement is required for submission and **will appear in the published article** if the submission is accepted. Please make sure it is accurate and that any funding sources listed in your Funding Information later in the submission form are also declared in your Financial Disclosure statement.<br><br>View published research articles from *PLOS ONE* for specific examples.	The authors have declared that no competing interests exist.

**NO authors have competing interests**<br><br>Enter: *The authors have declared that no competing interests exist.*<br><br>**Authors with competing interests**<br><br>Enter competing interest details beginning with this statement:<br><br>*I have read the journal's policy and the authors of this manuscript have the following competing interests: [insert competing interests here]*<br><br>* typeset	
**Ethics Statement**<br><br>Enter an ethics statement for this submission. This statement is required if the study involved:<br><br>• Human participants<br>• Human specimens or tissue<br>• Vertebrate animals or cephalopods<br>• Vertebrate embryos or tissues<br>• Field research<br><br>Write "N/A" if the submission does not require an ethics statement.<br><br>General guidance is provided below. Consult the submission guidelines for detailed instructions. **Make sure that all information entered here is included in the Methods section of the manuscript.**	No permission required

Powered by Editorial Manager® and ProduXion Manager® from Aries Systems Corporation

**Format for specific study types**<br><br>**Human Subject Research (involving human participants and/or tissue)**<br>• Give the name of the institutional review board or ethics committee that approved the study<br>• Include the approval number and/or a statement indicating approval of this research<br>• Indicate the form of consent obtained (written/oral) or the reason that consent was not obtained (e.g. the data were analyzed anonymously)<br><br>**Animal Research (involving vertebrate animals, embryos or tissues)**<br>• Provide the name of the Institutional Animal Care and Use Committee (IACUC) or other relevant ethics board that reviewed the study protocol, and indicate whether they approved this research or granted a formal waiver of ethical approval<br>• Include an approval number if one was obtained<br>• If the study involved *non-human primates*, add *additional details* about animal welfare and steps taken to ameliorate suffering<br>• If anesthesia, euthanasia, or any kind of animal sacrifice is part of the study, include briefly which substances and/or methods were applied<br><br>**Field Research**<br><br>Include the following details if this study involves the collection of plant, animal, or other materials from a natural setting:<br>• Field permit number<br>• Name of the institution or relevant body that granted permission	
**Data Availability**<br><br>Authors are required to make all data underlying the findings described fully available, without restriction, and from the time of publication. PLOS allows rare exceptions to address legal and ethical concerns. See the PLOS Data Policy and FAQ for detailed information.	No - some restrictions will apply

A Data Availability Statement describing where the data can be found is required at submission. Your answers to this question constitute the Data Availability Statement and **will be published in the article**, if accepted.<br><br>**Important:** Stating 'data available on request from the author' is not sufficient. If your data are only available upon request, select 'No' for the first question and explain your exceptional situation in the text box.<br><br>Do the authors confirm that all data underlying the findings described in their manuscript are fully available without restriction?	
**Describe where the data may be found in full sentences. If you are copying our sample text, replace any instances of XXX with the appropriate details.**<br><br>• If the data are **held or will be held in a public repository**, include URLs, accession numbers or DOIs. If this information will only be available after acceptance, indicate this by ticking the box below. For example: *All XXX files are available from the XXX database (accession number(s) XXX, XXX.).*<br>• If the data are all contained **within the manuscript and/or Supporting Information files**, enter the following: *All relevant data are within the manuscript and its Supporting Information files.*<br>• If neither of these applies but you are able to provide **details of access elsewhere**, with or without limitations, please do so. For example:<br><br>*Data cannot be shared publicly because of [XXX]. Data are available from the XXX Institutional Data Access / Ethics Committee (contact via XXX) for researchers who meet the criteria for access to confidential data.*<br><br>*The data underlying the results presented in the study are available from (include the name of the third party*	Data are not available at this time

and contact information or URL).<br>• This text is appropriate if the data are owned by a third party and authors do not have permission to share the data.<br><br>* typeset	
Additional data availability information:	

Manuscript Click here to access/download;Manuscript;Manuscript.docx

Morphological and Molecular Characterization of Nepalese Common Bean *(Phaseolus vulgaris* L.*)* Landraces

Pradip Thapa[✉] **, S. Bohara**[2]**, P. Shrestha**[3]**, Sanjay Raut**[2]**, B. K. Joshi**[1]

[1]National Agriculture Genetic Resources Centre, Khumaltar, Lalitpur, Nepal
[2]Far Western University, Tikapur, Nepal
[3]Agriculture and Forestry University, Chitwan, Nepal

1) Pradip Thapa
Email: pradip.thapa876@gmail.com
ORCiD ID: https://orcid.org/0000-0002-2080-6517
Address: Kathmandu, Bagmati, Nepal

2) Sandip Bohara
Email: sandeepbohara547@gmail.com
ORCiD ID: https://orcid.org/0009-0002-2453-5117
Address: Kathmandu, Bagmati, Nepal

3) Promise Shrestha
Email: sthapromise0584@gmail.com
ORCiD ID: https://orcid.org/0009-0007-6215-8398
Address: Kathmandu, Bagmati, Nepal

4) Sanjay Kumar Raut
Email: sanjayraut309@gmail.com
ORCiD ID: https://orcid.org/0009-0001-9229-4821
Address: Tikapur, far western, Nepal

5) Bal Krishna Joshi
Email: joshibalak@yahoo.com
ORCiD ID: https://orcid.org/0000-0002-7848-5824
Address: Kathmandu, Bagmati, Nepal

Corresponding author: Pradip Thapa
Institute: National Agricultural Genetic Resources, Gene Bank, Nepal
Email: pradip.thapa876@gmail.com

Abstract

Nepalese common bean landraces constitute valuable yet underexplored genetic reservoirs for crop improvement. However, integrated morphological and molecular characterization of this germplasm remains limited. This study evaluated the morphological and genetic diversity of 58 Nepalese common bean landraces from 21 districts at the National Agriculture Genetic Resources Centre (NAGRC) from 2023-2024 via an augmented block design. Twenty-three quantitative and twenty-four qualitative traits were measured and analyzed via descriptive and multivariate methods. The Shannon-Weaver diversity indices (H') were 0.085-0.99 for qualitative traits and 0.46-0.90 for quantitative traits. The five principal components (PCs) explained 70.86% of the total phenotypic variation, with two PCs explaining 46.27% of the variation. Phenotypic cluster analysis grouped the 58 accessions into 5 distinct groups. Molecular analysis via 15 SSR markers revealed 40 alleles, with an average of 3.16 alleles per marker. The PIC value ranged from 0.16 to 0.46. The genotypic dendrogram grouped the 58 landraces into 3 clusters. The first two molecular principal coordinates accounted

for 43.27% of the variation among the accessions. The analysis of molecular variance revealed a maximum variation of 93% within the population and only 7% variation among the populations. Population structure analysis revealed three genetic clusters, with most accessions showing admixture with moderate heterozygosity and a high fixation index. The accessions NGRCO4168 and NGRCO9516 were identified as promising for days to 50% flowering, days to 50% fruiting, maturity, and yield and should be evaluated across environments, whereas expanded SSR or SNP analyses combined with phenotypic data can support marker-trait association studies for key agronomic traits.

Keywords: Genetic diversity, SSR markers, population structure, agromorphological traits, Nepalese bean landraces, analysis of molecular variance, principal coordinate analysis

1. Introduction

The common bean (*Phaseolus vulgaris* L.*)* is an annual, herbaceous, self-pollinating legume crop species that belongs to the family Fabacea [1]. It is a diploid and has 11 pairs of chromosomes (2n=2x=22), with a genomic size of 473 Mb [2]. The Leguminosae family ranks as the second most important group of agricultural crops, following the Poaceae family [3]. The common bean is the most extensively grown and consumed legume worldwide [4]. The Mesoamerican and Andean gene pools originated from the domestication of beans approximately 8,000 years ago [5]. Geographical and reproductive barriers partially isolate the centers of genetic diversity in the Mesoamerican and Andean regions of origin [5]. The Mesoamerican gene pool is more genetically diverse than the Andean gene pool is [6]. The Andean gene pool is characterized by large seeds (>40 g 100-seed weight^{-1}) and ‘T,’ ‘C,’ ‘H,’ and ‘A’ phaseolin types, whereas the Mesoamerican pool has smaller (<25 g) or medium-sized seeds with ‘S’ or ‘B’ phaseolin types [7, 8 , 5]. Distinct physical traits separate the Andean and Mesoamerican gene pools. Andean beans have larger leaves, lanceolate or triangular bracteoles, white flowers, and longer internodes, whereas Mesoamerican varieties are characterized by cordate or ovate bracteoles, a placental pod beak, and colorful, striped flowers [9]. There are more than 70 species of common bean, five of which, runner bean (*P. coccineus L.*), lima bean (*P. lunatus L.*), tepary bean (*P. acutifolius A. Gray*), year bean (*P. dumosus Macfady*), and common bean (*P. vulgaris L.*) have been domesticated in various ecogeographic settings [10]. Among the five domesticated varieties worldwide, common bean is the most extensively consumed grain legume in the world and makes up approximately 90% of cultivated crops [11].

The global area, yield, and productivity of common bean are 191525937 ha, 4.20 (t/ha) and 438158366.15 tons, respectively [12]. In Nepal, bean is cultivated in an area of 21,823 ha, with a production of 19117.7 tonnes and a productivity of 3.71 t/ha [12]. Nepal has a total of five common bean varieties, including three registered landraces and 2 hybrid varieties [13]. Beans are the main sources of vegetable protein in the human diet and can be eaten as dried seeds, green pods, or immature seeds [14]. It is considered "poor man's meat", as it serves as a cheap source of food for 300 million people in the tropics and 100 million people in Africa [15]. Common bean plays a significant role in subsistence farming, which is why it is considered a grain of hope [11]. Beans are highly protenious and contain 2-3 times more protein than cereal crops do [16]. Common bean has high protein (16-33%) and fiber contents; complex carbohydrates; and other nutrients, such as iron, zinc, magnesium, potassium, and folic acid, which are sources of B-complex vitamins [8]. It is used as a traditional remedy for renal issues, diabetes, dropsy, diarrhea, and dysentery [17]. Common beans improve soil fertility by fixing atmospheric nitrogen through their symbiotic association with rhizobia and the breakdown of plant residues [18].

Landraces, also known as local genotypes, are populations produced over many years by artificial or natural selection, which is distinguished by their strong tolerance to biotic and abiotic stresses, leading to stable yields and moderate productivity within low-input farming systems [19]. Landrace and wild crop accessions are important genetic resources for plant breeding and the preservation of plant alleles and gene combinations [20]. Landraces serve as valuable sources of genetic diversity, offering traits for resistance to diseases and pests, as well as tolerance to various abiotic stresses [21]. Improved landraces perform better than the national average yield of cereal crops by 20% and 60% for minor crops [22]. As the biophysical resource base declines and climate change poses increasing threats, landraces are central to current crop breeding programs and represent a vital part of plant biodiversity in common beans [23]. The world's population is predicted to grow at a high rate to 10 billion people by 2050. Therefore, to fulfill the anticipated food demand in 2050, global food production must be increased by 60% to 110% [24]. ~~Therefore, to meet the needs of the growing population, research should focus on legume crops rather than solely cereal crops~~. The National Agriculture Genetic Resources Centre (NAGRC) conserves a total of 719 pulse crop accessions, 93 of which are bean accessions [22]. Characterizing the genetic diversity of common bean accessions from germplasm banks and working collections helps determine optimal genetic distances between parent pairs, identify redundancies, and understand genetic interactions [25].

Morphological and agronomic traits are often used to assess genetic diversity, but they are strongly influenced by the environment and developmental stage, and their limited heritability makes them unreliable indicators of true genetic relatedness among accessions [26, 27]. Therefore, molecular markers are crucial techniques for genetic mapping and analysis of genetic diversity, population structure, and phylogenetic connections in many plants since they are ~~unaffected~~ by ecological conditions [28]. When these DNA molecular markers are tightly associated with genes of interest, they may be utilized in marker-assisted breeding programs to select for preferred alleles [29]. For the characterization, conservation, and management of germplasm, molecular markers offer a more straightforward, dependable, and effective method of revealing genotype variations at the DNA level [30]. Simple sequence repeats (SSRs), or microsatellites, are PCR-based, codominant, and highly polymorphic markers with 2-6 bp tandem repeats. They are widely used for variety identification, genetic mapping, diversity analysis, marker-assisted selection, and population structure studies in common bean germplasm [31, 32 , 29].

To date, there is no evidence of the molecular characterization of common bean germplasm by simple sequence repeat markers in Nepal. Therefore, this research was carried out to assess the genetic diversity and population structure among 58 bean landraces via morphological and molecular analyses~~. Therefore, the results of this study could be used for bean breeding and bean improvement programs.~~

2. Materials and methods

2.1 Study area and experimental site

The experiment was conducted at the National Agriculture Genetic Resources Center (NAGRC) GenBank, Khumaltar, Lalitpur, Nepal, during the winter season of 2023. The site lies 1368 m above sea level at 27°40' N latitude and 85°20' E longitude. During the study period (November to March), the site experienced typical winter climatic conditions, with mean maximum temperatures ranging from 18.7°C (January) to 23.9°C (November) and mean minimum temperatures ranging from 3.1°C January to 9.8°C March (Figure 1). Rainfall was low and sporadic, occurring mainly in February (13.9 mm) and March (44.3 mm). Overall, the region receives approximately 29.1 mm of annual rainfall, with average minimum and maximum temperatures of 6.5°C and 21.3°C, respectively.

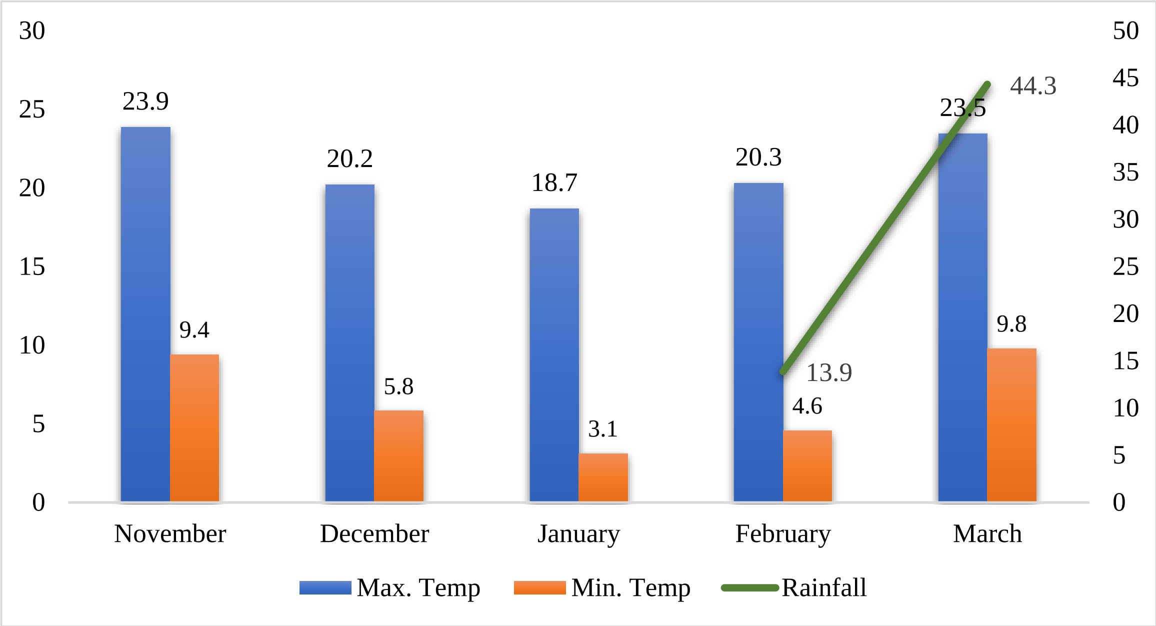

Figure 1. Meteorological data of the study site

2.2 Plant materials

A total of 58 common bean accessions collected from 21 districts (Annex Table 1; Figure 2) across Nepal, were included in the study to represent wide genetic and geographic diversity. Three varieties, NGRCO8500, NGRCO8503, and Trishuli, served as check entries. Five accessions were collected from the Terai region, 44 accessions were collected from the hilly region, and 9 accessions were collected from the hill hilly area of Nepal.

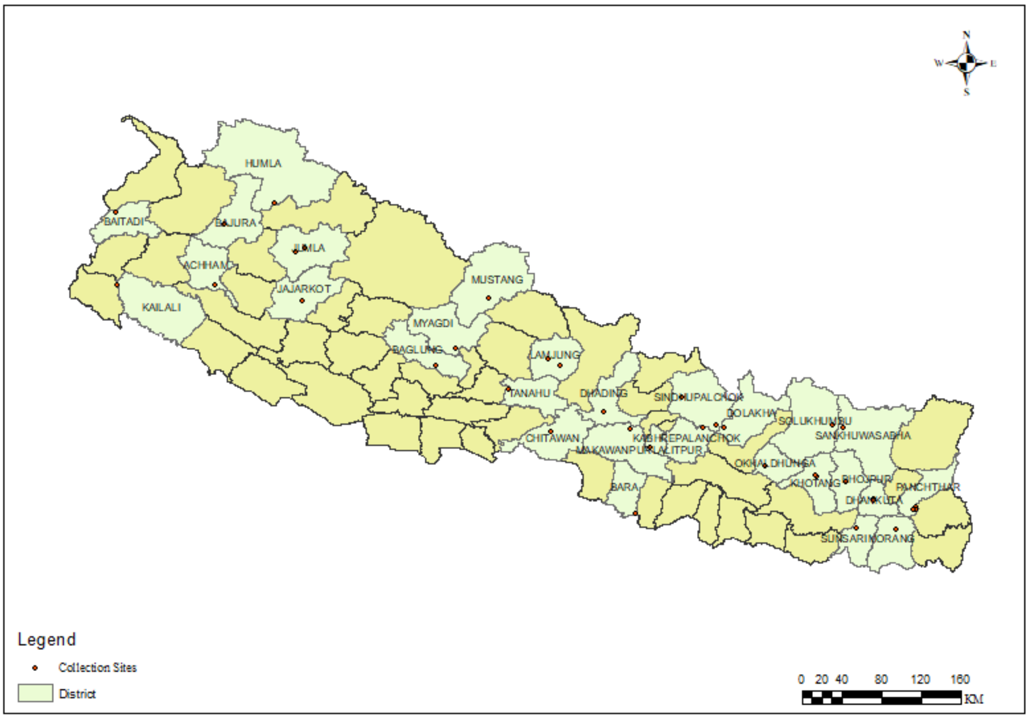

Figure 2. ~~Collection sites of the bean landraces~~

~~2.3 Experimental design and layout~~

The experiment was arranged in an augmented block design with two replications. Seeds were sown on 12 November 2023. Each accession was planted in two rows, each measuring 0.9 m in width and 3 m in length. Ten seeds were sown per row, maintaining a spacing of 60 cm between rows and 30 cm between plants.

~~2.4~~. Agronomic and crop management practices

The experimental field was prepared through plowing and harrowing to obtain a fine tilth. Farmyard manure was applied at 10 t ha^{-1} during final land preparation. Fertilizers were applied at the recommended rate of 50:60:40 kg ha^{-1} N:P:K [13], with the full dose of phosphorus and potassium and half of the nitrogen applied basally at sowing, and the remaining nitrogen was top-dressed at 30 days after sowing (DAS). Hand weeding was carried out twice, at 25-30 DAS and 45-50 DAS. Irrigation was provided as needed, particularly during flowering and pod development. Staking at 30-35 DAS was performed via bamboo stakes to support indeterminate and semi-climbing plants. Plant protection measures were applied uniformly following need-based integrated pest management practices.

~~2.5~~ Data collection

Morphological data ~~of~~ 23 quantitative and 24 qualitative traits were recorded via standard descriptors for common bean [33]. The qualitative traits were recorded on the basis of the observation of descriptor states (Annex Table 3), whereas the quantitative traits were recorded by count ~~and~~ measurement (Annex Table 2). Observations of quantitative traits were taken from 5 randomly selected plants and seeds.

~~2.6 Morphological data analysis~~

~~The qualitative and quantitative data were recorded and entered into Microsoft Excel for further analysis. The combined mean~~ quantitative data were analyzed for descriptive statistics (minimum, maximum, mean, variance, standard deviation and coefficient of variance) via the pastecs [34] package in R studio (4.4.3) [35]. The qualitative data were analyzed via package summary tools [36]. Shannon’s waver diversity index (H’) [37] was also calculated via a specific formula in Microsoft Excel (2016).

$$H' = [\Sigma \binom{n}{N} \times \{Log2 \binom{n}{N} \times (-1)\}] / Log2\kappa$$

where n represents the frequency of a phenotypic trait class, N is the total number of observations for a particular trait, and κ denotes the number of phenotypic classes. The quantitative traits were classified into 9 intervals, with the diversity indices categorized into four levels: low (0.1-0.4), intermediate (0.4-0.6), high (0.6-0.8), and very high (>0.8) [38]. Principal component analysis (PCA) was performed via the Factoextra [39], factominor [40] and gridextra [41] packages. The optimum number of clusters was selected via the Factoextra package [39] and the Nbclust package [42]. The circular hierarchical cluster dendrogram was generated via the average (UPGMA) method, and the Euclidean distance was determined via the dendextend [43] and circilize [44] packages.

~~2.7 Molecular data analysis~~

The SSR bands were scored on the basis of the presence and absence of the bands, the 1 and 0 scoring and band size were performed, ~~and the data were entered into Microsoft Excel (2016)~~. The molecular hierarchical cluster dendrogram was prepared ~~via~~ the average method and Nei’s genetic distance (1973) via NTsyspc software [45]. Principal coordinate analysis (PCoA) and analysis of molecular variance (AMOVA) were performed via Gene Alex (6.0) software [46]. The PIC value was calculated as PIC= $1 - \sum pi^2$, where p represents the frequency of an allele [47]. The frequency of alleles,

observed heterozygosity, expected heterozygosity, polymorphic information content and fixation index were calculated via Power Marker (3.0) software [48]. The population structure analysis and expected heterozygosity and fixation index (F_{st}) calculations for each subpopulation were performed via STRUCTURE software V.2.3.4 [49]. The optimum number of clusters was selected by implementing the structure selector [50] and Evanno's ΔK method [51]. The analysis involved Markov Chain Monte Carlo (MCMC) simulations by setting the k value from 2 to 10 with 5 iterations, with burn-in periods of 10000 and 100,0000. The percentage of polymorphisms, marker index, resolving power, and effective multiplex ratio were calculated via specific formulas in Microsoft Excel. The percentage of polymorphism was calculated by POP =Number of polymorphic bands/Total number of bands. The resolving power was calculated via the formula RP= Ib= $1 - (2 \times 0.5 - P\,|)$, where P represents the proportion of individuals containing bands and Ib= Bands informativeness for each band (Prevost and Wilkinson 1999). The effective multiplex value was calculated via the formula EMR= Number of polymorphic bands × no. of polymorphic bands/Total number of bands [47]. The marker index was calculated via the formula MI=PIC × EMR, where PIC=polymorphic information content and EMR=effective multiplex [47].

3. Molecular characterization

In total, 58 leaf samples of different bean accessions were taken from the field of the National Agricultural Genetic Resources Center (NAGRC), GenBank. Approximately 0.2 g of leaf samples from 15-20 day old plants were taken, and DNA was extracted according to the modified CTAB protocol [52, 53]. The DNA concentration was quantified by using the A260/A280 ratio with a Quawell UV-Vis spectrophotometer Q5000S. The extracted DNA was stored at -200°C for PCR. In total, 15 SSR primer pairs (Annex Table 5) were used in this study to assess the genetic diversity among the bean accessions. PCR analysis was carried out in a total reaction volume of 15 µL, including 7.5 µL of master mix 2X Green GoTaq® (Promega 25 ml), 1.5 µL of forward and 1.5 µL of reverse marker (Promega 25 ml), 2.5 µL of nuclease-free water (Promega 25 ml), and 2 µL of DNA ~~utilizing~~ the MiniAmp thermal cycler ~~with a single 96-well plate~~. The DNA sample was then diluted to a final concentration of 50 ng/µl for PCR. The markers were also diluted to a final concentration of 250 ng/µl. The specific denaturation, annealing and extension temperatures are given in Annex Table 6. The PCR products were separated on a 1.5% agarose gel (BIONEER 500 gm) using 1x TAE buffer (GENAXXON bioscience 500 ml) in 300 ml and 400 ml of 1x TAE buffer. Approximately 2 µL/100 ml of ethidium bromide (EtBr) (GENAXXON bioscience) was used for gel preparation. The electrophoresis running buffer (1X TAE) was prepared by diluting 20 ml of 50X TAE stock solution to 1 L with distilled water. A total of 15 µL of PCR product was loaded in the gel, and 1 kb and 100 bp of the ladder (GENAXXON bioscience) DNA was also added for scoring of the DNA bands. Then, the gel was run for 2 hours at 90 volts and 50 mA current. The gel was subsequently visualized under UV light (PowerPro Cleaver Scientific) to observe the bands.

4. Results

4.1 Quantitative data analysis

~~Quantitative analysis of 23 traits~~ of the bean germplasm revealed considerable variation across phenological, yield, and yield-related traits, as evidenced by their observed ranges, mean values, standard errors, coefficients of variation (CVs), and Shannon-Waver diversity indices (H′) (Table 1). The coefficient of variance ranged from 10% to 68%, which indicates low to high variation among the quantitative traits. Very high variability (>25%) was found for six traits: total yield (68%), plant height (52%), number of pods per plant (46%), thousand-seed weight (42%), chlorophyll content at the vegetative

stage (30%) and number of raceme/plant (28%). Shannon Weaver's index ranged from 0.46-0.90, which represented a moderate to very high level of diversity. Very high diversity (>80%) was found for 12 quantitative traits: leaf breadth (0.92), leaf length (0.9), days to first fruiting (0.9), seed length (0.87), seed thickness (0.87), days to 50% flowering (0.86), days to 80% maturity (0.85), days to first flowering (0.84), days to 80% germination (0.83), stem diameter (0.83), days to 50% fruiting (0.82) and plant height (0.82). Similarly, [54] and [55] reported H' diversity indices for quantitative traits ranging from 0.47-0.73 and 0.36-0.97, respectively.

Early phenological traits exhibited substantial variability, with number of days to 80% germination ranging from 18-30 days (mean ± SE: 21.46 ± 0.32) and very high Shannon-Weaver diversity (H′ = 0.83). The flowering traits displayed comparatively greater heterogeneity, as number of days to first flowering varied from 50-83 days (62.15 ± 1.27), and number of days to 50% flowering ranged from 52-87 days (69.51 ± 1.49), both of which presented very high diversity (H′ = 0.84 and 0.86, respectively). [56] reported 35-70 days for number of days to first flowering and 40-84 days for days to 50% flowering, and [57] reported 40-63 days for days to 50% flowering. The reproductive phenological traits showed pronounced heterogeneity in terms of fruiting initiation and developmental duration. The number of days to first fruiting ranged from 53 to 85 days (66.34 ± 1.21), with the highest diversity among the phenological traits (H′ = 0.90), whereas the number of days to 50% fruiting (56-95 days; 75.48 ± 1.60) and the number of days to 80% maturity (85-123 days; 102.70 ± 1.35) also exhibited very high diversity (H′ = 0.82 and 0.85), respectively. [56] reported that the number of days to 80% maturity ranged from 80-149 days.

The yield-contributing traits demonstrated wide phenotypic variation. The pod length ranged from 5.95 to 15.13 cm (9.58 ± 0.28), with moderate diversity (H′ = 0.64), whereas the number of pods per plant varied from 4 to 27 (10.56 ± 0.64), indicating high diversity (H′ = 0.76). The number of seeds per pod ranged from 3 to 9 (4.86 ± 0.13), with moderate diversity (H′ = 0.46), which is comparable to earlier reports by [57], who reported 9–32 pods per plant and 3–6 seeds per pod. Thousand-seed weight showed pronounced variability, ranging from 131.25 to 1236.25 g (344.42 ± 18.86) with high diversity (H′ = 0.75), corroborating earlier findings by [56] and [57], who reported mean values of 766 g and 969.5 g, respectively. The total yield varied widely from 0.02 to 2.01 t ha^{-1} (0.85 ± 0.08) and exhibited high diversity (H′ = 0.74).

Table 1. Descriptive statistics among 23 quantitative traits of bean landraces

Accession name	Min	Max	Mean±SE	STD	CV (%)	H'
Days to 80% germination	18	30	21.46±0.32	2.41	11	0.83
Chlorophyll content at vegetative stage	11.74	41.5	21.54±0.86	6.56	30	0.67
Leaf length (cm)	6.18	10.6	8.17±0.13	1.02	13	0.90
Leaf breadth (cm)	3.27	6.89	5.10±0.10	0.73	14	0.92
Days to first flowering	50	83	62.15±1.27	9.69	16	0.84
Days to 50% flowering	52	87	69.51±1.49	11.35	16	0.86
Dyas to first fruiting	53	85	66.34±1.21	9.24	14	0.90
Days to 50% fruiting	56	95	75.48±1.60	12.15	16	0.82
Number of flower bud/raceme	2	5	3.72±0.08	0.62	17	0.66
Number of raceme/plant	5	14	8.20±0.30	2.32	28	0.75
Pod length (mm)	5.95	15.13	9.58±0.28	2.14	22	0.64
Pod width (mm)	7.54	16.26	9.97±0.19	1.48	15	0.80
Plant height (cm)	20.65	178.60	91.04±6.26	47.66	52	0.82
Stem diameter (mm)	4.00	8.01	6.23±0.10	0.77	12	0.83
Number of nodes/plant	4	8	5.63±0.15	1.15	20	0.62
Number of pods/plant	4	27	10.56±0.64	4.90	46	0.76
Number of seed/pod	3	9	4.86±0.13	1.00	21	0.46

Days to 80% maturity	85	123	102.70±1.35	10.31	10	0.85
Seed length (mm)	9.90	20.35	12.97±0.24	1.79	14	0.87
Seed breadth (mm)	5.72	13.60	7.28±0.15	1.11	15	0.80
Seed thickness (mm)	3.95	9.06	5.48±0.11	0.84	15	0.87
Thousand seed weight (g)	131.25	1236.25	344.42±18.86	143.65	42	0.75
Total yield (t/ha)	0.02	2.01	0.85±0.08	0.58	68	0.74

Min = minimum, Max = maximum, STD = standard deviation, CV = coefficient of variation, H' = Shannon–Weaver diversity index.

4.2 Qualitative data analysis

The ~~morphological descriptors,~~ frequency, percentage and Shannon waver diversity index of 24 qualitative traits of 58 bean landraces are presented in Table 2. The diversity index (H') ranged from 0.085-0.99, which indicates that low to very high diversity is present among the 24 qualitative traits of the bean landraces. The highest diversity was found for leaf shape (0.99), and the lowest diversity was found for seed coat color (0.085). Only three traits, leaf shape (0.99), bracteole shape (0.99), and hypocotyl pigmentation (0.94), presented very high diversity (>80%). Similarly, [54] and [55] reported that the Shannon waver diversity index for qualitative data ranges from 0.57-0.58 to 0.47-0.57.

~~Qualitative characterization of the bean germplasm revealed appreciable phenotypic variation across vegetative, floral, pod, and seed-related traits, as reflected by differences in frequency distribution and the Shannon–Wiener diversity index (H′).~~ The leaf chlorophyll color exhibited moderate diversity (H′ = 0.63), with medium green leaves predominating (53.45%), followed by dark green (27.59%) and pale green (18.97%), whereas the leaf shape presented comparatively greater diversity (H′ = 0.99) because of the nearly equal distributions of triangular (53.45%) and quadrangular (46.55%) forms. Flower color displayed relatively low diversity (H′ = 0.32), mainly represented by lilac (43.10%) and white (36.21%) flowers. The plant growth habit exhibited limited diversity (H′ = 0.24), with determinate bush (44.83%) and indeterminate semi-climbing types (24.14%) being the most common. Pod color showed moderate variation (H′ = 0.28), dominated by shiny green (43.10%) and normal green (31.03%) pods, whereas pigmented pod classes were relatively rare. Seed-related qualitative traits generally exhibited low diversity, as indicated by low H′ values for the seed coat pattern (0.11), seed coat color (0.085), primary seed color (0.085), and number of seed colors (0.14), with, pale cream to buff, brown, and black seeds being predominant. Seed shape exhibited moderate diversity (H′ = 0.24), with cuboid seeds accounting for the largest proportion (56.90%), followed by oval and truncate fastigiate forms.

Table 2. Qualitative data analysis of 24 traits

Variables	Descriptor states	Frequency (n)	Percentage (%)	H'
Hypocotyl pigmentation	1-Purple	12	20.69	0.94
	2-Green	46	79.31	
Emerging cotyledon color	3-Green	32	55.17	0.39
	4-Very pale green	25	43.1	
	6-other	1	1.72	
Leaf color of chlorophyll	3-Pale green	11	18.97	0.63
	5-Medium green	31	53.45	
	7-Dark green	16	27.59	
Leaf hairiness	3-low	7	12.07	0.45

	5-Medium	43	74.14	
	7-High	8	13.79	
Leaf shape	1-Triangular	31	53.45	0.99
	2-Quadrangular	27	46.55	
Flower color	1-White	21	36.21	0.32
	3-Lilac	25	43.1	
	7-Dark lilac with purplish spots	3	5.17	
	9-Purple	9	15.52	
Plant type	1-Determinate bush	26	44.83	0.24
	2-Indeterminate bush with erect branches	5	8.62	
	3-Indeterminate bush with prostate branches	2	3.45	
	4-indeterminate with semi-climbing main stem and branches	14	24.14	
	5-indeterminate with moderate climbing ability and pods are distributed evenly up to the plant	3	5.17	
	6-indeterminate with aggressive climbing and pods are mainly on the upper nodes of the plant.	8	13.79	
Bracteole size	3-Small	21	36.21	0.58
	5-Medium	28	48.28	
	7-Large	9	15.52	
Bracteole shape	3-Lanceolate	28	48.28	0.99
	7-Ovate	30	51.72	
Flower bud size	3-Small	12	20.69	0.66
	5-Medium	26	44.83	
	7-Large	20	34.48	
Pod curvature	3-Straight	12	20.69	0.65
	5-Slightly curved	28	48.28	
	7-Curved	18	31.03	
Pod beak orientation	3-Upward	20	34.48	0.61
	5-Straight	27	46.55	
	7-Downward	11	18.97	
Pod beak position	1-Marginal	32	55.17	0.39
	2-Non-marginal	25	43.1	
	3-Others	1	1.72	
Wing/pod opening	3-Parallel closed wings	20	34.48	0.58
	5-Wings moderately diverging	29	50	
	7-Wings widely diverging	9	15.52	
Pod color	1-Dark purple	1	1.72	0.28
	3-Purple strips on green	2	3.45	
	4-Carmine strips on green	7	12.07	
	6-Dark pink	1	1.72	
	7-Normal green	18	31.03	
	8-Shiny green	25	43.1	

	9-Dull green or silver grey	4	6.9	
Seed coat pattern	0-Absent	31	53.45	0.11
	1-Constant mottled	1	1.72	
	2-Stripped	15	25.86	
	3-Rhomboid spotted	3	5.17	
	4-Speckled	4	6.9	
	5-Circular mottling	3	5.17	
	7- Broad striped	1	1.72	
Seed coat color	1-Black	10	17.24	0.085
	2-Brown pale to dark	14	24.14	
	3-Maroon	8	13.79	
	5-Yellow to greenish yellow	3	5.17	
	6-Pale cream to buff	15	25.86	
	7-Pure white	2	3.45	
	8-Whitish	3	5.17	
	9-White purple tinged	1	1.72	
	12-Red	2	3.45	
Seed shape	1-Round	4	6.9	0.24
	2-Oval	12	20.69	
	3-Cuboid	33	56.9	
	4-Kidney shaped	3	5.17	
	5-Truncate fastigiate	6	10.34	
Brilliance of the seed	3-Matt	2	3.45	0.64
	5-Medium	27	46.55	
	7-Shiny	29	50	
Number of seed color	1-Black	23	39.66	0.14
	2-Brown pale to dark	19	32.76	
	3-Maroon	12	20.69	
	4-Gray, brownish to greenish	2	3.45	
	5-Yellow to greenish yellow	2	3.45	
Primary/main seed color	1-Black	9	15.52	0.085
	2-Brown, pale to dark	15	25.52	
	3-Maroon	7	12.07	
	5-Yellow to greenish yellow	3	5.17	
	6-Pale cream to buff	16	27.59	
	7-Pure white	2	3.45	
	8-Whitish	3	5.17	
	9-White, purple tinged	1	1.72	
	12-Red	2	3.45	
Secondary seed color	1-Black	2	5.56	0.090
	2-Brown, pale to dark	5	13.89	
	3-Maroon	11	30.56	
	5-Yellow to greenish yellow	4	11.11	
	6-pale cream to buff	8	22.22	

	12-Red	3	8.33	
	13-Pink	1	2.78	
	14-Purple	2	5.56	
Seed veining	+-Absent	48	82.76	0.66
	0-Present	10	17.24	
Seed hilum color	8-Whitish	52	89.66	0.48
	12-Red	6	10.34	

4.3 Principal component analysis

The results of the principal component analysis (PCA) of twenty-three quantitative characteristics of the bean landraces are presented in Table 3. Dimension-1 contains the most variance (28.49%) in the whole dataset, followed by dimension-2 (17.77%), with a cumulative variance of 46.27%. The first principal component (PC-1) is highly shaped by phenology, vegetative growth, and yield performance. Days to 50% fruiting (-0.360), days to 50% flowering (-0.358), days to first flowering (-0.329), days to 80% maturity (-0.283), plant height (-0.273), number of racemes per plant (-0.265), total yield (0.240) and number of nodes per plant (-0.273). The second principal component (PC-2) is highly dominated by seed characteristics and physiological attributes. Thousand-seed weight (0.415), seed breadth (0.405), seed thickness (0.352), number of flower bud per raceme (-0.257), stem diameter (-0.256) and chlorophyll content at the vegetative stage (0.221) were used. The first five principal components with eigenvalues greater than 1 accounted for 76.53% of the total variation, with PC-5 explaining more than ~70% of the variation (PC1: 28.49%, PC2: 17.17%, PC3: 10.39%, PC4: 8.28, PC5: 5.93).

The PCA biplot (Figure 3) revealed considerable phenotypic diversity among the bean landraces. In PC1, accessions such as NGRCO10335, NGRCO10644, NGRCO1765, and CO14741 exhibited strong associations with reproductive and yield-related traits, including days to 50% flowering, days to first fruiting, days to 50% fruiting, number of pods per plant, and number of seeds per pod. Along PC2, only a limited number of accessions showed clear separation, most notably CO14570, suggesting that a distinct phenotypic profile is influenced primarily by traits related to seeds: seed length, seed breadth, seed thickness and thousand-seed weight.

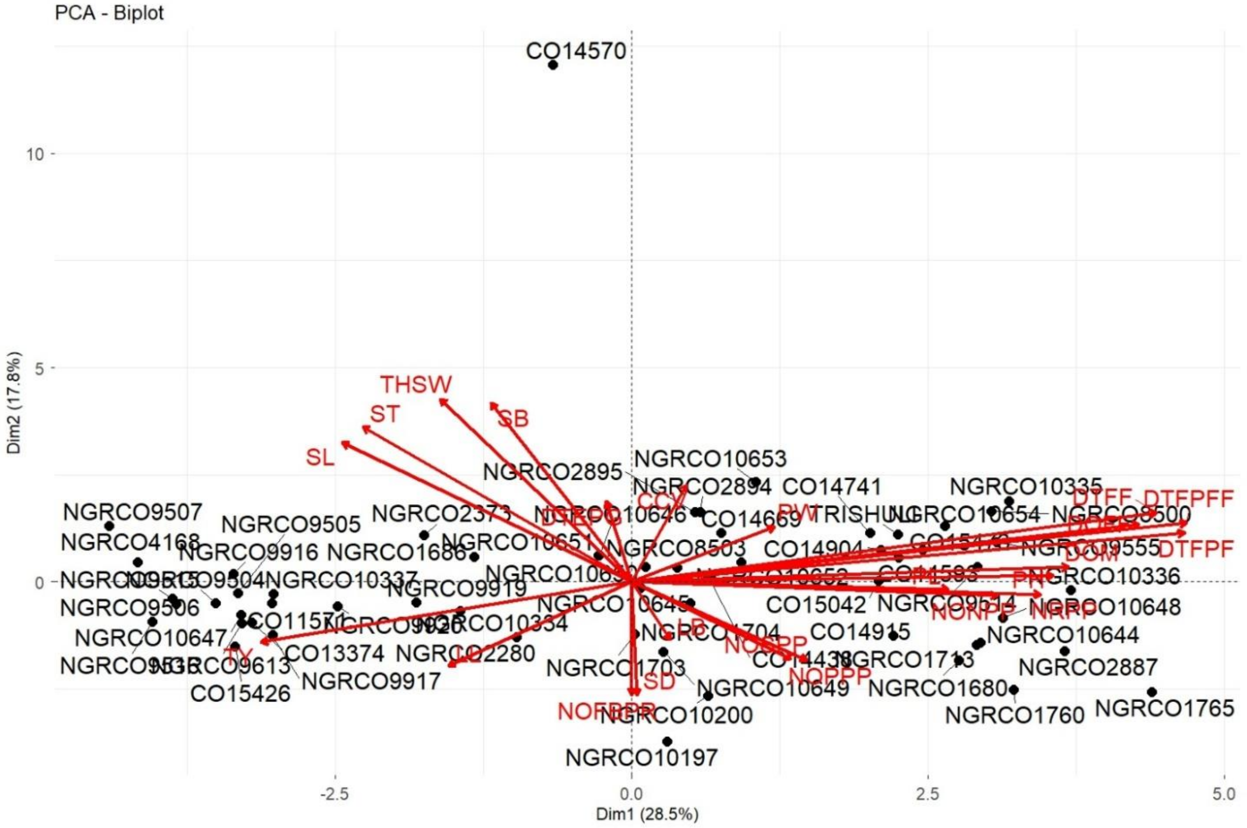

Figure 3. PCA biplot of common bean landraces

Dimensions one and two were explained primarily by phenological traits, seed characteristics, vegetative attributes and yield-related traits. Days to 50% fruiting, days to 50% flowering, thousand seed weight, days to first fruiting, seed breadth, days to first flowering, leaf breadth, leaf length, seed thickness, number of pods per plant, pod length, seed length and chlorophyll content at the vegetative stage. These results are in agreement with those of various other studies [56] , [58], [59]. Our study's six principal components are quite comparable to those reported by [60], who reported that the overall number of the six components accounts for 83% of the variation. ~~PC 3 was associated with mainly leaf and pod morphological traits as well as early establishment characteristics. Leaf breadth (-0.511), pod length (-0.411), leaf length (-0.393), number of seed per pod (-0.321), days to 80% emergence (-0.298), pod width (0.291) and seed length (-0.221). PC 4 reflected variation more closely linked to yield components and structural traits. Number of pods per plant (0.383), pod width (0.352), stem diameter (0.338), seed breadth (0.288), seed thickness (0.285), number of flower buds per raceme (0.268), leaf length (0.253), days to 80% emergence (-0.216), days to 80% maturity (0.213), thousand seed weight and chlorophyll content at the vegetative stage (-0.209). PC 5 was strongly associated with physiological vigor and plant architecture. Chlorophyll content at the vegetative stage (-0.569), stem diameter (-0.333), pod length (0.321), leaf breadth (-0.264), leaf length (-0.257), days to 80% emergence (-0.234), plant height (0.220), number of seed per pod (0.219), days to 80% maturity (0.215) and days to first flowering (-0.209) were measured.~~ Principal component analysis (PCA) revealed that days to 50% fruiting, days to 50% flowering, thousand seed weight, days to first fruiting, seed breadth, days to first flowering, leaf breadth, leaf length, seed thickness, number of pods per plant, pod length, seed length, chlorophyll content at the vegetative stage and days to 80% maturity were the traits that contributed the most to the overall observed variation.

Table 3. PCA table of 23 quantitative traits of common bean

**Dimensions**	**PC1**	**PC2**	**PC3**	**PC4**	**PC5**
Eigenvalue	6.55	4.09	2.39	1.9	1.36
Variance (%)	28.49	17.77	10.39	8.28	5.93
Cumulative variance (%)	28.49	46.27	56.65	64.93	70.86
**Variables**			**Coefficient vectors**		
Days to 80% germination	0.016	0.182	**-0.298**	**-0.216**	**-0.234**
Chlorophyll content at vegetative stage	-0.035	**0.221**	0.151	**-0.209**	**-0.569**
Leaf length (cm)	0.119	-0.191	**-0.393**	**0.253**	**-0.257**
Leaf breadth (cm)	-0.025	-0.131	**-0.511**	0.188	**-0.264**
Days to first flowering	**-0.329**	0.131	-0.04	-0.01	**-0.209**
Days to 50% flowering	**-0.358**	0.111	-0.028	-0.009	-0.169
Dyas to first fruiting	-0.34	0.157	-0.069	0.072	-0.066
Days to 50% fruiting	**-0.36**	0.135	-0.025	0.009	-0.147
Number of flower bud/raceme	0	**-0.257**	0.127	**0.268**	-0.013
Number of raceme/plant	**-0.265**	-0.028	0.017	-0.027	0.077
Pod length (mm)	-0.205	-0.016	**-0.411**	-0.054	**0.321**
Pod width (mm)	-0.093	0.125	**0.291**	**0.352**	0.036
Plant height (cm)	**-0.273**	0.014	-0.037	0.131	**0.22**
Stem diameter (mm)	-0.003	**-0.256**	0.072	**0.338**	**-0.333**
Number of nodes/plant	**-0.237**	-0.031	0.001	0.129	-0.01
Number of pods/plant	-0.114	-0.179	0.12	**0.383**	-0.08
Number of seed/pod	-0.103	-0.174	**-0.321**	-0.057	**0.219**

Days to 80% maturity	**-0.283**	0.034	0.038	**0.213**	**0.215**
Seed length (mm)	0.188	0.316	**-0.221**	0.109	0.062
Seed breadth (mm)	0.091	**0.405**	-0.056	**0.288**	0.032
Seed thickness (mm)	0.174	**0.352**	-0.016	**0.285**	0.093
Thousand seed weight (g)	0.124	**0.415**	-0.096	**0.211**	0.038
Total yield (t/ha)	**0.24**	-0.137	-0.091	0.204	-0.05

4.4 Hierarchical clustering

The hierarchical cluster dendrogram was constructed by using the 17 quantitative traits across the 58 bean landraces. The optimum number of clusters was selected via the Nbclust package [42]. It evaluates clustering solutions by simultaneously applying 30 different cluster validity indices. The silhouette method classified the quantitative traits into 5 clusters (Figure 4). The average method and Euclidean distance were used to construct the cluster diagram. The average method was utilized, as it has the highest cophenetic correlation value of 0.94 among the methods. Clusters I, II, III, IV and V are represented by red, green, cyan, black and purple colors, respectively (Figure 3). Clusters I, II, III, IV and V consisted of 21, 34, 1, 1 and 1 accessions, respectively. Cluster I contained the most number of accessions 21(~~58.62~~%), whereas clusters III, IV and V contained the lowest number of accessions 1(1.72%) (Table 4).

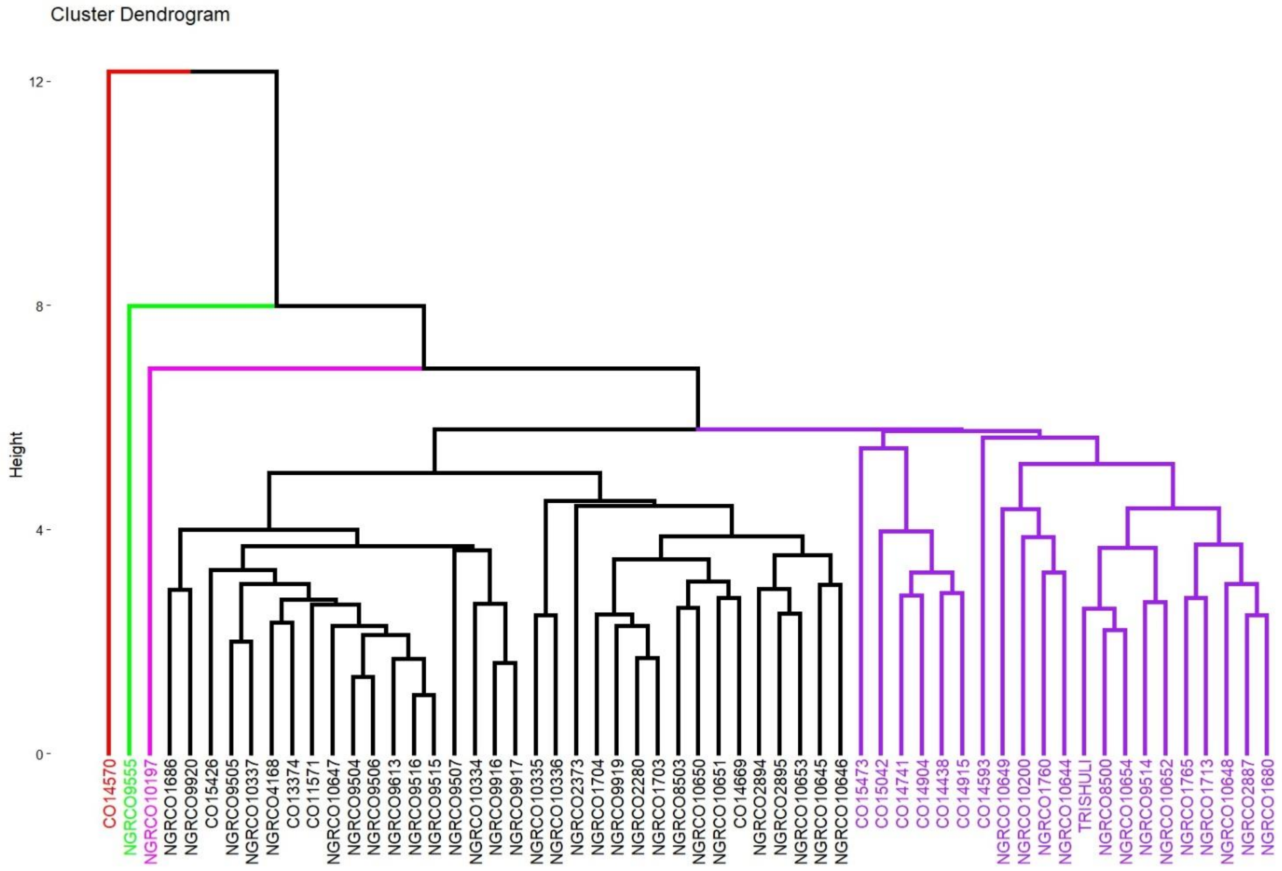

Figure 4. Hierarchical cluster dendrogram with the Euclidean distance and average method

Cluster I showed ~~superior performance, with~~ ~~earlier days to 50% flowering and 50% fruiting at 62.40 and 68.60 days, respectively~~, ~~and a moderate yield (1 t/ha)~~. In Cluster II, the largest cluster was characterized by delayed flowering (79.50

days) and maturity (108 days), the tallest plants (114 cm), and a relatively high chlorophyll content (22.60) but a relatively low yield (0.59 t/ha). Cluster III, represented by a single landrace, exhibited early germination (18 days) but very late maturity (118 days), high pod width (16.30 mm), and a large number of pods per plant (24), resulting in a moderate yield (0.89 t/ha). Cluster IV emerged as the most productive cluster, with the highest yield (2 t/ha), maximum number of pods per plant (27) and number of seeds per pod (7), early maturity (98 days), and relatively large leaf dimensions, with a length of 10.60 cm and breadth of 6.89 cm. In contrast, Cluster V was distinct in terms of exceptionally high chlorophyll content (41.50 ~~CCV~~) and very large seed size, as indicated by the highest seed length (20.40 mm), seed width (13.60 mm), seed thickness (9.06 mm), and thousand seed weight (1236 g) but delayed germination (26 days).

Table 4. Clustering means of the 2 clusters

**Cluster**	**I**	**II**	**III**	**IV**	**V**
Cluster number	21	34	1	1	1
Days to 80% germination	21.10	22.10	18.00	21.00	26.00
Chlorophyll content at vegetative stage (~~CCV~~)	20.20	22.60	25.90	19.60	41.50
Leaf length (cm)	8.30	8.00	6.92	10.60	6.18
Leaf breadth (cm)	5.03	5.27	3.27	6.89	4.31
Days to 50% flowering	62.40	79.50	87.00	70.00	86.00
Days to 50% fruiting	68.60	85.10	87.00	75.00	94.00
Pod length (mm)	8.68	11.30	5.95	9.40	9.08
Pod width (mm)	10.10	9.51	16.30	9.88	10.20
Plant height (cm)	78.30	114.00	101.00	65.50	59.00
Number of pods per plant	9.26	11.40	24.00	27.00	7.00
Number of seed per pod	4.56	5.38	3.00	7.00	4.00
Days to 80% maturity	99.50	108.00	118.00	98.00	100.00
Seed length (mm)	13.40	12.10	11.90	11.60	20.40
Seed width (mm)	7.45	6.64	8.29	7.91	13.60
Seed thickness (mm)	5.84	4.77	5.76	4.69	9.06
Thousand seed weight (g)	371.00	264.00	297.00	293.00	1236.00
Total yield (t/ha)	1.00	0.59	0.89	2.00	0.63

Inter-cluster centroid distance analysis revealed substantial genetic divergence among the five clusters of bean landraces (Table 5). The greatest genetic distance was observed between Cluster II and Cluster V (974.09), followed by the distances between Cluster IV and Cluster V (944.27), Cluster III and Cluster V (940.95), and between Cluster I and Cluster V (866.76), demonstrating that Cluster V is genetically most distinct from the other clusters. In contrast, the lowest inter-cluster distance occurred between Cluster II and Cluster III (41.01), followed by Cluster III and Cluster IV (47.74), indicating close genetic similarity among these clusters. Moderate divergence was recorded among Cluster I with Clusters II, III, and IV.

Table 5. Cluster centroid distance

	Cluster I	Cluster II	Cluster III	Cluster IV	Cluster V
Cluster I	0.00	115.35	87.11	81.60	866.76
Cluster II	115.35	0.00	41.01	61.13	974.09
Cluster III	87.11	41.01	0.00	47.74	940.95
Cluster IV	81.60	61.13	47.74	0.00	944.27

Cluster V	866.76	974.09	940.95	944.27	0.00

4.6 Molecular characterization

The molecular summary statistics are presented in Table 6. The genetic diversity of 58 common bean landraces was assessed via 15 SSR markers. Among the 15 SSR primers, three were monomorphic, whereas the remaining 12 markers were polymorphic and were selected for further analysis. ~~A total of 12 quantitative molecular traits were assessed for molecular diversity~~.

Table 6. Molecular summary statistics of 12 genetic traits among 58 bean landraces

Primers	Base pairs	Total bands	Total alleles	Polymorphic alleles	Allele frequency	Observed heterozygosity (H_o)	Expected Heterozygosity (H_e)	PIC	f	RP	EMR	MI
PV-cct001	100-150	61	3	3	0.776	0.372	0.172	0.34	0.542	1.552	2	0.68
BM143	100-180	61	3	3	0.543	0.511	0.052	0.40	0.901	1.086	2	0.8
BM170	200-500	69	3	3	0.905	0.173	0.190	0.16	-0.087	1.81	2	0.32
BM184	100-160	55	3	3	0.759	0.386	0.000	0.34	1.000	1.518	2	0.68
BM187	180-500	59	4	4	0.569	0.547	0.086	0.46	0.845	1.138	2	0.92
PV-BR35	100-200	55	3	3	0.534	0.540	0.000	0.44	1.000	1.068	2	0.88
PV-BR185	100-150	55	3	3	0.603	0.514	0.000	0.43	1.000	1.206	2	0.86
SSR-IAC10	280-800	86	5	5	0.655	0.505	0.190	0.45	0.630	1.31	2	0.9
SSR-IAC62	100-150	56	3	3	0.603	0.504	0.000	0.41	1.000	1.206	2	0.82
BMd-12	100-150	61	2	3	0.603	0.479	0.034	0.36	0.929	1.206	2	0.72
BM152	100-150	58	3	3	0.690	0.438	0.000	0.36	1.000	1.38	2	0.72
BM210	100-200	55	3	3	0.776	0.361	0.000	0.32	1.000	1.552	2	0.64
Sum		731	40	40						16.032	24	8.94
Mean		60.92	3.16	3.16	0.668	0.444	0.060	0.37	0.866	1.33	2	0.745

Note: PIC= Polymorphic information content, EMR= Effective multiplex ratio, MI= Marker index and RP= Resolving power, f= Fixation index

The size of the band's ranges from 100-800 base pairs. The total number of bands was 697, with an average of 60.92 bands for each marker. The highest number of bands produced by the BM170 primer was 69, and the lowest was produced by BM184, PV-BR35, PV-BR185 and BM210, with 55 bands. The total number of alleles was 40, with an average of 3.16 alleles per marker. Similarly, the total number of polymorphic alleles was 40, with an average of 3.16. [22] used 25 SSR primers and reported an average of 4.20 alleles per primer, which is slightly greater than our findings. Our findings are in line with those of [14], who used 27 SSR primers and likewise reported 2-12 alleles per marker. The allele frequency distribution spans from 0.534-0.905, which is a medium-high frequency distribution, which aligns with the findings of [14], who reported a range of 0.20-0.79, and [32] reported a range from 0.27-0.78. The allele frequency distribution generally ranges from 0-1.

The observed heterozygosity (H_o) ranged from 0.173 (BM170) to 0.547 (BM187), with an overall mean of 0.444, indicates a moderate level of heterozygosity within the germplasm. This pattern suggests differential allelic expression across loci, consistent with the presence of both fixed and segregating alleles. Comparable studies have reported H_o values ranging from 0.62 [61], highlights slightly higher heterozygosity in other bean populations. The expected heterozygosity (H_e) ranged from 0 to 0.19, with an average of 0.06, reflected the predominance of alleles with low frequency across the SSR loci. Certain loci, such as SSR-IAC10, exhibited relatively higher H_e (0.19), contributing disproportionately to the overall genetic diversity of the population. Polymorphic information content (PIC), an indicator of the discriminatory capacity of each SSR marker, ranged from 0.16 (BM170) to 0.46 (BM187), with a mean of 0.37. Markers with higher PIC values, including BM187 and SSR-IAC10, were particularly effective in revealing genetic variation among landraces. These findings are comparable with [62], who reported PIC values of 0.33-0.66 in bean germplasm, whereas populations with fewer alleles, such as reported by [31], exhibited lower PIC values (0.17-0.41).

The resolving power (RP) of SSR markers, reflecting their ability to discriminate among accessions, varied between 0.542 and 1.0, with an average of 0.866. This indicates a moderate to high discriminative capacity across loci. The effective multiplex ratio (EMR) was consistently 2 for all markers, suggesting uniform amplification efficiency. The marker index (MI), a composite measure of marker informativeness integrating RP and EMR, ranged from 0.32 (BM170) to 0.92 (BM187), with an average of 0.745. Higher MI values correspond to more informative markers, indicating their suitability for genetic differentiation and diversity studies. These results are in accordance with [63], who reported RP values of 0.45-0.89 and MI values of 0.39-1.28 for SSR markers in legumes.

4.7 Phylogenetic tree

The molecular phylogenetic tree was constructed via the unweighted pair group method with arithmetic average (UPGMA) method and Neis's (1983) genetic distance in NTsyspc software. Using 12 SSR markers, the 58 bean landraces were classified into 3 major clusters (Figure 5). Similarly, [14] and [63] also reported the major molecular cluster of 2 when 27 and 23 SSR primers were used. Cluster-1, cluster-2 and cluster-3 contained 12, 36 and 10 accessions, respectively.

Cluster I consisted of 12 accessions, predominantly originating from the High hills (>1500 m) region, including accessions from Khotang, Dhankhuta, Panchthar, Sankhuwasabha, and Lamjung. The clustering indicates that accessions from the same district (e.g., NGRCO9504 and NGRCO9505 from Khotang; NGRCO9515 and NGRCO9516 from Dhankhuta) were grouped together, showed shared genetic backgrounds and potential local adaptation. Cluster II was the largest,

containing 36 accessions, and included a mix of Mid-hills (500-1500 m) and High hills (>1500 m) accessions. Accessions originated from a broad range of districts. Among the 36 accessions, approximately 20 were from high-hill regions and 16 from mid-hill regions, highlighting the relatively broad genetic base within this cluster. Notably, accessions from the same district were generally clustered together ~~(e.g., NGRCO10197 and NGRCO10200 from Jumla; NGRCO1703 and NGRCO1704 from Bajura)~~, but some districts exhibited distribution across subclusters. Cluster III comprised 10 accessions, with a dominance of High hills (>1500 m) origin. The cluster included accessions from Myagdi, Mustang, Panchthar, Dolakha, and the variety Trishuli. ~~Most accessions from the same district were grouped together (e.g., NGRCO2373 and NGRCO2284 from Mustang; CO14669, Trishuli, and NGRCO8500 from Dolakha)~~. Overall, the dendrogram illustrates that geographic origin, particularly altitude, plays a significant role in the genetic structure of Nepalese bean landraces. Accessions from high-altitude districts tend to cluster together, whereas mid-hill and lowland.

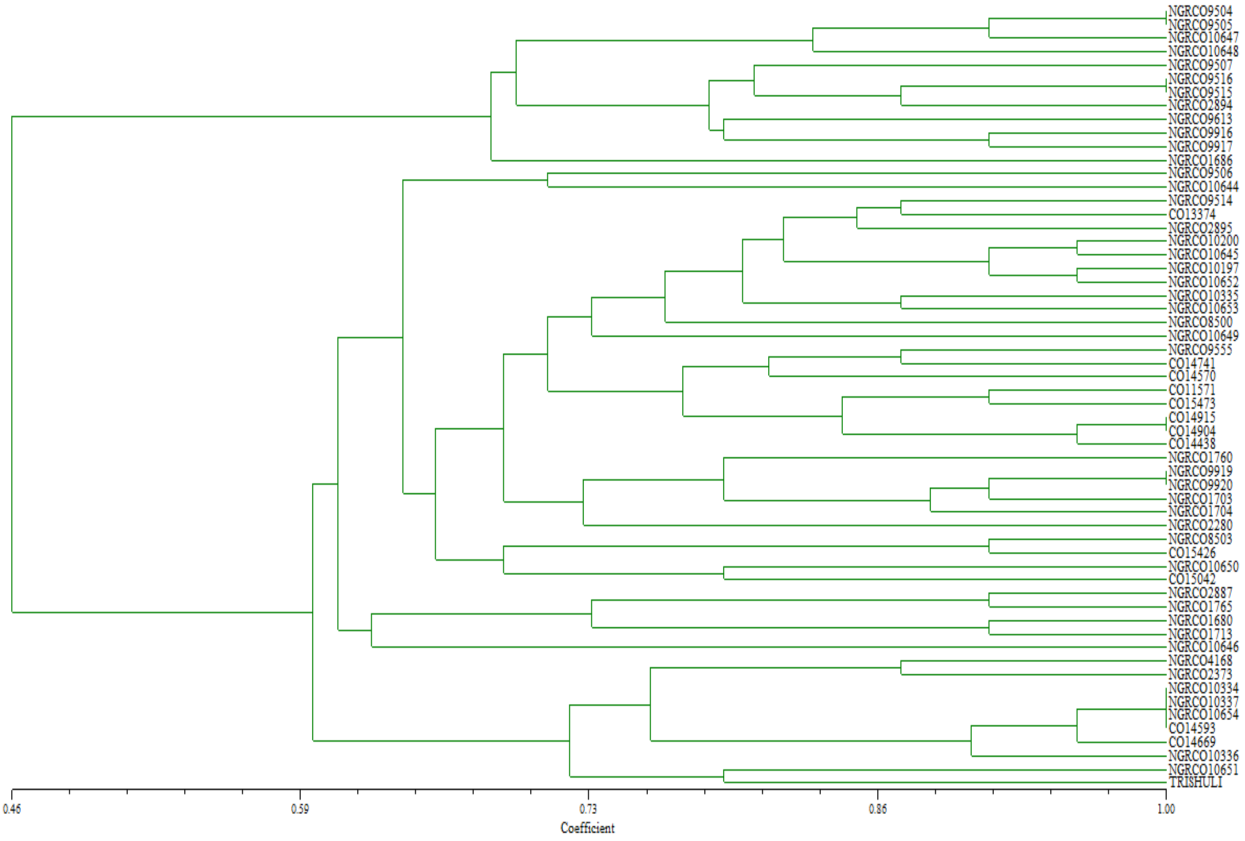

Figure 5. Molecular cluster dendrogram of 12 SSR markers among 58 SSR landraces

The UPGMA dendrogram did not reveal distinct clustering of the common bean accessions according to their collection sites, indicating a lack of clear geographic structuring. This finding was further supported by the results of [22, 14] and [64], who reported that there was no significant correlation between geographical origin and genetic similarity in their cluster analysis of bean landraces. This lack of clear grouping suggests that beans from different regions are genetically similar and that such genetic similarity is likely due to high gene flow. In contrast, SSR-based UPGMA analysis by [1] revealed geographic separation among bean genotypes from Ordu, likely due to traditional local seed production and limited external seed exchange.

4.8 Principal coordinate analysis

Principal coordinate analysis (PCoA) is an ordination method that transforms a similarity or dissimilarity matrix into a low-dimensional plot, aiming to preserve the original distances between individuals [65]. It is reliable when the first axes explain ≥25% of the variation. The PCoA identified three main axes that describe genetic differences among the bean accessions. Axis 1 explained 27.44% of the total variation, indicating that it represented the largest share of genetic diversity. Axis 2 adds another 15.83%, whereas Axis 3 accounts for 9.87%. Together, the first two axes capture approximately 43.27% of the total variation, offering a good two-dimensional view of the genetic patterns (Table 7; Figure 6). When the third axis is included, the cumulative explained variation increases to 53.14%, meaning that these axes together describe most of the genetic differences in the population.

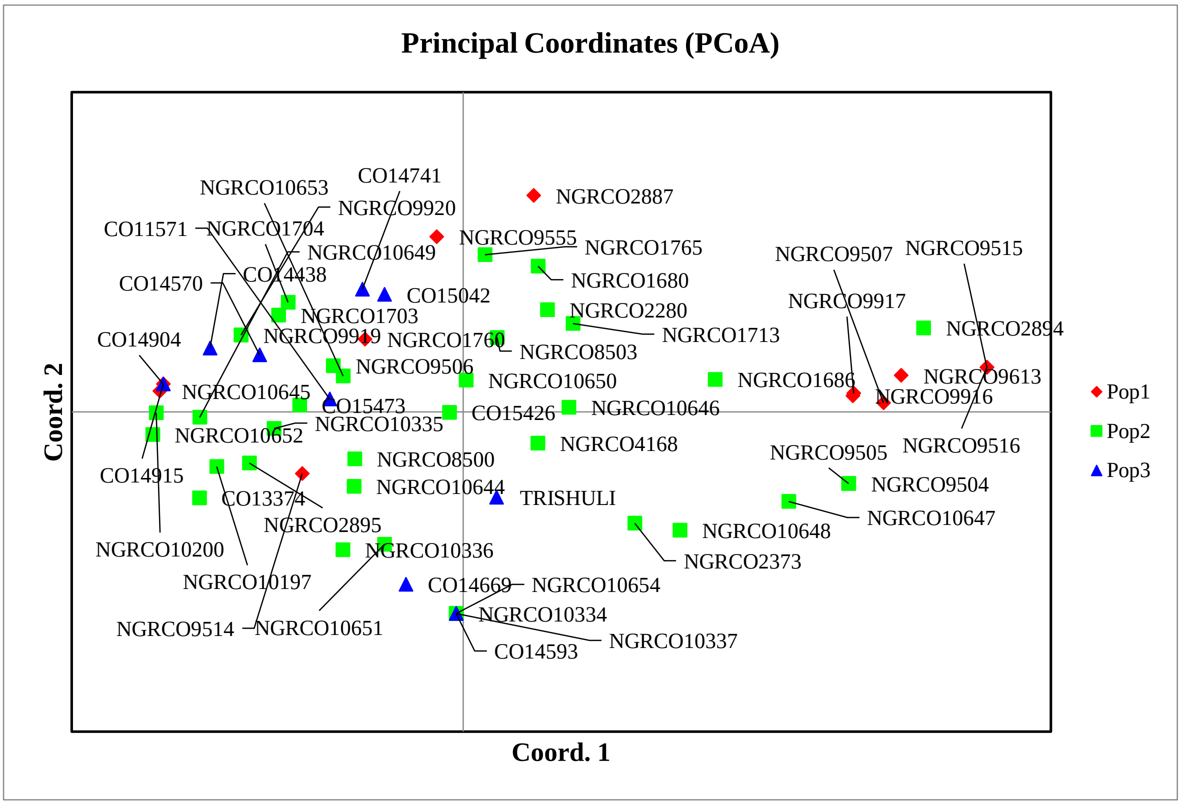

Figure 6. Molecular principal coordinate analysis showing three axis among three populations

Bean landrace accessions were grouped into three populations based on collection altitude representing agro-ecological regions of Nepal: Population 1 (Terai, <500 m) with 6 accessions, Population 2 (Mid-hills, 500-1500 m) with 18 accessions, and Population 3 (High hills, >1500 m) with 34 accessions. These populations were used for principal coordinate analysis (PCoA) based on SSR markers. Unlike the clear separation observed in the cluster dendrogram, the PCoA plot reveals that accessions from different clusters are not strictly grouped together. Instead, there was noticeable overlap and intermixing among accessions belonging to Pop1, Pop2, and Pop3. While some accessions from the same cluster are positioned in proximity, others are dispersed and intermixed with accessions from different clusters. ~~Similar kinds of PCoA patterns were found in the SSR based characterization of bean landraces by [66], [22] and [67] who reported intermixing of the landraces among populations~~.

Table 7. Principal coordinate analysis of 58 bean landraces for 3 axis

**Axis**	**1**	**2**	**3**
Percentage (%)	27.44	15.83	9.87
Cumulative percentage (%)	27.44	43.27	53.14

4.8 Analysis of the molecular variance (AMOVA)

The analysis of molecular variance (AMOVA) was conducted via a Neis distance matrix of 58 accessions on the basis of the three molecular clusters. The AMOVA revealed that 93% of the total genetic variation resided within populations, whereas 7% was attributed to differences among populations (Table 8). This distribution indicates that individual accessions within each population harbor substantial genetic diversity. The AMOVA yielded a PhiPT value of 0.344 (P = 0.001), indicating moderate and statistically significant genetic differentiation among the populations. This result suggests that approximately 7% of the total genetic variation is attributable to differences between populations, whereas the remaining variation occurs within populations. S~~imilarly, [61] reported an intrapopulation variance of 83% and a variance of 17%, and [14] reported an intra-population variance of 66% and an interpopulation variance of 34%.~~

Table 8. Analysis of molecular variance (AMOVA) for 3 populations

Source	df	SS	MS	Est. Var.	%	PhiPT	P value
Among Pops	2	43.923	21.962	0.781	7%	0.075	0.005
Within Pops	55	532.611	9.684	9.684	93%		
Total	57	576.534		10.465	100%		

Note: df= degree of freedom, SS= sum of squares, Est. Var= Estimated variance and %= Percentage of total variance

4.10 Population structure analysis

The population structure of the 58 bean landraces based on 12 SSR markers revealed the presence of three major genetic clusters (K = 3), indicating the underlying genetic composition of the studied accessions. The STRUCTURE bar plot (Figure 7) illustrates the proportional membership coefficient (Q) of each accession in the inferred clusters, where each vertical bar represents an individual accession and the colors blue, green, and red represent the three inferred genetic groups. Based on the highest membership coefficient value, out of the 58 accessions, 26 accessions were assigned to subpopulation I, 11 accessions to subpopulation II, and 21 accessions to subpopulation III (Table 10). The STRUCTURE bar plot indicated that several accessions exhibited admixed ancestries, shown by multiple colors within a single bar, suggesting shared genetic components among clusters. However, some accessions showed predominant membership in a single cluster with high membership coefficients (Q values), indicating relatively distinct genetic backgrounds with limited admixture.

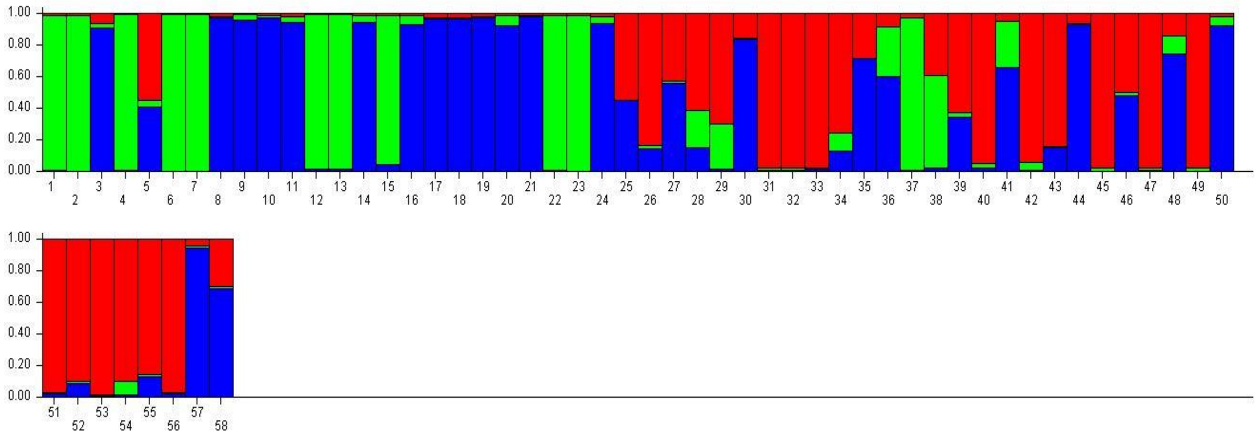

Figure 7. Population structure analysis of 12 SSR markers of 58 bean landraces

Genetic diversity parameters estimated for the three inferred subpopulations indicated moderate variation in expected heterozygosity (H_e) among the clusters (Table 9). The expected heterozygosity ranged from 0.272 to 0.374, with a mean value of 0.312, showed a moderate level of genetic diversity within the inferred populations. Among the clusters, subpopulation 3 exhibited the highest genetic diversity (H_e = 0.374), whereas subpopulation 1 showed the lowest diversity (H_e = 0.272). F_{st} serves as a measure of genetic divergence between different subgroups within a population [63]. The fixation index (F_{st}) ranged from 0.224 to 0.505, with an average value of 0.406. The highest genetic differentiation was observed in subpopulation 2 (F_{st} = 0.505), followed by subpopulation 1 (F_{st} = 0.488), indicate strong genetic differentiation from the overall population. In contrast, subpopulation 3 exhibited comparatively lower differentiation (F_{st} = 0.224), suggest relatively higher genetic exchange or shared ancestry with other groups. These results indicate that while some level of admixture exists among the bean landraces, the identified clusters still maintain considerable genetic differentiation.

Table 9. Expected heterozygosity and F_{st} among the 4 subpopulations

Subpopulation (K)	Expected heterozygosity (H_0)	F_{st}
1	0.272	0.488
2	0.289	0.505
3	0.374	0.224
Mean	0.312	0.406

Table 10. Membership coefficient of bean genotypes in three subpopulations

	**Subpopulation**		
Accessions	**I**	**II**	**III**
NGRCO9504	0.011	0.981*	0.008
NGRCO9505	0.012	0.981*	0.007
NGRCO9506	0.061	0.031	0.908*
NGRCO9507	0.007	0.983*	0.01
NGRCO9514	0.543*	0.046	0.412
NGRCO9516	0.004	0.991*	0.005
NGRCO9515	0.004	0.991*	0.005
NGRCO1760	0.015	0.007	0.977*
NGRCO2887	0.006	0.032	0.961*
NGRCO9555	0.012	0.011	0.977*
NGRCO1765	0.018	0.038	0.944*
NGRCO2894	0.005	0.979*	0.016
NGRCO9613	0.007	0.975*	0.018
NGRCO1680	0.008	0.048	0.944*
NGRCO1686	0.011	0.945*	0.044
NGRCO2280	0.008	0.063	0.929*
NGRCO9919	0.028	0.006	0.967*
NGRCO9920	0.024	0.007	0.969*
NGRCO1703	0.019	0.006	0.976*
NGRCO8503	0.014	0.063	0.923*
NGRCO1704	0.012	0.005	0.983*
NGRCO9916	0.011	0.977*	0.011

NGRCO9917	0.008	0.985*	0.007
NGRCO1713	0.018	0.041	0.941*
NGRCO10200	0.544*	0.005	0.451
NGRCO10197	0.829*	0.026	0.145
NGRCO2895	0.428	0.011	0.561*
NGRCO4168	0.612*	0.238	0.15
NGRCO2373	0.698*	0.287	0.015
NGRCO10335	0.153*	0.007	0.84
NGRCO10334	0.975*	0.017	0.008
NGRCO10337	0.978*	0.014	0.008
NGRCO10336	0.972*	0.01	0.017
NGRCO10644	0.754*	0.113	0.132
NGRCO10645	0.279	0.005	0.716*
NGRCO10646	0.085	0.314	0.601*
NGRCO10647	0.022	0.966*	0.012
NGRCO10648	0.391	0.581*	0.028
NGRCO8500	0.627*	0.029	0.344
NGRCO10649	0.948*	0.029	0.023
NGRCO10650	0.046	0.295	0.659*
NGRCO10651	0.942*	0.049	0.008
NGRCO10652	0.839*	0.005	0.157
NGRCO10653	0.059	0.01	0.931*
NGRCO10654	0.977*	0.016	0.007
CO11571	0.498*	0.019	0.483
CO14669	0.977*	0.013	0.01
CO15426	0.141	0.113	0.746*
CO14593	0.975*	0.018	0.008
CO15042	0.016	0.056	0.928*
CO14915	0.97*	0.005	0.025
CO15473	0.894*	0.02	0.086
CO13374	0.982*	0.005	0.013
TRISHULI	0.894*	0.086	0.02
CO14438	0.855*	0.012	0.133
CO14904	0.969*	0.005	0.027
CO14741	0.041	0.014	0.945*
CO14570	0.298	0.01	0.692*

5. Discussion

Landraces are vital for maintaining agrobiodiversity but are increasingly threatened by genetic erosion due to habitat loss, replacement by uniform elite varieties, urbanization, deforestation, overexploitation, and invasive alien species, leading to a reduction in overall genetic diversity [68]. Therefore, it is essential to prioritize the morphological and molecular characterization of landraces, along with conservation-oriented breeding strategies using morphological and molecular markers and ex situ preservation through gene banks.

The common bean landraces exhibited considerable phenotypic and genotypic variation. The days to first flowering ranged from 50-83 days, and the days to first fruiting ranged from 53-85 days. Accessions from hilly regions generally

require a longer duration to initiate flowering than those from warmer Terai plains do [3, 57]. The accession NGRCO9507 from Tanahun flowered earliest (50 days), whereas NGRCO9555 from Rupandehi exhibited the latest flowering. The observed variation in flowering and fruiting times is likely influenced by environmental factors, particularly temperature, which regulate physiological and phenological development [69]. Early-flowering and early-fruiting accessions can be utilized in breeding programs aimed at developing short-duration and stress-escaping cultivars, whereas late-maturing accessions may serve as valuable parents for improving adaptation and yield stability in favorable agroecological environments. The accessions showed wide variation in maturity duration, reaching physiological maturity between 85 and 123 days. It is a key agronomic trait influencing farmers' varietal preference, as early-maturing cultivars reduce exposure to biotic stresses and enable earlier market entry and crop intensification [64, 70]. The accession NGRCO4168, a bush-type accession, matured earliest (85 days), whereas NGRCO9514, an indeterminate and moderately climbing accession, exhibited the latest maturity (123 days). This variation is likely associated with differences in growth habits and temperature. The indeterminate genotypes maintaining prolonged vegetative growth that delays reproductive development [71], and with low-temperature stress in high-altitude environments extending crop phenology [56]. The common bean accessions substantial variation in total yield, ranging from 0.01 to 2.01 t ha^{-1}, with accession NGRCO9516 resulting in the highest yield (2.01 t ha^{-1}). The superior performance of this accession is likely associated with its determinate growth habit and relatively short maturity duration (87 days), which favor synchronized flowering and pod set. These phenological attributes may increase yield components, including pod and seed numbers per plant, by enabling partial escape from abiotic stresses commonly affecting the flowering and pod development stages [72]. Pod length exhibited substantial variation among the accessions, ranging from 5.95 to 15.13 mm. The maximum pod length (15.13 mm) was recorded for the check variety NGRCO8500, followed closely by accession NGRCO1765 (15.01 mm). Pod length is a key economically important trait, as it directly influences yield potential and plays a significant role in marketability and consumer preference [22, 73].

Among the 58 landraces studied, the flower color was predominantly lilac, followed by white, purple, and dark lilac with purplish spots. Flower color in common bean is regulated primarily by the V locus, where the dominant V allele promotes anthocyanin production, resulting in purple/pink flowers, and the recessive v allele produces white flowers [74]. Seed coat color is controlled mainly by loci such as P, V, and C, which regulate anthocyanin and flavonoid biosynthesis, with silencing of the P gene during domestication contributing to white-seeded phenotypes [74, 75]. The color of the seed coat is mostly black and brown, which is characteristic of the Mesoamerican gene pool, whereas maroon, red, pink, purple, pale cream, and yellow-green hues strongly represent Andean types with diverse pigmentation patterns [21, 76, 77]. The presence of dual pigmentation and lighter seed colors suggests a mixed gene pool composition. Colored seeds, particularly black and red seeds, are often associated with relatively high antioxidant contents due to the presence of anthocyanins, making them valuable for nutritional enhancement [78]. Conversely, white-seeded varieties are preferred in certain markets for their aesthetic appeal and faster cooking times [79]. Seed traits are the primary characteristics of beans that influence both their productivity and economic value [3]. Morphologically, 27% of bean accessions of Andean origin had more than 40 g of seed/100 g of seed weight, whereas the remaining 72.41% were of Mesoamerican origin and had less than 25 g of seed/100 g of seed weight. This diversity in seed color observed among the studied common bean accessions reflects potential differences between the Andean and Mesoamerican gene pools. Seed shape can also be classified into 5 major shapes and is dominated by cuboid seed shape. The observed variation in the seed coat pattern, including mottled patterns, aligns with [80], who noted that complex patterns arise from interactions between multiple loci. Common bean

seed characteristics such as color, size, and shape play key roles in consumer selection, as individual preferences vary from person to person [22]. All of these types of seed-related diversity, including color and shape, might be due to hybridization between the gene pools, intergene pool introgression, recombination, mass selection, seed sale and exchange, selection for cooking traits such as taste, and minimum selection by farmers [81, 82, 83, 84]. Considerable variation in plant growth type was observed across the accessions, which were classified into six unique categories. This indeterminate growth habit type prevalence is most likely a result of both ecological adaptation and the cropping strategy used in the region [59]. According to [57], this indeterminate semi-climber growth habit is most suited for high altitudes in the Himalayan area, where temperatures are lower than those in the lowland regions of Nepal. Beans are typically planted as companion crops with maize and amaranth in hilly areas, where climbing varieties are more attractive, whereas in plains, beans are typically cultivated as sole crop-bush types. Those that exhibited determinate growth habit and a shorter maturity time produced more pods and seeds per plant because these accessions were able to avoid the adverse circumstances of high daily temperatures and low humidity during the blooming and pod formation phases [72].

Principal component analysis (PCA) is a multivariate statistical method for simplifying complex datasets by identifying key traits, uncovering their interrelationships, key roles, and the nature of traits, which is crucial for recognizing the diversity within breeding material, identifying traits that contribute to successful growth and eliminating low breeding value traits [56]. The first six components captured 70.86% of the total variation, with traits such as days to 50% fruiting, days to 50% flowering, thousand seed weight, days to first fruiting, seed breadth, days to first flowering, leaf breadth, leaf length, seed thickness, number of pods per plant, pod length, seed length, chlorophyll content at the vegetative stage and days to 80% maturity contributing the most to the observed variation. The distinct separation of accessions in the biplot suggests the potential to cross genetically and phenotypically divergent lines to create new varieties with the desired combinations for early flowering, fruiting, yield and growth habit. Breeders can use this information to select parent lines that combine complementary vegetative and reproductive traits. Clustering a large number of germplasm accessions into a homogeneous group facilitates the selection of diverse parents, enables precise comparisons among populations, and increases the likelihood of combining desirable genes for superior progeny development [85]. In the present study, the landraces were grouped into five distinct clusters. Cluster V, represented by accession NGRCO14570, was characterized by superior seed length, breadth, and thickness and high seed weight, reflecting traits typical of the Mesoamerican gene pool and offering the potential to improve the size of seeds preferred by farming communities. The cluster IV accession NGRC10197 presented high yield potential, early maturity, a determinate growth habit, and relatively high numbers of pods and seeds per plant, making it a promising parent for yield improvement. The cluster I accession NGRCO4168 exhibited early 50% flowering and fruiting, indicating its suitability for developing early-maturing cultivars. The large inter-cluster distances among Clusters I, IV, and V suggest substantial genetic divergence, supporting the strategic use of NGRC10197 and NGRCO4168 in hybridization to combine early maturity with high yield, followed by introgression of seed size traits from NGRCO14570. The lack of correspondence between geographical origin and morphological clustering indicates that phenotypic diversity is shaped more by genetic drift, selection pressure, environmental effects, and seed exchange than by collection site [86, 87].

The SSR primers amplified a wide range of fragments, ranging from 100-800 base pairs. This type of variability is characteristic of SSR markers, where repeat motif expansions or contractions generate different allele sizes [88]. Larger ranges may indicate higher mutability, whereas narrower ranges suggest stable motifs with lower mutation rates [89].

Differences in repeat motifs (di- versus tri-nucleotide) and flanking sequence lengths contribute to the observed fragment range [82]. The observed band size facilitates accurate allele scoring, which is crucial for distinguishing closely related landraces [90]. The total number of amplified bands per primer ranged from 55-69, indicating great diversity. Higher band counts often correlate with robust PCR amplification, whereas lower counts may indicate null alleles or amplification bias [47]. Heterozygous individuals contribute two bands per locus, whereas homozygous individuals contribute one, so higher band counts are associated with greater heterozygosity [91]. The total number of alleles ranged from 3-5. Variation in allele counts arises from the use of different markers and sample sizes collected from various locations [29]. SSRs typically exhibit high allelic richness in cross-pollinated species; however, in predominantly selfing species such as common bean, allele numbers are constrained by the self-pollinating reproductive system, which promotes homozygosity and reduces allelic richness through inbreeding [92]. In outcrossing species such as maize, where SSRs often exhibit >5 alleles per locus due to relatively high recombination rates and gene flow [93]. The observed heterozygosity (H_o) exceeded the expected heterozygosity (H_e) across most loci, reflecting an excess of heterozygotes in the studied Nepalese bean landraces. Common bean is predominantly self-pollinated, which reduces H_0 via limited outcrossing and increases homozygosity [94]. The (H_0>H_e) for a self-pollinating species may result from occasional outcrossing events, farmer-mediated seed exchange, mixed cropping systems, or admixture between distinct populations, which maintain heterozygosity despite the predominantly self-pollinating nature of common bean [95, 96]. The PIC reflects marker informativeness, with values >0.5 considered highly discriminatory [97]. The low to medium mean PIC aligns with the limited allele number (3-5), as the PIC is influenced by both allele count and frequency distribution [98]. Higher PIC values indicate greater utility for genetic mapping and diversity studies [97, 98]. Low PIC values are typically found among closely related genotypes, and high values are typically found among more genetically diverse genotypes, such as cross-pollinated species [99].

Principal coordinate analysis (PCoA) facilitates the examination of population relationships by converting genetic distances into spatial coordinates [100]. There was no correlation between PCoA grouping and cluster analysis in this study. These results suggest low genetic differentiation among common bean accessions from different regions, likely due to extensive gene flow by farmer-to-farmer seed exchange across districts, which is a dominant practice in Nepal. This exchange system promotes the spread of genetic material across wide areas, leading to homogenization of genetic diversity. The analysis of molecular variance (AMOVA) revealed greater variation within populations than between populations, which also verified the PCoA results. The population structure analysis identified 3 clusters, which also showed a wide range of admixture and shared ancestry among the populations. The high F_{st} values suggest substantial genetic differentiation among the subpopulations, supporting the presence of well-structured genetic variation within the studied bean landraces. The observed population structure, together with moderate heterozygosity and noticeable interpopulation differentiation, suggests that the genetic diversity within the studied common bean accessions is influenced by both intrapopulation variation and limited gene flow between subpopulations. The genetic variation revealed through PCoA, AMOVA and population structure analysis can largely be attributed to a combination of a range of evolutionary and ecological mechanisms among plant varieties. These include both artificial and natural selection, gene flow between populations, and random processes such as genetic drift.

Conclusion

The majority of qualitative, quantitative, and molecular traits evaluated in Nepalese common bean landraces revealed substantial phenotypic and genotypic diversity. Agro-morphological assessment revealed that many landraces outperformed the check varieties in terms of key reproductive and yield-related traits. Farmers in Nepal prefer genotypes with early fruiting and maturity, climbing growth habits, high yields, and more seeds per pod. The identified early-fruiting and early-maturing bush-type landraces can serve as valuable parents in hybridization with high-yielding climbing types to combine earliness and productivity. SSR-based molecular characterization confirmed the morphological variability, revealing moderate to high genetic diversity, high heterozygosity and allelic diversity. Most variation occurred within populations, and weak geographic structuring with substantial admixture indicated extensive gene flow among landraces. While SSR markers can be used to assess diversity and population structure effectively, future studies using high-density SNP genotyping and GWAS could provide a finer resolution of trait–marker relationships, which will support marker-assisted selection and the development of improved, farmer-preferring bean varieties adapted to diverse agro-ecological regions of Nepal.

Acknowledgements

We would like to thank the Nepal Agricultural Research Council, Government of Nepal, for providing all support for conducting the research. The team of the National Agriculture Genetic Resources Center, Khumaltar, is duly acknowledged for their valuable contribution to carrying out this experiment.

References:

AI, A., Ojobo, O.A. Genetic diversity assessment of Phaseolus vulgaris L. landraces in Nigeria's mid-altitude agroecological zone. Int. J. Biodivers. Conserv. 2012;4(13): 453–460. https://doi.org/10.5897/IJBC11.21

Anderson, J.A., Churchill, G.A., Autrique, J.E., Tanksley, S.D., Sorrells, M.E. Optimizing parental selection for genetic linkage maps. Genome.1993; 36(1): 181–186. https://doi.org/10.1139/g93-024

Angioi, S.A., Rau, D., Attene, G., Nanni, L., Bellucci, E., Logozzo, G., et al. Beans in Europe: origin and structure of the European landraces of Phaseolus vulgaris L. Theor. Appl. Genet. 2010; 121(5): 829–843. https://doi.org/10.1007/s00122-010-1353-2

Arunga, E.E., Kinyua, M., Ochuodho, J., Owuoche, J., Chepkoech, E. Genetic diversity of determinate French beans grown in Kenya based on morpho-agronomic and simple sequence repeat variation. J. Plant Breed. Crop Sci. 2015; 7(8): 240–250. https://doi.org/10.5897/JPBCS2015.0503

Aryal, K., Poudel, S., Chaudhary, P., Chaudhary, R.P., Ghimire, K.H., Shrestha, D.S., Joshi, B.K.. Agro-morphological diversity of high altitude bean landraces in the Kailash Sacred Landscape of Nepal. J. Nepal Agric. Res. Counc. 2020; 6: 1–13. https://doi.org/10.3126/jnarc.v6i0.28109

Auguie, B., Antonov, A., Auguie, M.B. gridExtra: miscellaneous functions for "grid" graphics [software]. CRAN; 2017. https://doi.org/10.32614/CRAN.package.gridExtra

Beebe, S., Skroch, P.W., Tohme, J., Duque, M.C., Pedraza, F., Nienhuis, J. Structure of genetic diversity among common bean landraces of Middle American origin based on correspondence analysis of RAPD. Crop Sci. 2000; 40(1): 264–273. https://doi.org/10.2135/cropsci2000.401264x

Blair, M.W., Díaz, L.M., Buendía, H.F., Duque, M.C.. Genetic diversity, seed size associations and population structure of a core collection of common beans (Phaseolus vulgaris L.). Theor. Appl. Genet. 2009; 119(6): 955–972. https://doi.org/10.1007/s00122-009-1064-8

Blair, M.W., Giraldo, M.C., Buendia, H.F., Tovar, E., Duque, M.C., Beebe, S.E. Microsatellite marker diversity in common bean (Phaseolus vulgaris L.). Theor. Appl. Genet. 2006; 113: 100–109. https://doi.org/10.1007/s00122-006-0276-4

Blair, M.W., González, L.F., Kimani, P.M., Butare, L. Genetic diversity, inter-gene pool introgression and nutritional quality of common beans (Phaseolus vulgaris L.) from Central Africa. Theor. Appl. Genet. 2010; 121(2): 237–248. https://doi.org/10.1007/s00122-010-1305-x

Botstein, D., White, R.L., Skolnick, M., Davis, R.W. Construction of a genetic linkage map in man using restriction fragment length polymorphisms. Am. J. Hum. Genet. 1980; 32(3): 314–331.

Charrad, M., Ghazzali, N., Boiteau, V., Niknafs, A. NbClust: an R package for determining the relevant number of clusters in a data set. J. Stat. Softw. 2014; 61: 1–36. https://doi.org/10.18637/jss.v061.i06

Choudhary, K., Mathur, N., Choudhary, O.P., Pillai, U. Protocol for isolation of genomic DNA from dry and fresh leaves of Vigna species suitable for RAPD and restriction digestion. Adv. Biol. Res. 2008; 2(5–6): 83–89.

Comtois, D. summarytools: tools to quickly and neatly summarize data [software]. CRAN; 2014. https://doi.org/10.32614/CRAN.package.summarytools

Darai, R., Sah, R., Sah, J. B., Gupta, B., & Rasaili, S. Assessment of quantitative traits of rajma bean (*Phaseolus vulgaris* L.) accessions for yield and yield-attributing traits in multi-environments of Nepal. Annals of Agricultural & Crop Sciences. (2021); *6*(2): 1075. https://doi.org/10.26420/annagriccropsci.2021.1075

Dawo, M.I., Sanders, F.E., Pilbeam, D.J. Yield, yield components and plant architecture in the F3 generation of common bean (Phaseolus vulgaris L.) derived from a cross between the determinate cultivar 'Prelude' and an indeterminate landrace. Euphytica. 2007; 156(1): 77–87. https://doi.org/10.1007/s10681-007-9354-1

Dennis, O., Phinehas, T., James, K., Annet, N., Pamela, P., Michael, U., Paul, G. The genetic diversity and population structure of common bean (Phaseolus vulgaris L.) germplasm in Uganda. Afr. J. Biotechnol. 2014; 13(29): 2935–2949. https://doi.org/10.5897/ajb2014.13916

Doyle, J.J., Doyle, J.L. A rapid DNA isolation procedure for small quantities of fresh leaf tissue. Phytochem. Bull. 1987; 19: 11–15.

Duke, J.Handbook of legumes of world economic importance. New York: Springer Science & Business Media; 2012.

Ekbic, E., Hasancaoğlu, E.M. Morphological and molecular characterization of local common bean (Phaseolus vulgaris L.) genotypes. Appl. Ecol. Environ Res. 2019; 17(1): 841–853. https://doi.org/10.15666/aeer/1701_841853

Ellegren, H. Microsatellites: simple sequences with complex evolution. Nat Rev Genet. , 2004; 5(6): 435–445. https://doi.org/10.1038/nrg1348

Enyew, M., Feyissa, T., Carlsson, A.S., Tesfaye, K., Hammenhag, C., Geleta, M. Genetic diversity and population structure of sorghum [Sorghum bicolor (L.) Moench] accessions as revealed by single nucleotide polymorphism markers. Front. Plant Sci. 2022; 12: 799482. https://doi.org/10.3389/fpls.2021.799482

Eticha F, Bekele E, Belay G, Börner A. Phenotypic diversity in tetraploid wheats collected from Bale and Wello regions of Ethiopia. Plant Genet Resour. 2005; 3(1):35–43. https://doi.org/10.1079/PGR200457

Evanno, G., Regnaut, S., Goudet, J. Detecting the number of clusters of individuals using the software STRUCTURE: a simulation study. Mol. Ecol. 2005; 14(8): 2611–2620. https://doi.org/10.1111/j.1365-294X.2005.02553.x

FAOSTAT. FAOSTAT database. 2023 .Available from http://www.fao.org/faostat/en/#home (accessed 18 February 2024).

Fissehaa, Z.. Phenotypic diversity and variability of common bean (Phaseolus vulgaris L.) landrace germplasm from Ethiopia. J. Pastoralism. 2020; 1(2): 20–29.

Galili, T. dendextend: an R package for visualizing, adjusting and comparing trees of hierarchical clustering. Bioinformatics. 2015; 31(22): 3718–3720. https://doi.org/10.1093/bioinformatics/btv428

Genebank, 2023. Annual Report (2022/23). Lalitpur, Nepal: National Agriculture Genetic Resources Centre, NARC; 2023

Gepts, P., Osborn, T.C., Rashka, K., Bliss, F.A.. Phaseolin-protein variability in wild forms and landraces of the common bean (Phaseolus vulgaris): evidence for multiple centers of domestication. Econ. Bot. , 1986; 40: 451–468. https://doi.org/10.1007/BF02859659

Gioia, T., Logozzo, G., Marzario, S., Spagnoletti Zeuli, P., Gepts, P. Evolution of SSR diversity from wild types to US advanced cultivars in the Andean and Mesoamerican domestications of common bean (Phaseolus vulgaris). PLoS ONE. 2019; 14(1): e0211342. https://doi.org/10.1371/journal.pone.0211342

Godfray, H.C.J., Beddington, J.R., Crute, I.R., Haddad, L., Lawrence, D., Muir, J.F., et al. Food security: the challenge of feeding 9 billion people. Science. 2010; 327(5967): 812–818. https://doi.org/10.1126/science.1185383

Grosjean, P., Ibanez, F. Pastecs: package for analysis of space-time ecological series [software]. CRAN. 2002. https://doi.org/10.32614/CRAN.package.pastecs

Gu, Z., Gu, L., Eils, R., Schlesner, M., Brors, B. Circlize implements and enhances circular visualization in R. Bioinformatics. 2014; 30(19): 2811–2812. https://doi.org/10.1093/bioinformatics/btu393

Guidoti, D.T., Gonela, A., Vidigal, M.C.G., Conrado, T.V., Romani, I. Interrelationship between morphological, agronomic and molecular characteristics in the analysis of common bean genetic diversity. Acta Sci. Agron. 2018: 40, e33032. https://doi.org/10.4025/actasciagron.v40i1.33032

Gupta, C., Salgotra, R.K., Sharma, M., Gupta, M., Sharma, R., Gupta, S. Genetic diversity analysis of common bean (Phaseolus vulgaris L.) collected from north west Himalaya for agro-morphological traits. Plant Arch. , 2021; 21(1); 2130–2135. https://doi.org/10.51470/PLANTARCHIVES.2021.v21.no1.199

Gyang, P., Nyaboga, E., Muge, E. Molecular characterization of common bean (Phaseolus vulgaris L.) genotypes using microsatellite markers. J. Adv. Biol. Biotechnol. 2017; 13(2): 1–15. https://doi.org/10.9734/JABB/2017/33519

Gyang, P.J., Muge, E.K., Nyaboga, E.N. Genetic diversity and population structure of Kenyan common bean (Phaseolus vulgaris L.) germplasm using peroxidase gene markers. Proc. Natl. Acad. Sci. India Sect. B Biol. Sci. 2019; 90(2): 293–301. https://doi.org/10.1007/s40011-019-01101-0

Hammer, K., Teklu, Y. Plant genetic resources: selected issues from genetic erosion to genetic engineering. J. Agric. Rural Dev. Trop. Subtrop. 2008; 109(1): 15–50.

Hegay, S., Geleta, M., Bryngelsson, T., Gustavsson, L., Hovmalm, H.P., Ortiz, R. Comparing genetic diversity and population structure of common beans grown in Kyrgyzstan using microsatellites. In: Maxted, N., Dulloo, M., Ford-Lloyd, B., Frese, L., Iriondo, J., Pinheiro de Carvalho, M. (Eds.), Agrobiodiversity Conservation: Securing the Diversity of Crop Wild Relatives and Landraces. Wallingford: CABI; 2012 pp. 178–184.

International Board for Plant Genetic Resources (IBPGR). Phaseolus vulgaris descriptors. Rome: IBPGR; 1982. 32p.

Jan, S., Rather, I.A., Sofi, P.A., Wani, M.A., Sheikh, F.A., Bhat, M.A., Mir, R.R. Characterization of common bean (Phaseolus vulgaris L.) germplasm for morphological and seed nutrient traits from Western Himalayas. Legume Sci. 2021; 3(2): e86. https://doi.org/10.1002/leg3.86

Jannat, S., Shah, A.H., ul Hassan, M., Sher, A., Fiaz, S., Elesawy, B.H., et al. Genetic diversity of common bean (Phaseolus vulgaris L.) ecotypes from Pakistan using simple sequence repeats. Saudi J. Biol. Sci. 2022; 29(6): 103300. https://doi.org/10.1016/j.sjbs.2022.103300

Jose, F.C., Mohammed, M.S., Thomas, G., Varghese, G., Selvaraj, N., Dorai, M. Genetic diversity and conservation of common bean (Phaseolus vulgaris L., Fabaceae) landraces in Nilgiris. Curr. Sci. 2009 ;96(2): 227–235.

Kalia, R.K., Rai, M.K., Kalia, S., Singh, R., Dhawan, A.K. Microsatellite markers: an overview of the recent progress in plants. Euphytica. 2011; 177(3): 309–334. https://doi.org/10.1007/s10681-010-0286-9

Kassambara, A., Mundt, F. factoextra: extract and visualize the results of multivariate data analyses [software]. CRAN. 2016; https://doi.org/10.32614/CRAN.package.factoextra

KC, R.B., Joshi, B.K., Dahal, S.P. Diversity analysis and physico-morphological characteristics of indigenous germplasm of lablab bean. J. Nepal Agric. Res. Counc. 2016; 2: 15–21. https://doi.org/10.3126/jnarc.v2i0.16116

Kouam, E.B., Kamga-Fotso, A.M.A., Anoumaa, M. Exploring agro-morphological profiles of Phaseolus vulgaris germplasm shows manifest diversity and opportunities for genetic improvement. J. Agric. Food Res. 2023; 14: 100772. https://doi.org/10.1016/j.jafr.2023.100772

Kwak, M., Gepts, P. Structure of genetic diversity in the two major gene pools of common bean (Phaseolus vulgaris L., Fabaceae). Theor. Appl. Genet. 2009; 118: 979–992. https://doi.org/10.1007/s00122-008-0955-4

Lê, S., Josse, J., Husson, F. FactoMineR: an R package for multivariate analysis. J. Stat. Softw. 2008; 25: 1–18. https://doi.org/10.32614/CRAN.package.FactoMineR

Li, Y. L., & Liu, J. X. StructureSelector: A web-based software to select and visualize the optimal number of clusters using multiple methods. Molecular ecology resources. 2018; 18(1): 176-177.

Liu, B.H. Statistical Genomics: Linkage, Mapping, and QTL Analysis. Boca Raton: CRC Press; 2017. https://doi.org/10.1201/9780203738658

Liu, H.J., Wang, X., Xiao, Y., Luo, J., Qiao, F., Yang, W., et al. CUBIC: an atlas of genetic architecture promises directed maize improvement. Genome Biol. 2020; 21: 1–17. https://doi.org/10.1186/s13059-020-1930-x

Liu, K., Muse, S.V. PowerMarker: an integrated analysis environment for genetic marker analysis. Bioinformatics. 2005; 21(9): 2128–2129. https://doi.org/10.1093/bioinformatics/bti282

Loko, L.E.Y., Orobiyi, A., Adjatin, A., Akpo, J., Toffa, J., Djedatin, G., Dansi, A. Morphological characterization of common bean (Phaseolus vulgaris L.) landraces of Central region of Benin Republic. J. Plant Breed. Crop Sci. 2018; 10(11): 304–318. https://doi.org/10.5897/JPBCS2018.0766

Mamidi, S., Rossi, M., Moghaddam, S.M., Annam, D., Lee, R., Papa, R., McClean, P.E. Demographic factors shaped diversity in the two gene pools of wild common bean (Phaseolus vulgaris L.). Heredity. 2013; 110(3): 267–276. https://doi.org/10.1038/hdy.2012.82

Matondo, N.K., Yao, K.N., Kyalo, M., Skilton, R., Nkongolo, K.K., Mumba, D., et al. Assessment of the genetic diversity and the relationship among common bean (Phaseolus vulgaris L.) accessions from DR-Congo germplasm using SSR molecular markers. Int. J. Curr. Res. 2017; 9(3): 47814–47821.

McClean, P.E., Bett, K.E., Stonehouse, R., Lee, R., Pflieger, S., Moghaddam, S.M., et al. White seed color in common bean (Phaseolus vulgaris) results from convergent evolution in the P (pigment) gene. New Phytol. 2018; 219(3): 1112–1123. https://doi.org/10.1111/nph.15259

McClean, P.E., Lee, R.K., Otto, C., Gepts, P., Bassett, M.J. Molecular and phenotypic mapping of genes controlling seed coat pattern and color in common bean (Phaseolus vulgaris L.). J. Hered. 2002; 93(2): 148–152. https://doi.org/10.1093/jhered/93.2.148

Mesera, E., Shifaraw, G., Alamerew, S., Amsalu, B. Genetic variability analysis and association of traits in common bean (Phaseolus vulgaris L.) landraces collected from Ethiopia at Jimma. Adv. Agric. 2022; 4400711. https://doi.org/10.1155/2022/4400711

Ministry of Agriculture and Livestock Development. Ministry of Agriculture and Livestock Development, Government of Nepal. 2024. https://moald.gov.np (accessed January 2024).

Mohammadi, S.A., Prasanna, B.M. Analysis of genetic diversity in crop plants—salient statistical tools and considerations. Crop Sci. 2003; 43(4): 1235–1248. https://doi.org/10.2135/cropsci2003.1235

Nasar, S., Ostevik, K., Murtaza, G., Rausher, M.D. Morphological and molecular characterization of variation in common bean (Phaseolus vulgaris L.) germplasm from Azad Jammu and Kashmir, Pakistan. PLoS ONE. 2022; 17(4): e0265817. https://doi.org/10.1371/journal.pone.0265817

Neupane, R.K., Shrestha, R., Vaidya, M.L., Bhattarai, E.M., Darai, R. Agromorphological diversity in common bean (Phaseolus vulgaris L.) landraces of Jumla, Nepal. In: Kharkwal, M.C. (Ed.), Proceedings of the Fourth International Food Legumes Research Conference; Oct; New Delhi, India. 2008. pp. 639–648.

Nyakio, M., Palapala, V.A., Steele, K., Otsyula, R. Analysis of AFLP markers for screening resistance to common bean root rot (Pythium spp.). Afr. J. Biotechnol. 2015; 14(23): 1966–1974.

Okii, D., Tukamuhabwa, P., Odong, T., Namayanja, A., Mukabaranga, J., Paparu, P., Gepts, P. Morphological diversity of tropical common bean germplasm. Afr. Crop Sci. J. 2014; 22(1): 59–68.

Oliveira, T.C., Barelli, M.A.A., Oliveira, A.J., Leal, N.S., Gilio, T.A.S., Azevedo, R.F. Molecular characterization of common bean accessions using microsatellite markers. Ciênc. Agrotec 2023: 47, e003523. https://doi.org/10.1590/1413-7054202347003523

Özkan, G., Haliloğlu, K., Türkoğlu, A., Özturk, H.I., Elkoca, E., Poczai, P. Determining genetic diversity and population structure of common bean (Phaseolus vulgaris L.) landraces from Türkiye using SSR markers. Genes. 2022; 13(8); 1410. https://doi.org/10.3390/genes13081410

Papa, R., & Gepts, P. Asymmetry of gene flow and differential geographical structure of molecular diversity in wild and domesticated common bean (Phaseolus vulgaris L.) from Mesoamerica. Theoretical and applied genetics. 2003; 106(2): 239-250.

Paredes, M., Becerra, V., Tay, J. Inorganic nutritional composition of common bean (Phaseolus vulgaris L.) genotypes race Chile. Chil. J. Agric. Res. 2009; 69(4): 486–495. https://doi.org/10.4067/S0718-58392009000400002

Pathania, A., Sharma, S.K., Sharma, P.N. Common bean. In: Singh, M., Bisht, I.S., Dutta, M. (Eds.), Broadening the Genetic Base of Grain Legumes. Springer India, New Delhi; 2014 pp. 11–50. https://doi.org/10.1007/978-81-322-2023-7_2

Peakall, R., Smouse, P.E. GenAlEx 6.5: genetic analysis in Excel. Population genetic software for teaching and research—an update. Bioinformatics. 2012; 28(19): 2537–2539. https://doi.org/10.1093/bioinformatics/bts460

Powell, W., Morgante, M., Andre, C., Hanafey, M., Vogel, J., Tingey, S., & Rafalski, A. The comparison of RFLP, RAPD, AFLP and SSR (microsatellite) markers for germplasm analysis. Molecular breeding. 1996; 2(3): 225-238.

Pritchard, J.K., Stephens, M., Donnelly, P. Inference of population structure using multilocus genotype data. Genetics. 2002; 155(2): 945–959. https://doi.org/10.1093/genetics/155.2.945

R Core Team. R: A Language and Environment for Statistical Computing. Vienna, Austria: R Foundation for Statistical Computing; 2023. Available form https://www.R-project.org/

Rai, N., Kumar, S., Singh, R. K., Rai, K. K., Tiwari, G., Kashyap, S. P., ... & Rai, A. B. Genetic diversity in Indian bean (Lablab purpureus) accessions as revealed by quantitative traits and cross-species transferable SSR markers. Indian J Agric Sci. 2016; 86(9): 654-660.

Rana, J. C., Sharma, T. R., Tyagi, R. K., Chahota, R. K., Gautam, N. K., Singh, M., ... & Ojha, S. N. Characterisation of 4274 accessions of common bean (Phaseolus vulgaris L.) germplasm conserved in the Indian gene bank for phenological, morphological and agricultural traits. Euphytica. 2015; 205(2): 441-457.

Raymond, M., Rousset, F. GENEPOP (version 1.2): population genetics software for exact tests and ecumenicism. J. Hered. 1995; 86(3): 248–249. https://doi.org/10.1093/oxfordjournals.jhered.a111573

Razvi, S. M., Khan, M. N., Bhat, M. A., Mushtaq Ahmad, M. A., Ganaie, S. A., Sheikh, F. A., ... & Parry, F. A. 2018: Morphological variability and phylogenetic analysis in common bean (Phaseolus vulgaris L.).

Rebaa, F., Abid, G., Aouida, M., Abdelkarim, S., Aroua, I., Muhovski, Y., ... & Jebara, M. Genetic variability in Tunisian populations of faba bean (Vicia faba L. var. major) assessed by morphological and SSR markers. Physiology and Molecular Biology of Plants. 2017; 23(2): 397-409.

Rendón-Anaya, M., Montero-Vargas, J. M., Saburido-Álvarez, S., Vlasova, A., Capella-Gutierrez, S., Ordaz-Ortiz, J. J., ... & Herrera-Estrella, A. Genomic history of the origin and domestication of common bean unveils its closest sister species. Genome biology.2017; 18(1): 60.

Reynoso-Camacho, R., Ramos-Gomez, M., & Loarca-Pina, G. 10 Bioactive components in common beans (Phaseolus vulgaris L.). Advances in agricultural and food biotechnology. 2006.p. 217-236

Rivera, A., Plans, M., Sabaté, J., Casañas, F., Casals, J., Rull, A., & Simó, J. The Spanish core collection of common beans (Phaseolus vulgaris L.): an important source of variability for breeding chemical composition. Frontiers in Plant Science. 2018; 9: 1642.

Rodiño, A. P., Santalla, M., De Ron, A. M., & Singh, S. P. A core collection of common bean from the Iberian Peninsula. Euphytica, 2003; 131(2): 165–175. https://doi.org/10.1023/A:1023973309788

Rohlf, F.J. NTSYSpc: Numerical Taxonomy and Multivariate Analysis System, Version 2.1. Exeter Software, Setauket. 2000; NY. SAS Institute Inc., 2013. SAS/STAT® 9.4 User's Guide. SAS Institute Inc., Cary, NC.

Saba, I., Sofi, P. A., Zeerak, N. A., Mir, R. R., & Gull, M. Using augmented design for evaluation of common bean (Phaseolus vulgaris L.) germplasm. International Journal of Current Microbiology and Applied Science. 2017; 6(7): 246-254.

Savić, A., Pipan, B., Vasić, M., & Meglič, V. Genetic diversity of common bean (Phaseolus vulgaris L.) germplasm from Serbia, as revealed by single sequence repeats (SSR). Scientia Horticulturae. 2021; 288: 110405.

Scarano, Daria, Fernando Rubio, Juan José Ruiz, Rosa Rao, and Giandomenico Corrado. "Morphological and genetic diversity among and within common bean (Phaseolus vulgaris L.) landraces from the Campania region (Southern Italy)." Scientia Horticulturae 2014: 180: 72-78.

Schllemer dos Santos, A. C., Bischoff Nunes, I., Teixeira da Silva, L., de Sousa Alcântara, L. V., da Silva Domingues, L., Donazzolo, J., ... & Possenti, J. C. Morpho–Molecular Characterization of Brazilian Common Bean Landraces Varieties and Commercial Cultivars. Horticulturae. 2024; 10(11): 1123.

Schmutz, J., McClean, P. E., Mamidi, S., Wu, G. A., Cannon, S. B., Grimwood, J., ... & Jackson, S. A. A reference genome for common bean and genome-wide analysis of dual domestications. Nature genetics. 2014; 46(7): 707-713.

Shannon, C.E. A mathematical theory of communication. Bell Syst. Tech. J. 1948; 27(3): 379–423. https://doi.org/10.1002/j.1538-7305.1948.tb01338.x

Shrestha, P., & Sthapit, S. Nepal: LI-BIRD's approach to supporting community seed banks. In Community Seed Banks. 2015; (pp. 187-193). Routledge.

Singh, S.P. Patterns of variation in cultivated common bean (*Phaseolus vulgaris*, Fabaceae). Econ Bot. 1989; 43: 39–57. https://doi.org/10.1007/BF02859324

Singh, S.P., Gepts, P., Debouck, D.G.. Races of common bean (Phaseolus vulgaris L., Fabaceae). Econ. Bot. 1991; 45(3): 379–396. https://doi.org/10.1007/BF02887079

Stoilova, T., Pereira, G., de Sousa, MM, & Carnide, V. Diversity in common bean landraces (Phaseolus vulgaris L.) from Bulgaria and Portugal. Journal of Central European Agriculture. 2005; 6 (4): 443-448.

Sujata, K. C., Hira, K. M., Sundar, M. S., Basistha, A., & Jiban, S. Evaluation of common bean (Phaseolus vulgaris L.) genotypes against anthracnose (Colletotrichum lindemuthianum Sacc. and Magn.). 2021.

Tajima, F.. Statistical method for testing the neutral mutation hypothesis by DNA polymorphism. Genetics .1989; 123(3): 585–595. https://doi.org/10.1093/genetics/123.3.585

Tessier, C., David, J., This, P., Boursiquot, J.M., Charrier, A. Optimization of the choice of molecular markers for varietal identification in Vitis vinifera L. Theor. Appl. Genet. 1999; 98(1): 171–177. https://doi.org/10.1007/s001220051046

Tigist, S. G., Sibiya, J., Amelework, A., & Keneni, G. Agromorphological and Physiological Performance of Ethiopian Common Bean (Phaseolus vulgaris L.) Genotypes under Different Agroecological Conditions. Plants. 2023. 12(12), 2342. https://doi.org/10.3390/plants12122342

van Hintum, T.J.L., Brown, A.H.D., Spillane, C., Hodgkin, T. Core Collections of Plant Genetic Resources. IPGRI Technical Bulletin No. 3. IPGRI, Rome. 2002.

Voysest, O., & Dessert, M. Bean cultivars: classes and commercial seed types. Common beans: research for crop improvement. 1991.pp. 119-162.

Wang, X., Lee, S., Choi, Y., Yi, J., Desta, K. T., Shin, M., Lee, G., Jeon, Y., & Yoo, E. Evaluation of genetic diversity and population structure of wheat core collection using SNP and KASP markers for major agronomic traits. BMC Plant Biology. 2025; 25(1): 904. https://doi.org/10.1186/s12870-025-06926-8

Weir, B.S., Cockerham, C.C. Estimating F-statistics for the analysis of population structure. Evolution. 1984; 38(6): 1358–1370. https://doi.org/10.2307/2408641

Wright, S. The genetical structure of populations. Ann. Eugen. 1951; 15(1): 323–354. https://doi.org/10.1111/j.1469-1809.1949.tb02451.x

Wright, S.. The interpretation of population structure by F-statistics with special regard to systems of mating. Evolution. 1965; 19(3): 395–420. https://doi.org/10.2307/2406450

Yu, K., Park, S. J., Poysa, V., & Gepts, P. Integration of simple sequence repeat (SSR) markers into a molecular linkage map of common bean (Phaseolus vulgaris L.). Journal of Heredity. 2002; 91(6): 429-434.

Zargar, S. M., Farhat, S., Mahajan, R., Bhakhri, A., & Sharma, A. Unraveling the efficiency of RAPD and SSR markers in diversity analysis and population structure estimation in common bean. *Saudi* journal of biological sciences. 2016; 23(1): 139-149.

Zeven, A. C. Landraces: a review of definitions and classifications. Euphytica. 1998; *104*(2): 127-139.

Zhang, X., Blair, M. W., & Wang, S. Genetic diversity of Chinese common bean (Phaseolus vulgaris L.) landraces assessed with simple sequence repeat markers. Theoretical and Applied Genetics. 2008; 117(4): 629-640.

Declarations funding source

The authors declare that no funds, grants, or other support was received during the preparation of this manuscript.

Declaration of competing interests

The authors have no relevant financial or non-financial interests to disclose.

Declaration of generative AI use

The authors declare that no generative AI or AI-assisted technologies were used in the preparation of this manuscript.

Author Contributions

Conceptualization: Pradip Thapa, Sandip Bohara, Bal Krishna Joshi

Methodology: Pradip Thapa, Bal Krishna Joshi, Sanjay Raut

Investigation: Sandip Bohara

Software: Sandip Bohara, Promise Shrestha

Data Curation: Sandip Bohara, Promise Shrestha

Formal Analysis: Sandip Bohara, Promise Shrestha

Resources: NAGRC molecular Lab

Visualization: Sandip Bohara, Promise Shrestha

Writing-Original Draft: Sandip Bohara, Promise Shrestha, Sanjay Raut

Writing-Review & Editing: Sandip Bohara, Promise Shrestha, Pradip Thapa, Bal Krishna Joshi

Supervision and Validation: Sanjay Raut, Bal Krishna Joshi, Pradip Thapa

Data availability

The datasets generated during and/or analyzed during the current study are available from the corresponding author upon reasonable request.

Supplementary table

Annex Table 1. Information about the collection of 58 landraces from 24 districts of Nepal

S. N	Accessions	Collected district	Lattitude	Longitude	Altitude
1	NGRCO 9504	Khotang	27.21567	86. 795202	1523m
2	NGRCO 9505	Khotang	27.22415	86. 78719	1668m
3	NGRCO 9506	Khotang	27.22415	86. 78719	1668m
4	NGRCO 9507	Tanahun	28.001	83.9131	879m
5	NGRCO 9514	Dhankhuta	27. 0095	87. 31353	1490m
6	NGRCO 9516	Dhankhuta	27. 0011	87. 308	1380m
7	NGRCO 9515	Dhankhuta	27. 00244	87. 30722	1431m
8	NGRCO 1760	Kavrepalanchok	27.31	85.38	800m
9	NGRCO 2887	Sindhupalchok	27.9300	85.5800	1100m
10	NGRCO 9555	Bara	26. 88	85. 16	32m
11	NGRCO 1765	Sindhupalchowk	27.57	85.41	1100m
12	NGRCO 2894	Sankhuwasaaba	27.41	86.44	2300m
13	NGRCO 9613	Sunsari	26. 7466	87. 153185	109m
14	NGRCO 1680	Lamjung	28.2199	84.4852	1402m
15	NGRCO 1686	Lamjung	28.2745	84.3790	1981m
16	NGRCO 2280	Jajarkot	29.2748	82.1840	1800m
17	NGRCO 9919	Jajarkot	28. 79561	82. 16664	1783m
18	NGRCO 9920	Jajarkot	28. 79561	82. 16664	1783m
19	NGRCO 1703	Bajura	29.4900	81.4600	2675m
20	NGRCO 1704	Bajura	29.4900	81.4600	2675m
21	NGRCO 9916	Chitwan	27. 61861	84. 40333	195m
22	NGRCO 9917	Chitwan	27. 61861	84. 40333	195m
23	NGRCO 1713	Achham	28.2789	83.8501	1648m
24	NGRCO 10200	Jumla	29. 2425	82. 10528	2277m
25	NGRCO 10197	Jumla	29. 2425	82. 10528	2277m
26	NGRCO 2895	Humla	29.6767	81.9137	1350m
27	NGRCO 4168	Myagdi	28.3717	83.6066	1100m
28	NGRCO 2284	Mustang	28.8201	83.8471	3450m
29	NGRCO 2373	Mustang	28.8201	83.8471	3450m
30	NGRCO 10335	Kailali	29 . 597	80. 489	1314m
31	NGRCO 10334	Kailali	29 . 597	80 . 489	1314m
32	NGRCO 10337	Baitadi	29 . 597248	80 . 489687	1315m
33	NGRCO 10336	Kailali	29 . 597	80 . 489	1314m

34	NGRCO 10644	Solukhumbu	27.41	86.44	1893m
35	NGRCO 10645	Morang	26 . 736657	87 . 514838	271m
36	NGRCO 10646	Dhankhuta	27 . 00841	86 . 31093	1485m
37	NGRCO 10647	Dhankhuta	27 . 00956	87 . 31090	1530m
38	NGRCO 10648	Panchthar	26 . 93777	86 . 687678	1600m
39	NGRCO 10649	Panchthar	26 . 93777	86 . 687678	1600m
40	NGRCO 10650	Panchthar	26 . 93777	86 . 687678	1600m
41	NGRCO 10651	Panchthar	26 . 93777	86 . 687678	1600m
42	NGRCO 10652	Panchthar	26. 91667	87. 68333	1699m
43	NGRCO 10653	Panchthar	26 . 86667	87 . 66667	1623m
44	NGRCO 10654	Panchthar	26 . 86667	87 . 66667	1623m
45	CO 11571	Makwanpur	27.6441	85.1184	1701m
46	CO 14669	Dolakha	27 . 64993311	85 . 960618	2200m
47	CO 14962	Lalitpur	27 . 479894	85 . 236181	1700m
48	CO 15426	Okhaldhunga	27 . 307183	86 . 330297	1250m
49	CO 14593	Sindhupalchowk	27 . 683442	85 . 892030	2000m
50	CO 14956	Lalitpur	17 . 492007	85 . 247828	1700m
51	CO 15042	Baglung	28 . 131	83 . 372	1840m
52	CO 14915	Dhading	27 . 80204	84 . 880560	462m
53	CO 14570	Sindhupalchowk	27 . 677912	86 . 934377	2500m
54	CO 15473	Bhojpur	27 . 165	87.055	1500m
55	CO 13374	Dhankhuta	27 . 00091	87 . 3087	1371m
56	Trishuli	Dolakha	27.46	86.10	1596m
57	NGRCO 8500	Dolakha	27.46	86.10	1596m
58	NGRCO 8503	Dolakha	27.46	86.10	1727m

Annex Table. 2 List of 23 quantitative characters studied among the common bean landraces

S.N	Quantitative traits	Unit	Crop growth stage	Description of character
1	Days to 80% germination	count	Vegetative stage	Number of days taken by seed to germinate by 80%
2	Chlorophyll content	CCI	Vegetative stage	Measured from 5 plants from different positions
3	Leaf length	cm	Vegetative stage	Measured on terminal leaflet of third trifoliate leaf from pulvinus to leaf tip
4	Leaf breadth	cm	Vegetative stage	Measured on terminal leaflet of third trifoliate leaf across the leaf diameter
5	Days to first flowering	count	Reproductive stage	Number of days from emergence to stage where 50% of plants have set flowers
6	Days to 50% flowering	count	Reproductive stage	Number of days from emergence to stage where first plant set flowers
7	Dyas to first fruiting	count	Reproductive stage	Number of days from emergence to stage where first plant set fruit
8	Days to 50% fruiting	count		Number of days from emergence to stage where 50% of plants have set fruits
9	Number of flower bud/raceme	count	Reproductive stage	Average number of flower buds counted from 5 random plants raceme
10	Number of raceme/plants	count	Reproductive stage	Average number of racemes counted from 5 random plants
11	Pod length	mm	Reproductive stage	Average length of the largest fully expanded immature pods measured from 5 random plants by vernier caliper

12	Pod width	mm	Reproductive stage	Average width of the largest fully expanded immature pods measured from 5 random plants by vernier caliper
13	Plant height	cm	Maturity stage	Measured from cotyledon scar to the tip of plant
14	Stem diameter	mm	Maturity stage	Measured by vernier caliper at crop maturity
15	Number of nodes/plants	count	Maturity stage	Counting the number of nodes from 5 random plants
16	Number of pods/plants	count	Maturity stage	Average number of pods from 5 plants
17	Number of seed/pods	count	Maturity stage	Average number of seed from one pod
18	Days to maturity	count	Maturity stage	Number of days from emergence until 90% of pod are mature
19	Seed length	mm	After harvesting stage	Measured parallel to the hilum by vernier caliper
20	Seed breadth	mm	After harvesting stage	Measured the diameter between the seed by vernier caliper
21	Seed thickness	mm	After harvesting stage	Measured from hilum to opposite site by vernier caliper
22	Thousand seed weight	g	After harvesting stage	Weight of 100 seed measured by weighing balance at moisture content of 12-14%
23	Total yield	t/ha	After harvesting stage	Converting the gram per meter square into tons per hectare

Annex Table. 3 List of 24 qualitative characters studied among the common bean landraces

S.N	Qualitative traits	Crop growth stage	Description of character
1	Hypocotyl pigmentation	Vegetative stage	1=Purple, 2=green
2	Emerging cotyledon color	Vegetative stage	3=Green, 4=white, 6=others
3	Leaf color of chlorophyll	Vegetative stage	3=Pale green, 5=medium green 7=dark green
4	Leaf hairiness	Vegetative stage	3=Low, 5=medium, 7=dark green
5	Leaf shape	Vegetative stage	1=Triangular, 2=quadrangular
6	Flower color	Reproductive stage	1=White, 3=lilac, 7=dark lilac with purple spots
7	Plant type	Reproductive stage	1=Determinate bush, indeterminate bush with erect branches, 3=Indeterminate bush with produce branches, 4=Indeterminate with semi climbing main stem and branching, 5= Indeterminate with moderate climbing ability and pods are distributed evenly upto the plants, 6=Indeterminate with aggressive climbing and pods are mainly on the upper nodes of the plant
8	Bracteole size	Reproductive stage	3= Small, 5=medium, 7=large
9	Bracteole shape	Reproductive stage	3= Lanceolate, 7=Ovate
10	Flower bud size	Reproductive stage	3=Small, 5=medium, 7-large
11	Pod curvature	Reproductive stage	3-Straight, 5=slightly curved, 7=curved
12	Pod beak orientation	Reproductive stage	3=Upward, 5=straight, 7=downward
13	Pod beak position	Reproductive stage	1=Marginal, 2=non-marginal, 3=others
14	Wing/pod opening	Reproductive stage	3=Parallel closed wings, 5=wings moderately diverging, 7=wings widely diverging
15	Pod color	Maturity stage	1=Dark purple, 3=purple strips on green, 4=carmine strips on green, 6=dark pink, 7=normal green, 8=shiny green, 9=dull green or silver grey
16	Seed coat pattern	After harvesting	0=Absent, 1=constant mottled, 2=stripped, 3=rhomboid spotted, 4=speckled, 5=circular mottling, 7=broad striped

17	Seed coat color	After harvesting	1=Black, 2=brown pale to dark, 3=maroon, 5=yellow to greenish yellow, 6=pale cream to buff, 7=pure white, 8=whitish, 9=white purple tinged, 12=red
18	Seed shape	After harvesting	1=Round, 2=oval, 3=cuboid, 4=kidney shaped, 5=truncate fastigiate, 3=matt, 5=medium, 7=shiny
19	Brilliance of the seed	After harvesting	3=matt, 5=medium, 7=shiny
20	Number of seed color	After harvesting	1=Black, 2=brown pale to dark, 3=maroon, 4=gray, brownish to greenish, 5=yellow to greenish yellow
21	Primary/main seed color	After harvesting	1-Black, 2-brown, pale to dark, 3-maroon, 5-yellow to greenish yellow, 6-pale cream to buff, 7-pure white, 8-whitish, 9-white, purple tinged, 12-red
22	Secondary seed color	After harvesting	1=Black, 2=brown, pale to dark, 3=maroon, 5=yellow to greenish yellow, 6=pale cream to buff, 7=pure white, 8=whitish, 9=white, purple tinged, 12=red
23	Seed veining	After harvesting	+=Absent, 0=present
24	Seed hilum colour	After harvesting	8=Whitish, 12=red

Annex Table 4. Details of 15 SSR primers, sequences and annealing temperature used in the research

S.N	Marker name	Forward primer	Reverse primer	Annealing temperature
1	PV-cct001	CCAACCACATTCTTCCCTACGT C	GCGAGGCAGTTATCTTTAGGAGTG	56.9
2	BM143	GGGAAATGAACAGAGGAA A	ATGTTGGGAACTTTTAGTGTG	54.5
3	BM170	AGCCAGGTGCAAGACCTTAG	AGATAGGGAGCTGGTGGTAGC	52.1
4	BM184	AGTGCTCTATCAAGATGTGTG	ACATAATCAATGGGTCACTG	50.5
5	BM187	TTTCTCCAACTCACTCCTTTCC	TGTGTTTGTGTTCCGAATTATGA	48.1
6	PV-BR35	TCTACGCGTTCCCTCTGTCT	AGTGGATGTGTGGGAAAAGC	52.1
7	PV-BR167	GGCAAAAACAAAACCATTTCA	GCCATTTCTCCACTGTCTGG	43.8
8	PV-BR185	TGGTAAAGCAAA ACGATGG	GACAGAAGAGTGAGGGTGTGAA	50.5
9	SSR-IAC10	AGGAACTAAAAGCCGAACTGG	GCCTCCGCCGATCAACACTA	50.5
10	SSR-IAC62	AACCCGTGAATCTTTGAGG	ATTGATGGTGGATTTTGAA	49.5
11	BMd-12	CATCAACAAGGACAGCCTCA	GCAGCTGGCGGGTAAAACAG	52.1
12	BMd-25	GCAGATCGCCTACTCACAAA	CGTTGACGAGAAGCATCAAG	55.7
13	BMd-26	CTTGCCTTGTGCTTCCTTCT	TCCATTCCCAACCAAGTTTC	53.2
14	BM152	CCGGGACTTGCCAGAAGAAC	AAGAGGAGGTCGAAACCTTAAATC G	50.5
15	BM210	CCCTCATCCTCCATTCTTATCG	ACCACTGCAATCCTCATCTTTG	50.5

Appendix 5. Denaturation, annealing and extension temperature used for 15 SSR markers

For primers 1-10

For primer 1-10	Temperature	Time
Initial denaturation	92-degree celsius	5 minutes

Final denaturation	92-degree celsius	1 minutes (60 second)
Annealing	56.9-degree celcius	1 minutes (60seconds)
Extension	72-degree celsius	2 minutes
Final extension	72-degree celsius	5 minutes
	4-degree celsius	infinite time

For primers 10-13

For primer 10-13	Temperature	Time
Initial denaturation	92-degree celsius	5 minutes
Final denaturation	92-degree celsius	1 minutes (60 second)
Annealing	56.9-degree celcius	1 minutes (60 seconds)
Extension	72-degree celsius	2 minutes
Final extension	72-degree celsius	5 minutes
	4-degree celsius	infinite time

For primers 13-15

For primer 13-15	Temperature	Time
Initial denaturation	94-degree celsius	2 minutes
Final denaturation	94-degree celsius	(15 seconds)
Annealing	56.9-degree celcius	(15 seconds)
Extension	72-degree celsius	15 second
Final extension	72 degree celsius	15 seconds
	4-degree celsius	infinite time
