# Supplementary material for: Morphological and molecular characterization of Nepalese Common Bean (Phaseolus vulgaris L.) Landraces
Source: PLoS One. 2026 Jul 30;21(7):e0354479. doi: 10.1371/journal.pone.0354479 (PMC13423178; doi:10.1371/journal.pone.0354479)
Supplement: Attachment — Submitted filename: PONE-D-26-11665_Comments to the Editor.docx [file pone.0354479.s006.docx]

**Comments to the Editor**

The manuscript entitled *“Morphological and Molecular Diversity of Phaseolus vulgaris (common bean) from Nepal”* addresses an important topic and combines both morphological and molecular analyses, which is a strong and valuable approach. The dataset appears substantial, and the study has the potential to make a meaningful contribution to the field of plant genetic diversity and breeding.

However, in its current form, the manuscript suffers from serious issues in presentation and organization that significantly hinder its scientific clarity and readability. The writing quality is poor, with numerous grammatical and stylistic errors throughout the text. More importantly, the structure of the manuscript does not follow standard scientific conventions: results and discussion are often mixed, making it difficult to distinguish between observed findings and their interpretation. There are also serious methodological concerns: The formula used for calculating the diversity index appears to be incorrect and should be carefully verified. The diversity index has been applied to quantitative data without appropriate categorization, and its interpretation is not appropriate. As presented, the interpretation does not reflect standard usage (e.g., evenness of distribution when applied to such data).

In addition, the manuscript contains excessive and unnecessary information, particularly in the Materials and Methods and Results sections, which further reduces clarity. The referencing system is inconsistent and problematic. Citations are numbered in the text, but the reference list is not properly numbered, making it extremely difficult to verify sources or follow the scientific argumentation.

The manuscript editing style does not reflect PLOSOne style.

Overall, while the scientific effort and dataset are valuable, the manuscript requires substantial revision in language, structure, and methodological rigor before it can be considered for publication.

Recommendation: Major revision
