# Supplementary material for: Morphological and molecular characterization of Nepalese Common Bean (Phaseolus vulgaris L.) Landraces
Source: PLoS One. 2026 Jul 30;21(7):e0354479. doi: 10.1371/journal.pone.0354479 (PMC13423178; doi:10.1371/journal.pone.0354479)
Supplement: Attachment — Submitted filename: PONE-D-26-11665_comments to author.docx [file pone.0354479.s007.docx]

**Comments to the authors**

Your study on *Phaseolus vulgaris* diversity in Nepal is valuable and reflects significant research effort, particularly in combining morphological and molecular analyses. However, the manuscript in its current form does not clearly communicate the scientific contribution due to major issues in writing, organization, and methodology.

Please consider the following points:

a) Language and writing quality

The manuscript contains a lot of grammatical and stylistic errors. A thorough revision by a proficient English speaker or a professional editing service is strongly recommended.

b) Organization of the Manuscript

Results and discussion are frequently mixed; these sections should be clearly separated. Interpretations and comparisons with the literature should be moved to the Discussion section.

The overall flow from Materials and Methods to Results and Conclusion needs to be clarified and logically structured.

The manuscript editing does not follow the PLOSOne standard.

c) Methodological issues

There are important concerns regarding the methodology:

i. The formula used to calculate the diversity index appears to be incorrect and must be carefully checked and corrected.
ii. The diversity index has been calculated using quantitative data without clear categorization. Typically, diversity indices are applied to qualitative (categorical) data.
iii. If applied to quantitative data, appropriate categorization is required, and the interpretation should reflect distribution properties such as evenness or dominance. The current interpretation is therefore not appropriate and should be revised.
d) Clarity and conciseness

The manuscript contains a significant amount of unnecessary or redundant information, particularly in materials and methods results Please revise these sections to focus on essential information that directly supports the objectives and findings of the study.

Furthermore, some parts of the methodology are not clearly described; please provide clear and logically organized methodological steps. Present results clearly and separately from discussion.

e) . Referencing issues

Citations are numbered in the text, but the reference list is not correspondingly numbered. This makes it difficult to verify sources and follow the scientific argument.
Please ensure consistency and proper formatting of all references.

Final Remark

I have included detailed, specific comments directly within the annotated manuscript to guide your revision. Your study has strong potential, but substantial improvements in writing, structure, and methodological accuracy are required to make the work clear, rigorous, and suitable for publication.
