# Supplementary material for: Morphological and molecular characterization of Nepalese Common Bean (Phaseolus vulgaris L.) Landraces
Source: PLoS One. 2026 Jul 30;21(7):e0354479. doi: 10.1371/journal.pone.0354479 (PMC13423178; doi:10.1371/journal.pone.0354479)
Supplement: Attachment — Submitted filename: Response to Reviewers.docx [file pone.0354479.s009.docx]

**Response to Reviewer 1**

1. The Shannon–Weaver diversity index values for quantitative traits reported in the abstract were verified and corrected accordingly.
2. The yield unit was standardized to t/ha, and the repeated manuscript title was removed from the keywords section.
3. Additional relevant references were incorporated to support the gene pool seed weight threshold statement. Furthermore, the Nepal bean area and production statistics were updated using the latest available data from FAOSTAT (2024), replacing the previous 2023 dataset.
4. Details regarding the augmented block design, including the number of blocks and check varieties, were clarified. The manuscript also specifies that five plants per accession were sampled for quantitative trait evaluation and clarifies the nature of the replicates used.
5. The high thousand-seed weight observed for accession CO14570 (1236 g) was verified and retained, as the accession also exhibited comparatively larger seed dimensions, including seed length (20.35 mm), seed breadth (13.60 mm), and seed thickness (9.06 mm), as documented in the data repository.
6. The modified CTAB DNA extraction protocol was briefly described for clarity. Additionally, PCR annealing temperatures for each primer were included in the revised manuscript in the supplementary information section.
7. In Table 2, some H′ values appear similar due to rounding effects, and this clarification has been added to the manuscript. The PCA section was also revised to clearly state that principal components with eigenvalues greater than 1 (PC1–PC5) were retained for analysis.
8. During molecular data analysis, three monomorphic SSR markers were excluded, and only the 12 polymorphic markers were retained for further analysis.
9. In the discussion section, the observation of H₀ > Hₑ was further explained by considering the possible presence of null alleles as an alternative explanation.
10. The conclusion section was improved to better describe the performance of the evaluated landraces. The revised statement now indicates that several landraces outperformed the three check varieties for important reproductive and yield-related traits, including earliness and yield components.
11. The dataset has been deposited in a public Zenodo repository. In addition, the number of references was expanded from 100 to 112, and all references were reformatted according to the PLOS ONE journal guidelines.

**Response to Reviewer 2**

1. The manuscript language was thoroughly revised to improve grammatical accuracy, readability, and overall writing quality.
2. The organization of the manuscript was improved, and the Results and Discussion sections are now clearly separated.
3. The formula used to calculate the Shannon–Weaver diversity index was carefully rechecked and corrected where necessary.
4. The interpretation of the H′ diversity index for both quantitative and qualitative traits was clarified. For quantitative traits, a high H′ value indicates an even distribution of accessions across SD-based classes, whereas a low H′ value indicates concentration within a narrow phenotypic range. For qualitative traits, a high H′ value reflects an even distribution across multiple descriptor states, while a low H′ value indicates dominance of one or a few phenotypic classes.
5. Citation numbering within the manuscript was corrected, and all in-text citations were appropriately linked to the corresponding reference list entries.
6. Redundant and unnecessary information, particularly within the Materials and Methods and Results sections, was removed or revised. The methodology was rewritten in a clearer and more logically organized manner, and the Results section was distinctly separated from the Discussion section.
