# Supplementary material for: Morphological and molecular characterization of Nepalese Common Bean (Phaseolus vulgaris L.) Landraces
Source: PLoS One. 2026 Jul 30;21(7):e0354479. doi: 10.1371/journal.pone.0354479 (PMC13423178; doi:10.1371/journal.pone.0354479)
Supplement: Peer review history

# Peer review: Decision Letter 0

**DOI:** 10.1371/journal.pone.0354479.r001

**Authors:** Tzen-Yuh Chiang

25 Apr 2026

PONE-D-26-11665

Morphological and Molecular Characterization of Nepalese Common Bean (Phaseolus vulgaris L.) Landraces

PLOS One

Dear Dr.  Thapa,

Thank you for submitting your manuscript to PLOS ONE. After careful consideration, we feel that it has merit but does not fully meet PLOS ONE’s publication criteria as it currently stands. Therefore, we invite you to submit a revised version of the manuscript that addresses the points raised during the review process.

Please submit your revised manuscript by Jun 09 2026 11:59PM. If you will need more time than this to complete your revisions, please reply to this message or contact the journal office at plosone@plos.org. When you're ready to submit your revision, log on to https://www.editorialmanager.com/pone/ and select the 'Submissions Needing Revision' folder to locate your manuscript file.

Please include the following items when submitting your revised manuscript:

- A letter that responds to each point raised by the academic editor and reviewer(s). You should upload this letter as a separate file labeled 'Response to Reviewers'.
- A marked-up copy of your manuscript that highlights changes made to the original version. You should upload this as a separate file labeled 'Revised Manuscript with Track Changes'.
- An unmarked version of your revised paper without tracked changes. You should upload this as a separate file labeled 'Manuscript'.

If you would like to make changes to your financial disclosure, please include your updated statement in your cover letter. Guidelines for resubmitting your figure files are available below the reviewer comments at the end of this letter.

If applicable, we recommend that you deposit your laboratory protocols in protocols.io to enhance the reproducibility of your results. Protocols.io assigns your protocol its own identifier (DOI) so that it can be cited independently in the future. For instructions see: https://journals.plos.org/plosone/s/submission-guidelines#loc-laboratory-protocols. Additionally, PLOS ONE offers an option for publishing peer-reviewed Lab Protocol articles, which describe protocols hosted on protocols.io. Read more information on sharing protocols at https://plos.org/protocols?utm_medium=editorial-email&utm_source=authorletters&utm_campaign=protocols.

As the corresponding author, your ORCID iD is verified in the submission system and will appear in the published article. PLOS supports the use of ORCID, and we encourage all coauthors to register for an ORCID iD and use it as well. Please encourage your coauthors to verify their ORCID iD within the submission system before final acceptance, as unverified ORCID iDs will not appear in the published article. *Only*  the individual author can complete the verification step; PLOS staff *cannot*  verify ORCID iDs on behalf of authors.

We look forward to receiving your revised manuscript.

Kind regards,

Tzen-Yuh Chiang

Academic Editor

PLOS One

**Journal Requirements:**

When submitting your revision, we need you to address these additional requirements.

1. Please ensure that your manuscript meets PLOS ONE's style requirements, including those for file naming. The PLOS ONE style templates can be found at

https://journals.plos.org/plosone/s/file?id=wjVg/PLOSOne_formatting_sample_main_body.pdf and

https://journals.plos.org/plosone/s/file?id=ba62/PLOSOne_formatting_sample_title_authors_affiliations.pdf

2. In your Methods section, please provide additional information regarding the permits you obtained for the work. Please ensure you have included the full name of the authority that approved the field site access and, if no permits were required, a brief statement explaining why.

3. We note that you have indicated that there are restrictions to data sharing for this study. PLOS only allows data to be available upon request if there are legal or ethical restrictions on sharing data publicly. For more information on unacceptable data access restrictions, please see http://journals.plos.org/plosone/s/data-availability#loc-unacceptable-data-access-restrictions.

Before we proceed with your manuscript, please address the following prompts:

a) If there are ethical or legal restrictions on sharing a de-identified data set, please explain them in detail (e.g., data contain potentially identifying or sensitive patient information, data are owned by a third-party organization, etc.) and who has imposed them (e.g., a Research Ethics Committee or Institutional Review Board, etc.). Please also provide contact information for a data access committee, ethics committee, or other institutional body to which data requests may be sent.

b) If there are no restrictions, please upload the minimal anonymized data set necessary to replicate your study findings to a stable, public repository and provide us with the relevant URLs, DOIs, or accession numbers. For a list of recommended repositories, please see

https://journals.plos.org/plosone/s/recommended-repositories. You also have the option of uploading the data as Supporting Information files, but we would recommend depositing data directly to a data repository if possible.

We will update your Data Availability statement on your behalf to reflect the information you provide.

4. We note that Figure 2 in your submission contain map images which may be copyrighted. All PLOS content is published under the Creative Commons Attribution License (CC BY 4.0), which means that the manuscript, images, and Supporting Information files will be freely available online, and any third party is permitted to access, download, copy, distribute, and use these materials in any way, even commercially, with proper attribution. For these reasons, we cannot publish previously copyrighted maps or satellite images created using proprietary data, such as Google software (Google Maps, Street View, and Earth). For more information, see our copyright guidelines: http://journals.plos.org/plosone/s/licenses-and-copyright.

We require you to either (1) present written permission from the copyright holder to publish these figures specifically under the CC BY 4.0 license, or (2) remove the figures from your submission:

1. You may seek permission from the original copyright holder of Figure(s) [#] to publish the content specifically under the CC BY 4.0 license.

We recommend that you contact the original copyright holder with the Content Permission Form (http://journals.plos.org/plosone/s/file?id=7c09/content-permission-form.pdf) and the following text:

“I request permission for the open-access journal PLOS ONE to publish XXX under the Creative Commons Attribution License (CCAL) CC BY 4.0 (http://creativecommons.org/licenses/by/4.0/). Please be aware that this license allows unrestricted use and distribution, even commercially, by third parties. Please reply and provide explicit written permission to publish XXX under a CC BY license and complete the attached form.”

Please upload the completed Content Permission Form or other proof of granted permissions as an "Other" file with your submission.

In the figure caption of the copyrighted figure, please include the following text: “Reprinted from [ref] under a CC BY license, with permission from [name of publisher], original copyright [original copyright year].”

2. If you are unable to obtain permission from the original copyright holder to publish these figures under the CC BY 4.0 license or if the copyright holder’s requirements are incompatible with the CC BY 4.0 license, please either i) remove the figure or ii) supply a replacement figure that complies with the CC BY 4.0 license. Please check copyright information on all replacement figures and update the figure caption with source information. If applicable, please specify in the figure caption text when a figure is similar but not identical to the original image and is therefore for illustrative purposes only.

The following resources for replacing copyrighted map figures may be helpful:

USGS National Map Viewer (public domain): http://viewer.nationalmap.gov/viewer/

The Gateway to Astronaut Photography of Earth (public domain): http://eol.jsc.nasa.gov/sseop/clickmap/

Maps at the CIA (public domain): https://www.cia.gov/library/publications/the-world-factbook/index.html and https://www.cia.gov/library/publications/cia-maps-publications/index.html

NASA Earth Observatory (public domain): http://earthobservatory.nasa.gov/

Landsat: http://landsat.visibleearth.nasa.gov/

USGS EROS (Earth Resources Observatory and Science (EROS) Center) (public domain): http://eros.usgs.gov/#

Natural Earth (public domain): http://www.naturalearthdata.com/

5. We notice that your supplementary tables are included in the manuscript file. Please remove them and upload them with the file type 'Supporting Information'. Please ensure that each Supporting Information file has a legend listed in the manuscript after the references list.

6. If the reviewer comments include a recommendation to cite specific previously published works, please review and evaluate these publications to determine whether they are relevant and should be cited. There is no requirement to cite these works unless the editor has indicated otherwise.

[Note: HTML markup is below. Please do not edit.]

Reviewers' comments:

Reviewer's Responses to Questions

**Comments to the Author**

1. Is the manuscript technically sound, and do the data support the conclusions?

The manuscript must describe a technically sound piece of scientific research with data that supports the conclusions. Experiments must have been conducted rigorously, with appropriate controls, replication, and sample sizes. The conclusions must be drawn appropriately based on the data presented.

Reviewer #1: Yes

Reviewer #2: Yes

**********

2. Has the statistical analysis been performed appropriately and rigorously?

Reviewer #1: Yes

Reviewer #2: No

**********

3. Have the authors made all data underlying the findings in their manuscript fully available?

The PLOS Data policy requires authors to make all data underlying the findings described in their manuscript fully available without restriction, with rare exception (please refer to the Data Availability Statement in the manuscript PDF file). The data should be provided as part of the manuscript or its supporting information, or deposited to a public repository. For example, in addition to summary statistics, the data points behind means, medians and variance measures should be available. If there are restrictions on publicly sharing data—e.g. participant privacy or use of data from a third party—those must be specified.

Reviewer #1: No

Reviewer #2: Yes

**********

4. Is the manuscript presented in an intelligible fashion and written in standard English?

PLOS ONE does not copyedit accepted manuscripts, so the language in submitted articles must be clear, correct, and unambiguous. Any typographical or grammatical errors should be corrected at revision, so please note any specific errors here.

Reviewer #1: Yes

Reviewer #2: No

**********

5. Review Comments to the Author

Please use the space provided to explain your answers to the questions above. You may also include additional comments for the author, including concerns about dual publication, research ethics, or publication ethics. (Please upload your review as an attachment if it exceeds 20,000 characters)

Reviewer #1: The short title on page 1 reads "Diversity in Nepalese in common bean" — correct to "Diversity in Nepalese Common Bean" or similar.

Abstract – quantitative H' range: The Shannon-Weaver index for quantitative traits is given as 0.46–0.90, but Table 1 shows values as low as 0.74 for some traits. Please verify and align.

Abstract – missing units: Add units for yield (t/ha) where first mentioned.

Keywords – repetition: "Nepalese bean landraces" repeats "Nepalese" from the title; consider simplifying to "common bean landraces".

Reference [3] appears incomplete (no title/DOI). Please check all references against PLOS ONE style.

Gene pool seed weight thresholds: The statement >40g/100 seed weight (Andean) and <25g (Mesoamerican) lacks a citation for the 40g threshold.

Nepal bean area/production data: The 2023 FAOSTAT data cited may already be outdated; consider using 2024 or 2025 data if available or note the year explicitly.

You state "augmented block design" but do not specify number of blocks or how checks were arranged. Add one sentence.

For quantitative traits, you sampled 5 plants per accession. State, whether these were technical replicates or biological replicates.

CTAB protocol: Cite the modified CTAB protocol correctly (Doyle & Doyle 1987 is listed, but "modified" needs a brief description of key changes).

PCR annealing temperatures: Annex Table 4 and Appendix 5 show conflicting annealing temps for some primers (e.g., BM143). Unify.

Table 2 – H' values: Some H' values appear duplicated (e.g., leaf shape H'=0.99, bracteole shape H'=0.99). Are these identical by chance or rounding? Please comment briefly.

PCA – eigenvalue >1: State, that you retained components with eigenvalue >1 (PC1–PC5).

Cluster V seed weight: 1236 g for thousand seed weight seems extremely high (typical max ~800 g). Check data entry or clarify if this is a misprint.

Molecular data – monomorphic markers: You had 3 monomorphic SSRs out of 15. List them or state they were excluded from further analysis.

Discussion – You state H₀ > Hₑ indicates excess heterozygotes. For a self-pollinating species, discuss possible genotyping error (e.g., null alleles) as an alternative explanation.

Conclusion – "Many landraces outperformed check varieties" – only two checks were used. Soften to "several" or specify which traits.

Some DOIs are hyperlinked, others are plain text. Make uniform.

You state data available upon request. PLOS ONE strongly prefers public deposition. Consider adding a note that data will be deposited in a public repository upon acceptance.

Reviewer #2: Comments to the authors

Your study on Phaseolus vulgaris diversity in Nepal is valuable and reflects significant research effort, particularly in combining morphological and molecular analyses. However, the manuscript in its current form does not clearly communicate the scientific contribution due to major issues in writing, organization, and methodology.

Please consider the following points:

a) Language and writing quality

The manuscript contains a lot of grammatical and stylistic errors. A thorough revision by a proficient English speaker or a professional editing service is strongly recommended.

b) Organization of the Manuscript

Results and discussion are frequently mixed; these sections should be clearly separated. Interpretations and comparisons with the literature should be moved to the Discussion section.

The overall flow from Materials and Methods to Results and Conclusion needs to be clarified and logically structured.

The manuscript editing does not follow the PLOSOne standard.

c) Methodological issues

There are important concerns regarding the methodology:

i. The formula used to calculate the diversity index appears to be incorrect and must be carefully checked and corrected.

ii. The diversity index has been calculated using quantitative data without clear categorization. Typically, diversity indices are applied to qualitative (categorical) data.

iii. If applied to quantitative data, appropriate categorization is required, and the interpretation should reflect distribution properties such as evenness or dominance. The current interpretation is therefore not appropriate and should be revised.

d) Clarity and conciseness

The manuscript contains a significant amount of unnecessary or redundant information, particularly in materials and methods results Please revise these sections to focus on essential information that directly supports the objectives and findings of the study.

Furthermore, some parts of the methodology are not clearly described; please provide clear and logically organized methodological steps. Present results clearly and separately from discussion.

e) . Referencing issues

Citations are numbered in the text, but the reference list is not correspondingly numbered. This makes it difficult to verify sources and follow the scientific argument.

Please ensure consistency and proper formatting of all references.

Final Remark: I have included detailed, specific comments directly within the annotated manuscript to guide your revision. Your study has strong potential, but substantial improvements in writing, structure, and methodological accuracy are required to make the work clear, rigorous, and suitable for publication.

**********

6. PLOS authors have the option to publish the peer review history of their article (what does this mean?). If published, this will include your full peer review and any attached files.

If you choose “no”, your identity will remain anonymous but your review may still be made public.

**Do you want your identity to be public for this peer review?**  For information about this choice, including consent withdrawal, please see our Privacy Policy.

Reviewer #1: **Yes:** Dr. Jyoti Prakash Sahoo

Reviewer #2: **Yes:** Anoumaa Mariette

**********

[NOTE: If reviewer comments were submitted as an attachment file, they will be attached to this email and accessible via the submission site. Please log into your account, locate the manuscript record, and check for the action link "View Attachments". If this link does not appear, there are no attachment files.]

To ensure your figures meet our technical requirements, please review our figure guidelines: https://journals.plos.org/plosone/s/figures

You may also use PLOS’s free figure tool, NAAS, to help you prepare publication quality figures: https://journals.plos.org/plosone/s/figures#loc-tools-for-figure-preparation.

NAAS will assess whether your figures meet our technical requirements by comparing each figure against our figure specifications.

Attachment Submitted filename: PONE-D-26-11665_Rev-AM.pdf

Attachment Submitted filename: PONE-D-26-11665_Comments to the Editor.docx

Attachment Submitted filename: PONE-D-26-11665_comments to author.docx

# Author comment: Author response to Decision Letter 1

**DOI:** 10.1371/journal.pone.0354479.r002

19 May 2026

Response to Reviewer 1

1. The Shannon–Weaver diversity index values for quantitative traits reported in the abstract were verified and corrected accordingly.

2. The yield unit was standardized to t/ha, and the repeated manuscript title was removed from the keywords section.

3. Additional relevant references were incorporated to support the gene pool seed weight threshold statement. Furthermore, the Nepal bean area and production statistics were updated using the latest available data from FAOSTAT (2024), replacing the previous 2023 dataset.

4. Details regarding the augmented block design, including the number of blocks and check varieties, were clarified. The manuscript also specifies that five plants per accession were sampled for quantitative trait evaluation and clarifies the nature of the replicates used.

5. The high thousand-seed weight observed for accession CO14570 (1236 g) was verified and retained, as the accession also exhibited comparatively larger seed dimensions, including seed length (20.35 mm), seed breadth (13.60 mm), and seed thickness (9.06 mm), as documented in the data repository.

6. The modified CTAB DNA extraction protocol was briefly described for clarity. Additionally, PCR annealing temperatures for each primer were included in the revised manuscript in the supplementary information section.

7. In Table 2, some H′ values appear similar due to rounding effects, and this clarification has been added to the manuscript. The PCA section was also revised to clearly state that principal components with eigenvalues greater than 1 (PC1–PC5) were retained for analysis.

8. During molecular data analysis, three monomorphic SSR markers were excluded, and only the 12 polymorphic markers were retained for further analysis.

9. In the discussion section, the observation of H₀ > Hₑ was further explained by considering the possible presence of null alleles as an alternative explanation.

10. The conclusion section was improved to better describe the performance of the evaluated landraces. The revised statement now indicates that several landraces outperformed the three check varieties for important reproductive and yield-related traits, including earliness and yield components.

11. The dataset has been deposited in a public Zenodo repository. In addition, the number of references was expanded from 100 to 112, and all references were reformatted according to the PLOS ONE journal guidelines.

Response to Reviewer 2

1. The manuscript language was thoroughly revised to improve grammatical accuracy, readability, and overall writing quality.

2. The organization of the manuscript was improved, and the Results and Discussion sections are now clearly separated.

3. The formula used to calculate the Shannon–Weaver diversity index was carefully rechecked and corrected where necessary.

4. The interpretation of the H′ diversity index for both quantitative and qualitative traits was clarified. For quantitative traits, a high H′ value indicates an even distribution of accessions across SD-based classes, whereas a low H′ value indicates concentration within a narrow phenotypic range. For qualitative traits, a high H′ value reflects an even distribution across multiple descriptor states, while a low H′ value indicates dominance of one or a few phenotypic classes.

5. Citation numbering within the manuscript was corrected, and all in-text citations were appropriately linked to the corresponding reference list entries.

6. Redundant and unnecessary information, particularly within the Materials and Methods and Results sections, was removed or revised. The methodology was rewritten in a clearer and more logically organized manner, and the Results section was distinctly separated from the Discussion section.

Attachment Submitted filename: Response to Reviewers.docx

# Peer review: Decision Letter 1

**DOI:** 10.1371/journal.pone.0354479.r003

**Authors:** Tzen-Yuh Chiang

8 Jul 2026

Morphological and Molecular Characterization of Nepalese Common Bean (Phaseolus vulgaris L.) Landraces

PONE-D-26-11665R1

Dear Dr. Thapa,

We’re pleased to inform you that your manuscript has been judged scientifically suitable for publication and will be formally accepted for publication once it meets all outstanding technical requirements.

Within one week, you’ll receive an e-mail detailing the required amendments. When these have been addressed, you’ll receive a formal acceptance letter and your manuscript will be scheduled for publication.

An invoice will be generated when your article is formally accepted. Please note, if your institution has a publishing partnership with PLOS and your article meets the relevant criteria, all or part of your publication costs will be covered. Please make sure your user information is up-to-date by logging into Editorial Manager at Editorial Manager® and clicking the ‘Update My Information' link at the top of the page. For questions related to billing, please contact billing support.

If your institution or institutions have a press office, please notify them about your upcoming paper to help maximize its impact. If they’ll be preparing press materials, please inform our press team as soon as possible -- no later than 48 hours after receiving the formal acceptance. Your manuscript will remain under strict press embargo until 2 pm Eastern Time on the date of publication. For more information, please contact onepress@plos.org.

Kind regards,

Tzen-Yuh Chiang

Academic Editor

PLOS One

Additional Editor Comments (optional):

Reviewers' comments:

Reviewer's Responses to Questions

**Comments to the Author**

1. If the authors have adequately addressed your comments raised in a previous round of review and you feel that this manuscript is now acceptable for publication, you may indicate that here to bypass the “Comments to the Author” section, enter your conflict of interest statement in the “Confidential to Editor” section, and submit your "Accept" recommendation.

Reviewer #1: All comments have been addressed

**********

2. Is the manuscript technically sound, and do the data support the conclusions?

The manuscript must describe a technically sound piece of scientific research with data that supports the conclusions. Experiments must have been conducted rigorously, with appropriate controls, replication, and sample sizes. The conclusions must be drawn appropriately based on the data presented.

Reviewer #1: Yes

**********

3. Has the statistical analysis been performed appropriately and rigorously?

Reviewer #1: Yes

**********

4. Have the authors made all data underlying the findings in their manuscript fully available?

The PLOS Data policy requires authors to make all data underlying the findings described in their manuscript fully available without restriction, with rare exception (please refer to the Data Availability Statement in the manuscript PDF file). The data should be provided as part of the manuscript or its supporting information, or deposited to a public repository. For example, in addition to summary statistics, the data points behind means, medians and variance measures should be available. If there are restrictions on publicly sharing data—e.g. participant privacy or use of data from a third party—those must be specified.

Reviewer #1: Yes

**********

5. Is the manuscript presented in an intelligible fashion and written in standard English?

PLOS ONE does not copyedit accepted manuscripts, so the language in submitted articles must be clear, correct, and unambiguous. Any typographical or grammatical errors should be corrected at revision, so please note any specific errors here.

Reviewer #1: Yes

**********

6. Review Comments to the Author

Please use the space provided to explain your answers to the questions above. You may also include additional comments for the author, including concerns about dual publication, research ethics, or publication ethics. (Please upload your review as an attachment if it exceeds 20,000 characters)

Reviewer #1: Thank you for thoroughly addressing all the comments and suggestions from the previous review round. The manuscript has been significantly improved, and your detailed revisions have greatly enhanced its overall quality and clarity. The paper is now scientifically sound and ready for publication, pending a final round of minor grammatical and typographical corrections. I congratulate you on an excellent piece of work.

**********

7. PLOS authors have the option to publish the peer review history of their article (what does this mean?). If published, this will include your full peer review and any attached files.

If you choose “no”, your identity will remain anonymous but your review may still be made public.

**Do you want your identity to be public for this peer review?**  For information about this choice, including consent withdrawal, please see our Privacy Policy.

Reviewer #1: No

**********

# Editor report: Acceptance letter

**DOI:** 10.1371/journal.pone.0354479.r004

**Authors:** Tzen-Yuh Chiang

PONE-D-26-11665R1

PLOS One

Dear Dr. Thapa,

I'm pleased to inform you that your manuscript has been deemed suitable for publication in PLOS One. Congratulations! Your manuscript is now being handed over to our production team.

At this stage, our production department will prepare your paper for publication. This includes ensuring the following:

* All references, tables, and figures are properly cited

* All relevant supporting information is included in the manuscript submission,

* There are no issues that prevent the paper from being properly typeset

You will receive further instructions from the production team, including instructions on how to review your proof when it is ready. Please keep in mind that we are working through a large volume of accepted articles, so please give us a few days to review your paper and let you know the next and final steps.

Lastly, if your institution or institutions have a press office, please let them know about your upcoming paper now to help maximize its impact. If they'll be preparing press materials, please inform our press team within the next 48 hours. Your manuscript will remain under strict press embargo until 2 pm Eastern Time on the date of publication. For more information, please contact onepress@plos.org.

You will receive an invoice from PLOS for your publication fee after your manuscript has reached the completed accept phase. If you receive an email requesting payment before acceptance or for any other service, this may be a phishing scheme. Learn how to identify phishing emails and protect your accounts at https://explore.plos.org/phishing.

If we can help with anything else, please email us at customercare@plos.org.

Thank you for submitting your work to PLOS ONE and supporting open access.

Kind regards,

PLOS ONE Editorial Office Staff

on behalf of

Dr. Tzen-Yuh Chiang

Academic Editor

PLOS One
